# Epigenetic plasticity cooperates with cell-cell interactions to direct pancreatic tumorigenesis

**DOI:** 10.1126/science.add5327

**Published:** 2023-05-12

**Authors:** Cassandra Burdziak, Direna Alonso-Curbelo, Thomas Walle, José Reyes, Francisco M. Barriga, Doron Haviv, Yubin Xie, Zhen Zhao, Chujun Julia Zhao, Hsuan-An Chen, Ojasvi Chaudhary, Ignas Masilionis, Zi-Ning Choo, Vianne Gao, Wei Luan, Alexandra Wuest, Yu-Jui Ho, Yuhong Wei, Daniela F Quail, Richard Koche, Linas Mazutis, Ronan Chaligné, Tal Nawy, Scott W. Lowe, Dana Pe’er

**Affiliations:** 1 Computational and Systems Biology Program, Sloan Kettering Institute, Memorial Sloan Kettering Cancer Center; New York, NY 10065, USA; 2 Tri-Institutional Training Program in Computational Biology and Medicine, Weill Cornell Medicine; New York, NY 10065, USA; 3 Cancer Biology and Genetics Program, Sloan Kettering Institute, Memorial Sloan Kettering Cancer Center; New York, NY 10065, USA; 4 Institute for Research in Biomedicine (IRB Barcelona), The Barcelona Institute of Science and Technology; Barcelona 08028, Spain; 5 Clinical Cooperation Unit Virotherapy, German Cancer Research Center (DKFZ); Heidelberg 69120, Germany; 6 Department of Medical Oncology, National Center for Tumor Diseases; Heidelberg University Hospital, Heidelberg 69120, Germany; 7 German Cancer Consortium (DKTK); Heidelberg 69120, Germany; 8 Department of Pathology, Molecular and Cell-Based Medicine, Icahn School of Medicine at Mount Sinai; New York, NY 10029, USA; 9 Department of Biomedical Engineering, Columbia University; New York, NY 10027, USA; 10 Alan and Sandra Gerry Metastasis and Tumor Ecosystems Center; Memorial Sloan Kettering Cancer Center, New York 10065, NY, USA; 11 Rosalind and Morris Goodman Cancer Institute, McGill University; Montreal, QC H3A 1A3, Canada; 12 Center for Epigenetics Research, Memorial Sloan Kettering Cancer Center; New York, NY 10065, USA; 13 Institute of Biotechnology, Life Sciences Centre; Vilnius University, Vilnius LT 02158, Lithuania; 14 Howard Hughes Medical Institute; Chevy Chase, MD 20815, USA

## Abstract

**Introduction:**

Virtually all cancers begin with genetic alteration in healthy cells, yet mounting evidence suggests that non-genetic events such as environmental signaling play a crucial role in unleashing tumorigenesis. In the pancreas, epithelial cells harboring an activating mutation in the *Kras* proto-oncogene can remain phenotypically normal until an inflammatory event, which drives cellular plasticity and tissue remodeling. The inflammation-driven molecular, cellular, and tissue changes that precede and direct tumor formation remain poorly understood.

**Rationale:**

Understanding tumorigenesis requires a high-resolution view of events spanning cancer progression. We leveraged genetically engineered mouse models (GEMMs), single-cell genomic (RNA-seq and ATAC-seq) and imaging technologies to measure pancreatic epithelial cell-states across physiological, premalignant, and malignant stages. To analyze this rich and complex dataset, we developed computational approaches to characterize epigenetic plasticity and to infer cell-cell communication impacts on tissue remodeling.

**Results:**

Our data revealed that early in tumorigenesis, *Kras*-mutant cells are capable of acquiring multiple highly reproducible cell-states that are undetectable in normal or regenerating pancreata. Several such states align with experimentally validated cells-of-origin of neoplastic lesions, some of which display a high degree of plasticity upon inflammatory insult. These diverse *Kras*-mutant cell populations are defined by distinct chromatin accessibility patterns and undergo inflammation-driven cell fate transitions that precede pre-neoplastic and premalignant lesion formation. Furthermore, a subset of early *Kras*-mutant cell-states exhibit marked similarity to either the benign or malignant fates that emerge weeks to months later; for instance, *Kras*-mutant *Nestin-*positive progenitor-like cells display accessible chromatin near genes active in malignant tumors.

We defined and quantified epigenetic plasticity as the diversity in transcriptional phenotypes that is enabled or restricted by a given epigenetic accessibility landscape. Intriguingly, these plastic cell-states are enriched for open chromatin near cell-cell communication genes encoding ligands and cell-surface receptors, suggesting an increased propensity to communicate with the microenvironment. Given the rapid remodeling of both the epithelial and immune compartments during inflammation, we hypothesize that this epigenetically enabled communication is a major driver of tumorigenesis. We found that the premalignant epithelium displays extraordinary modularity with respect to communication gene co-expression patterns; distinct cell subpopulations each express a unique set of receptors and ligands that define the nature of incoming and outgoing signals that they can receive and send.

Through the development of Calligraphy, an algorithm that utilizes this receptor-ligand modularity to robustly infer the cell-cell communication underlying tissue remodeling, we showed that the enhanced signaling repertoire of early neoplastic tissue specifically endows plastic epithelial populations with greater capability for crosstalk, including numerous communication routes with immune populations. As one example, we identified a feedback loop between inflammation-driven epithelial and immune cell-states involving IL-33, previously implicated in pancreatic tumorigenesis. Using a new GEMM that enables spatiotemporally controlled suppression of epithelial *Il33* expression during *Kras*-initiated neoplasia, we functionally demonstrated that the loop initiated by epithelial IL-33 directs exit from a highly plastic inflammation-induced epithelial state, enabling progression towards typical neoplasia.

**Conclusion:**

Multimodal single-cell profiling of tumorigenesis in mouse models identified the cellular and tissue determinants of pancreatic cancer initiation, and a rigorous quantification of plasticity enabled the discovery of plasticity-associated gene programs. We found that *Kras*-mutant subpopulations markedly increase epigenetic plasticity upon inflammation, reshaping their communication potential with immune cells, and establishing aberrant cell-cell communication loops that drive their progression towards neoplastic lesions.

## Abstract (summary)

The response to tumor-initiating inflammatory and genetic insults can vary amongst morphologically indistinguishable cells, suggesting yet uncharacterized roles for epigenetic plasticity during early neoplasia. To investigate the origins and impact of such plasticity, we performed single-cell analyses on normal, inflamed, pre-malignant, and malignant tissues in autochthonous models of pancreatic cancer. We reproducibly identified heterogeneous cell-states that are primed for diverse late-emerging neoplastic fates and linked these to chromatin remodeling at cell-cell communication loci. Using an inference approach, we revealed signaling gene modules and tissue-level crosstalk, including a neoplasia-driving feedback loop between discrete epithelial and immune cell populations that was validated in mice. Our results uncover a neoplasia-specific tissue remodeling program that may be exploited for pancreas cancer interception.

## One-Sentence Summary

Single-cell analysis reveals that enhanced epigenetic plasticity drives pro-neoplastic crosstalk in early pancreatic tumors.

## Graphical Abstract

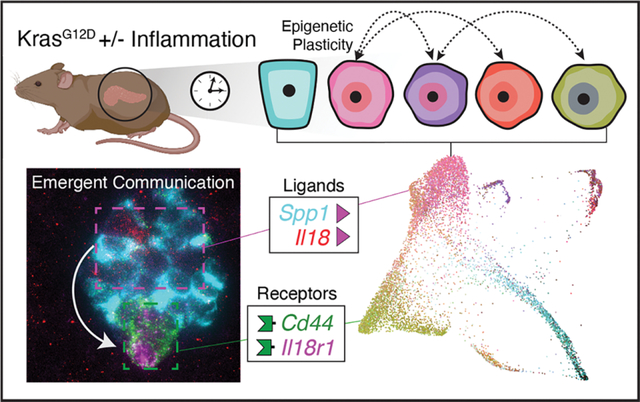

The initial events by which tissues diverge from normalcy to form benign neoplasms and malignant tumors remain poorly understood. It is well established that this process is driven by genetic mutations (1); however, the discovery of prevalent cancer driver mutations in phenotypically normal epithelia (2) challenges the classic notion of cancer pathogenesis and underscores the essential role of cellular and environmental context (3–5). Indeed, non-mutagenic environmental insults promote tumor initiation in mice (6, 7) and chronic inflammatory conditions substantially increase cancer risk in humans (8, 9). These events can have heterogeneous effects even amongst morphologically indistinguishable and genetically identical cells from the same tissue (10). Genetic tracing studies similarly reveal that all such cells are not equally prone to undergo neoplastic and malignant reprogramming (11). This heterogeneity suggests that for tumorigenesis to proceed, select mutant cells either possess or gain an enhanced ability to change cell-states, a phenomenon known as cellular plasticity (12, 13).

Developmental, regenerative, and pathologic plasticity is largely determined at the chromatin level as increases or decreases in the repertoire of transcriptional programs that can be accessed by a given cell (13, 14). Cells showing a high degree of plasticity, such as stem cells, often have a more ‘open’ or accessible chromatin landscape that becomes restricted during differentiation (15, 16). Previous work has used de-differentiation with respect to normal cell-states to characterize cancer cell plasticity with single-cell genomics from lung cancer models (17, 18). However, we still do not know how plasticity emerges in the earliest stages of tumorigenesis, particularly in concert with the environmental insults that accelerate these initiating events. Learning how plasticity is triggered to arise in pre-malignant tissues and how it contributes to early tumor evolution is paramount to understanding and intercepting cancer at its earliest stages.

Pancreatic ductal adenocarcinoma (PDAC) is typically diagnosed too late for curative treatment and arises from cooperativity between genetic and epigenetic reprogramming events (19). Unlike more genetically heterogeneous cancers, PDAC is invariably initiated by an activating mutation in the proto-oncogene *KRAS.* However, *KRAS*-mutant epithelia can remain phenotypically normal and depend on inflammatory stimuli (pancreatitis) to transform into pre-neoplastic and neoplastic lesions (20–22). We (23) and others (24, 25) have reported that oncogenic KRAS, in the absence of further mutation, cooperates with inflammation to trigger large-scale chromatin remodeling events that promote tumor initiation. However, important questions remain: How does *KRAS*-mediated plasticity give rise to neoplastic lineages and enable their subsequent evolution to invasive disease? What are critical cell-intrinsic and cell-extrinsic factors that determine a cell’s propensity to acquire a plastic and ultimately a tumorigenic cell-state? Understanding the answers to these questions may point to intervention strategies to prevent PDAC progression.

To shed light on neoplastic plasticity in PDAC, we compared physiological, pre-malignant, and malignant cell-state heterogeneity using single-cell genomics, applying computational methods and functional perturbation in autochthonous genetically engineered mouse models (GEMMs) that accurately recapitulate many aspects of the human disease. Beyond providing a comprehensive charting of epithelial dynamics from normal metaplasia through malignant tissue states, our approach allowed us to expose, quantify, and perturb early plasticity traits endowed by oncogene-environment interaction, and define molecular, cellular, and tissue-level principles of pre-malignant tumor evolution.

## Results

### Targeted high-resolution profiling of epithelial dynamics during damage-induced neoplasia

**Figure 1.**
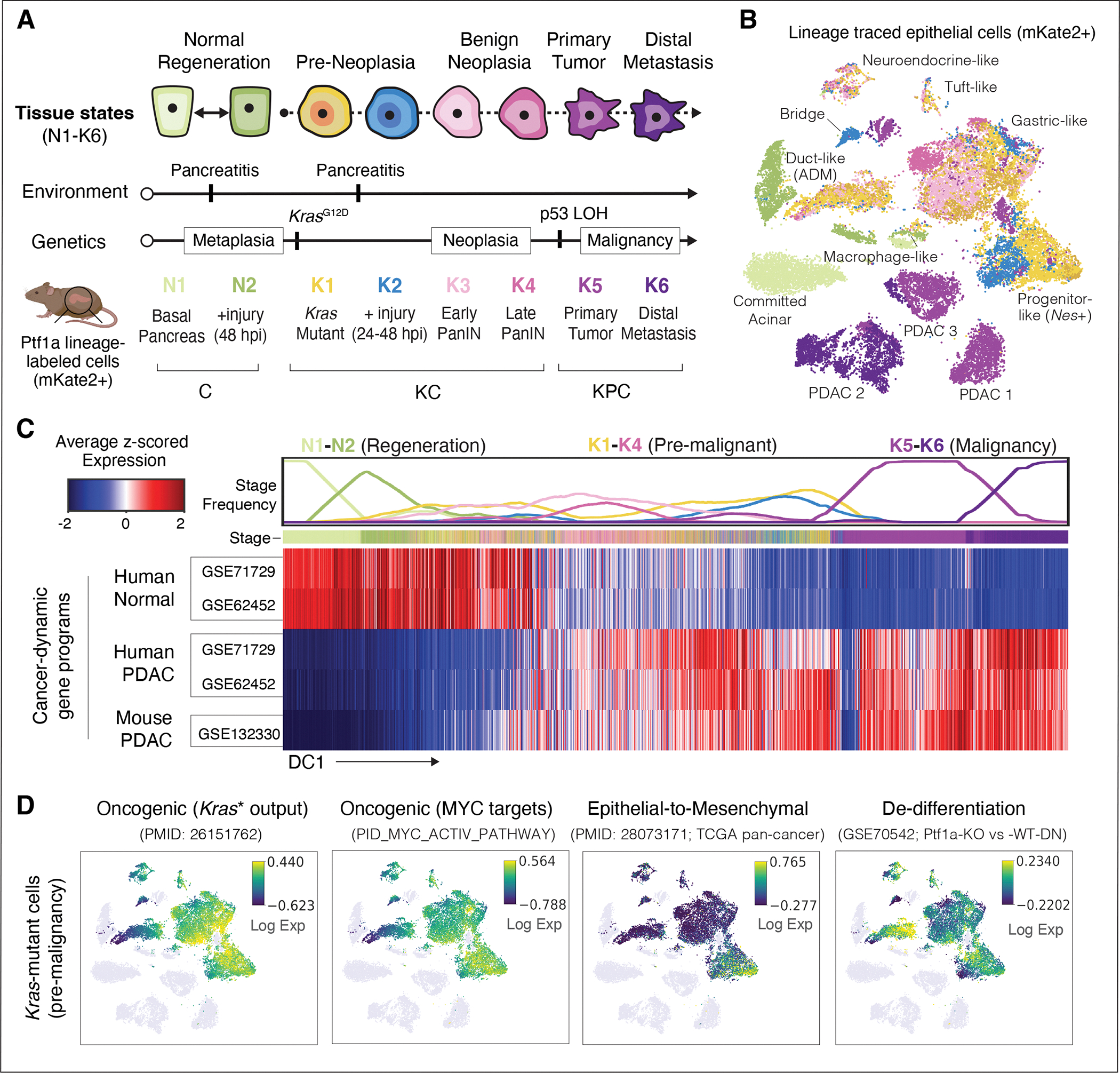
A single-cell transcriptomic atlas of pancreatic regeneration and tumorigenesis. (**A**) Experimental design for tissue collection. GEMMs expressing *Ptf1a*-Cre enable FACS-based enrichment of mKate2-labeled exocrine pancreas epithelial cells (23). mKate2^+^ cells were isolated from wild-type *Kras* mice before injury with caerulein (N1) or 48 hours post-injury (N2); and from *Kras*^*G12D*^ mice (KC genotype) before injury (K1), and 24–48 hours (K2) or 3 weeks after caerulein (K3, PanIN stage), as well as uninjured older KC mice (K4). PDAC primary tumors (K5) and liver and lung metastases (K6) were harvested from KC mice with a *p53* floxed (*p53*^*fl/+*^) or mutant (*p53*^*R172H/+*^) allele (KPC genotype). Mouse illustration was created with BioRender (https://biorender.com/). (**B**) tSNE visualization of pancreatic epithelial scRNA-seq profiles from all collected stages (n = 17 mice), colored as in (A) and labeled by cell-state (27). ADM denotes cells undergoing acinar-to-ductal metaplasia (31), and ‘Bridge’ denotes cells between acinar-like and malignant programs, which express genes from both. (**C**) Expression of PDAC-associated gene sets (rows) across all pancreatic epithelial cells (columns) (34, 35). Cells are ordered by the first diffusion component (DC1), representing the major axis of progression from normal (N1) to metastatic (K6) states. Plot at top displays frequency (from 0 to 1) of cells per stage, in bins of n = 2000 cells. Gene set score for each cell is computed as the average of log-normalized expression, z-scored for each gene to obtain a comparable scale. Heatmap is standardized to compare cells within each gene set. (**D**) tSNE plots as in (B), with pre-malignant (K1–K4) *Kras*-mutant cells colored by the expression of genes (from left to right) upregulated in bulk RNA-seq of *Kras-*mutant (Kras*) pancreas relative to normal (67), associated with Myc activity (68), EMT (36), or down-regulated upon *Ptf1a* knockout (67). Colors are scaled from 5th to 95th percentile of expression.

The study of epithelial dynamics in pancreatic cancer has been limited by the inability to capture early and transitional cell-states, which tend to be rare, short-lived, and difficult to identify. To characterize the full spectrum of epithelial cell-states in both normal and pathological tissue remodeling, we generated a single-cell transcriptomic (scRNA-seq) atlas of healthy, regenerating, benign neoplastic, and malignant epithelia using GEMMs that faithfully model cancer from initiation to metastasis. Our GEMMs incorporate a *Ptf1a*-Cre-dependent mKate2 fluorescent reporter to enrich pancreatic epithelial cells (23, 26, 27), allowing us to comprehensively profile pancreatic epithelial dynamics in well-defined tissue states. Specifically, we profiled pancreatic epithelial cells from healthy pancreas (N1) undergoing reversible metaplasia associated with normal regeneration after injury (N1→N2), and the metaplasia-neoplasia-adenocarcinoma sequence that initiates PDAC in the presence of mutant *Kras* (K1→K6) (Figs. 1A, S1A and Table S1). In this setting, as in human cancer (10), *Kras*-mutant metaplasia is accelerated by an inflammatory insult (pancreatitis) (pre-neoplasia; K1→K2), proceeds to benign pancreatic intraepithelial neoplasia, (PanIN; K3, K4), and ultimately, malignant PDAC (K5) and distal metastases (K6; Figs. 1A and S1A).

Using a lineage tagging reporter to enrich for epithelial (mKate2^+^) cells, we captured both abundant and rare constituents of normal, regenerating, and *Kras*-mutant epithelia, such as progenitor-like tuft (*Pou2f3*^+^, *Dclk1*^+^), EMT-like (*Zeb1*^+^), neuroendocrine (*Syp*^+^ *Chga*^+^), and other previously reported subpopulations (28–30) (Figs. 1B, S1B–E and Table S2). We also characterized highly granular routes of acinar-to-ductal metaplasia (ADM) associated with regeneration and tumor initiation (23, 31) (Fig. S2A,B). Compared to healthy and regenerating pancreata, we uncovered a staggering expansion of new cell-states that emerge during the earliest stages of KRAS-driven tumorigenesis (K1-K2, Figs. 1B, S1B–E and S2A–C). Despite such heterogeneity, the distinct cell-states captured within pre-malignant tissues were reproducible across biological replicates (individual mice) (Fig. S2D). In stark contrast, and consistent with studies analyzing human PDAC (32), malignant tumors isolated from different mice showed extensive inter-tumor variability, only sharing one small cell cluster (Fig. S2D,E).

We next used diffusion maps (33) to characterize the major axes of transcriptional variation in our data, ordering cells along components associated with coherent gene expression patterns. The top component of variation closely matches progression from normal to regenerating, early tumorigenic, and finally late-stage disease, and is consistent with gene signatures that distinguish advanced human PDAC from normal pancreas (34, 35). Specifically, genes upregulated in human and mouse PDAC rise along the first diffusion component (DC), while normal pancreas programs are downregulated (Fig. 1C). Consistent with prior reports analyzing bulk RNA-seq data (23), the combined effects of *Kras* mutation and injury-driven inflammation are sufficient to induce signatures of human PDAC in pre-malignancy, as early as 24 to 48 hours post-injury (hpi) (Fig. 1C,D). However, the added granularity of our single cell analyses revealed that cancer-specific signatures are not induced uniformly across pre-malignant epithelial cell-states; for example, some rare early *Kras*-mutant cells express high levels of EMT gene programs (*Zeb1*, *Vim;* (30, 36)) (Figs. 1D and S1E). *Kras*-mutant cell-states are also observed with varying degrees of de-differentiation (downregulation of acinar genes) and reactivation of developmental (*Clu*) or oncogenic (*Kras, Myc*) programs (Fig. 1D). Thus, in the presence of inflammation, *Kras-*mutant pancreatic epithelial cells rapidly undergo specific and highly reproducible changes that endow select subpopulations with the capacity to activate disease-relevant programs long before malignant progression.

### Aberrant, highly plastic cell-states emerge early in PDAC progression

**Figure 2.**
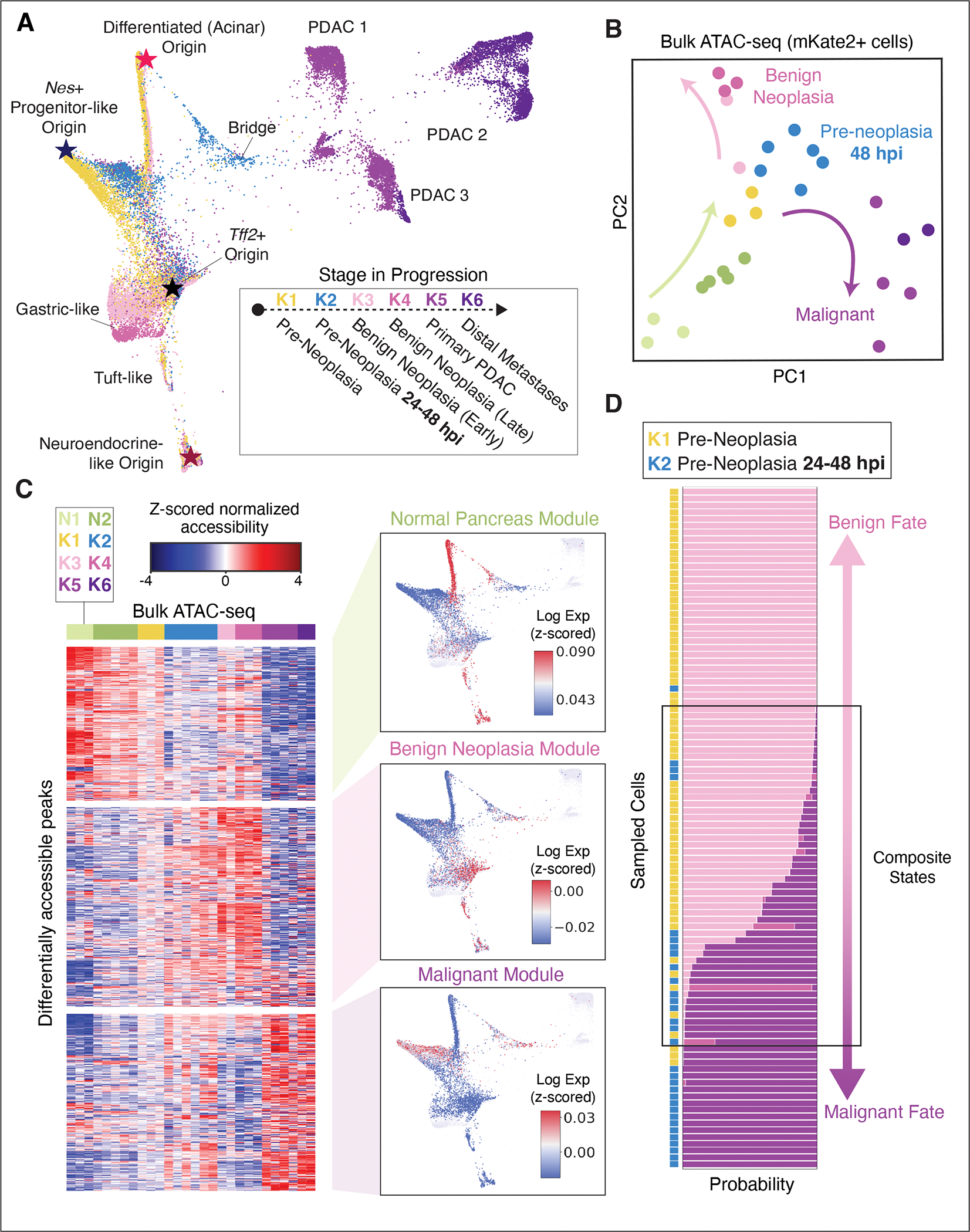
Differential epigenetic priming of *Kras*-mutant cells. (**A**) Force-directed layout (FDL) of all *Kras*-mutant scRNA-seq profiles (K1–K6, n = 11 mice). Cells colored by stage as in Fig. 1A. Stars highlight ‘apex’ states inferred by CellRank (39) (see Fig. S3B). (**B**) Principal component analysis (PCA) of bulk ATAC-seq profiles from pancreatic epithelial cells. Each point shows the position of a single biological replicate (individual mouse), colored by stage as in (A). Arrows indicate a transition upon injury and *Kras* mutation (N1-N2, K1-K2; green arrow) and a divergence between benign neoplastic (K3-K4; pink arrow) and malignant (K5-K6; purple arrow) stages. (**C**) *Left*: Chromatin accessibility along progression. Subsets of differentially accessible ATAC-seq peaks (rows) are organized into three modules by clustering (27); bulk ATAC-seq replicates (columns) are ordered and colored by stage as in (A). Peaks organize into distinct accessibility patterns, denoted as chromatin modules (27). *Right*: Expression of genes corresponding to chromatin accessibility modules in pre-neoplastic cells (K1, K2). FDL map as in (A), colored by module expression score computed by z-scoring each cell to emphasize dominant gene programs per cell, and averaging genes nearest to module peaks. Color (expression scores) are scaled between the 40^th^ and 90^th^ percentiles. (**D**) Probability of classifying pre-neoplastic cells (K1, K2) as more similar to benign neoplastic (K3-K4) or malignant (K5-K6) scRNA-seq profiles, based on expression similarity. Sampled cells (rows) are ordered from highest benign fate probability (top) to highest malignant fate probability (bottom); bars represent probability of classification from 0 to 1 to K3, K4, K5, or K6 labels, colored as in (A). A fraction of cells exhibit composite states with probability for both fates.

To map the cellular origins and processes underlying this diversity in transcriptional cell-states, we first visualized heterogeneity in all *Kras*-mutant epithelia using a force-directed layout (FDL), which emphasizes cell-state transitions along axes toward malignancy. As expected, we found that the *Kras-*mutant pancreatic epithelium undergoes progressive gene expression changes that activate metaplastic (*Clu*^+^*, Krt19*^+^; (22)), neoplastic (*Agr2*^+^, *Muc5ac*^+^*, Tff1*^+^; (37)) and ultimately, invasive cancer (*Foxa1*^+^; (38)) programs (Fig. 2A). The relatively low heterogeneity in apoptotic or proliferative signatures (Fig. S3A) implies that much of the change in observed cellular states is likely due to cell-state transitions rather than population dynamics.

To better characterize sources of cell-state variation, we applied CellRank (39), a data-driven approach that infers transcriptional dynamics from cell-cell similarity coupled with RNA velocity information (27, 40, 41). RNA velocities derived from the proportion of spliced to unspliced transcripts in each cell can indicate likely future states in neighboring phenotypic space. CellRank integrates directional information from per-cell velocity estimates with standard pseudotime inference based on cell-cell similarity to infer global transcriptional dynamics that can robustly pinpoint the origins of cell-state trajectories (39). Applying CellRank to early *Kras*-mutant cells acutely responding to an inflammatory insult (K1→K2) identified multiple states that potentially act as distinct origins for the observed heterogeneity (Fig. S3B).

The four inferred origin or ‘apex’ states include one differentiated acinar (*Ptf1a*^+^) and three de-differentiated (*Nes*^+^, *Aldh1b1*^+^ and *Tff2*^+^) populations. Most of these states align with independent genetic lineage tracing studies that demonstrate their ‘cell-of-origin’ potential individually (26, 42–45) (Fig. S3B,C). These relationships are further supported by single molecule fluorescence in situ hybridization (smFISH) data derived from inflamed *Kras*-mutant tissue (K2), which revealed clear transitional states (*Anxa10*^+^ *Nes*^+^ *Msn*^+^) in lesions containing both apex cells (*Nes*^+^ *Msn*^+^) and the gastric-like cells (*Anxa10*^+^) predominant in neoplastic tissue weeks later (K3-K4, Fig. S3D,E).

Moreover, several of the inferred apex states are highly responsive to inflammation, with apparent cell-state shifts along the cell-cell similarity graph emerging in the context of tissue injury (K2). For instance, during pancreatitis, well-differentiated acinar cells generate a metaplastic population with transcriptional features that are intermediate for acinar (*Zg16, Cpa1*) and tumorigenesis-associated (*S100a6*) programs within 24 hpi (ADM-PDAC “Bridge”) (Fig. S3F), and *Nes*^+^ progenitor-like cells shift into a state showing reduced activation of tumor suppressive programs (*Cdkn2a*). Our findings thus suggest that oncogenic *Kras* enables the emergence of diverse high-potential states (not observed in healthy nor regenerating pancreata (see Figs. 1B and S2)), each exhibiting distinct responses to inflammatory triggers, but all upregulating cancer-associated programs (see Fig. 1D).

### An epigenetic basis for high plasticity states

Given the important role for chromatin dynamics in driving neoplasia (23), we hypothesized that the expansive phenotypic diversity in *Kras*-mutant apex states and their injury-driven progeny arises through a diversification of permissive chromatin states. To determine how chromatin dynamics correspond to changes observed in our longitudinal scRNA-seq atlas, we first analyzed bulk ATAC (assay for transposase-accessible chromatin) sequencing data matching the above tissue stages (23, 27). The dominant principal components of variation revealed accessibility patterns specific to each stage of progression (Fig. 2B). Compared to samples from normal pancreas epithelium (N1-N2), the chromatin landscapes of pre-neoplastic *Kras*-mutant epithelia (K1-K2) reproducibly shift toward states acquired in early neoplasia and sustained in advanced disease (K3-K6). Nevertheless, we observed that the chromatin landscapes of benign neoplastic lesions (K3-K4) and malignant tumors (K5-K6) are highly divergent. Consistent with this, large sets of regulatory elements (“chromatin modules”) exhibit mutually exclusive accessibility patterns across benign and malignant stages, with one set of ATAC-seq peaks showing increased accessibility in benign lesions but not in malignant counterparts (Benign Neoplasia chromatin module) and another set behaving opposite (Malignant chromatin module, Fig. 2C and Table S3). The modular structure of these data suggests that chromatin accessibility at benign (K3-K4) and malignant (K5-K6) stages corresponds to discrete, stable cell-states, which may underlie the clearly distinct morphologies (see Fig. S1A) and expression patterns (see Fig. S2D) characteristic to cells of these advanced stages.

Mapping diverging Benign Neoplasia and Malignant chromatin accessibility modules to single-cell gene expression reveals a concordant pattern; genes proximal to Benign Neoplasia module loci are up-regulated in pre-malignant disease (K3-K4) and genes proximal to Malignant module loci increase in malignant disease (K5) (Fig. S4A). Chromatin modules associated with advanced stages are induced remarkably early in tumor development, such that Benign neoplasia and Malignant chromatin module associated genes are expressed and restricted to transcriptionally distinct populations within 24–48 hpi (K1-K2, Fig. 2C and Table S3). Furthermore, bulk ATAC-seq data show an initial increase in accessible chromatin in both modules from samples collected at 48 hpi, or without injury (K1-K2)—well before the emergence of benign lesions or malignant disease (K3-K6, Fig. 2C). These observations imply that the transcriptional diversity of pre-neoplastic *Kras-*mutant cells is established at the chromatin level and involves Benign Neoplasia or Malignant module activation prior to the development of PanINs or PDAC. This activation requires both oncogenic *Kras* and inflammation, as these programs are not similarly accessible or expressed in normal regeneration (N1-N2) (Figs. 2C and S4A).

The early establishment of a permissive chromatin landscape (K1-K2) that is later specified into a restricted, distinct set of accessible regulatory elements (K3-K6) is reminiscent of cell-fate determination occurring in developmental systems (16). We thus refer to the cell-states that pre-neoplastic *Kras*-mutant cells may eventually acquire as ‘cell-fates’—those associated with benign neoplasia (K3-K4) or malignancy (K5-K6). We postulated that pre-neoplastic *Kras*-mutant cells (K1, K2) expressing programs associated with the chromatin landscape of a single distinct fate (Benign or Malignant) may be epigenetically primed toward that fate, conferring greater propensity to acquire its phenotype over time or in response to certain exogenous triggers. We further reasoned that similarities between the transcriptomes of *Kras-*mutant cells from pre-neoplastic (K1, K2) and later neoplastic stages (K3–K6) would indicate such fate potential. We therefore developed a classification-based approach that first identifies gene expression patterns that accurately discriminate between cell populations in benign lesions or cancer, and then uses these patterns to assign cell-fate probabilities to pre-neoplastic cells based on the activation of fate-associated genes. Specifically, we trained a logistic-regression classifier to distinguish between benign neoplasia (K3, K4) and malignancy (K5, K6), and used it to classify pre-neoplastic (K1, K2) cells (27). This classifier is highly accurate (99%) in assigning fate to PanIN and PDAC cells of known fate and identified a set of discriminative genes which have been linked to either fate (Fig. S4B).

Applied to pre-neoplastic cells, this approach indeed pinpointed *Kras*-mutant cells that are strongly skewed toward one or the other fate (Figs. 2D and S4C). Most pre-neoplastic cells are classified as only having the potential to acquire a single fate, with cells responding to inflammation (K2) assigned a higher probability of acquiring a malignant fate. We also identified an intriguing set of *Kras*-mutant cells that are not well classified (Figs. 2D and S4D), the majority of which express a composite program of otherwise divergent fate-associated genes (Fig. S4E). These dual-primed subpopulations exist largely in the absence of tissue damage and overlap with initiating apex states (*Ptf1a*^+^ acinar and *Nes*^+^ progenitor) captured independently by CellRank (see Fig. S3B,C).

Collectively, our results imply that tumorigenesis can proceed from multiple well-differentiated or progenitor-like states, and that their neoplastic progression is not dictated solely by cell intrinsic determinants (*Kras* gene mutation) but impacted by inflammatory signals that epigenetically prime them towards diverse fates that can be predicted early in disease progression.

### Epigenetic plasticity is enhanced by inflammation

**Figure 3.**
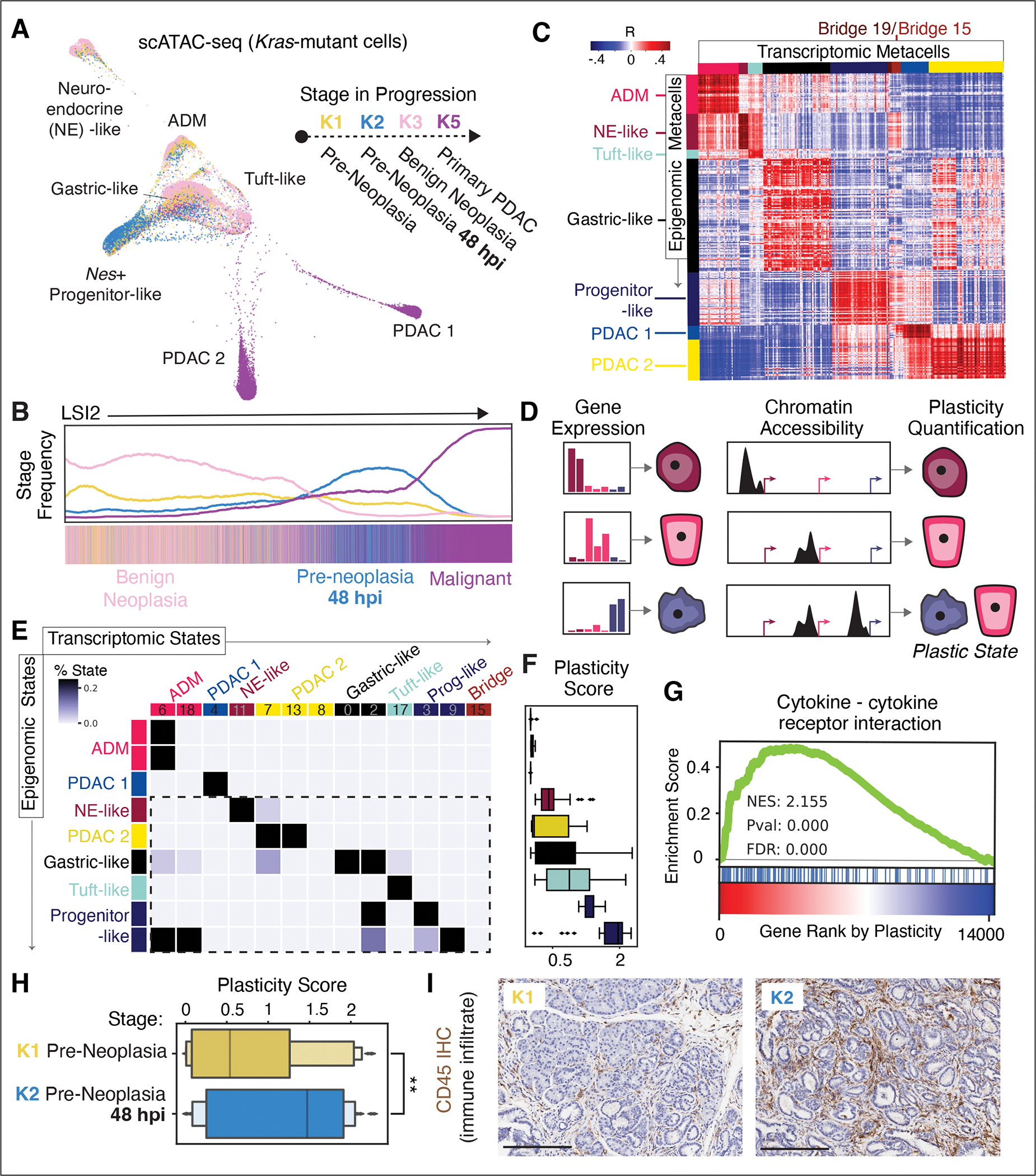
*Kras*-mutant cells display elevated epigenetic plasticity, which is associated with cell-cell communication propensity. (**A**) FDL of scATAC-seq profiles from *Kras-*mutant epithelial cells from pre-malignant (K1–K3) and malignant (K5) stages (n = 9 mice), colored by stage. (**B**) Frequency of cells from each stage along second high-variance component from latent semantic indexing (LSI) of scATAC-seq profiles (65). (**C**) Pairwise Pearson correlation coefficients of metacells from scATAC-seq ArchR gene accessibility scores (rows) and scRNA-seq expression values (columns). Annotated cell-states, determined by refined PhenoGraph clustering of scRNA-seq (Fig. S1C) and scATAC-seq (Fig. S5A), are colored according to their annotation as in labels from (A). Blocks of positive correlation along diagonal represent similar cell-states across the two modalities, whereas off-diagonal correlations indicate similarity across distinct cell-states. (**D**) Cartoon of classifier-based approach to quantify plasticity (27). (**E**) Classifier confusion matrix based on procedure in (D). Cell-states, determined by scRNA-seq (Fig. S1C) and scATAC-seq (Fig. S5A) metacell clusters, are colored as in labels in (A). Values represent number of metacells from an epigenomic cluster that classify to a transcriptomic cluster, normalized within each row. Dashed box highlights high plasticity epigenomic states. (**F**) Plasticity scores for epigenomic clusters in (E). Boxes represent interquartile range (IQR) of plasticity scores for all epigenomic metacells assigned to that cluster, computed as per-cell Shannon entropy in the classifier’s predicted probability distribution across transcriptomic states. Lines represent medians and whiskers represent 1.5x IQR. (**G**) GSEA plot based on Spearman rank correlation between plasticity score and each gene’s accessibility score. (**H**) Plasticity scores for epigenomic metacells from K1 and K2, showing significant increase in plasticity in K2 (one-tailed t-test; t = 2.5511, p value = 0.006). (**I**) Immunohistochemistry of CD45 (brown) marking immune cells in K1 and K2 tissue, showing increase in immune infiltrate in response to injury. Scale bar, 200 μm.

To map the epigenomic landscape at higher resolution, we generated single-cell chromatin accessibility (scATAC-seq) profiles of pre-neoplastic (K1), pre-neoplastic inflamed (K2), benign neoplastic (K3), and adenocarcinoma (K5) epithelia. Consistent with an epigenetic basis for the observed pre-malignant diversity (see Fig. 2C), we found considerable heterogeneity in chromatin accessibility within *Kras*-mutant epithelial cells at each stage (Figs. 3A and S5A). A major axis of variation in accessibility reproduced the divergence between benign and malignant fates seen in bulk analyses (Figs. 3B and S5B). Substantial variation in accessibility near fate-associated genes occurred across both stages and clusters (Fig. S5C), with *Kras*-mutant apex cells exhibiting a composite state defined by open chromatin at benign-associated and malignant-associated loci. This pattern extends to variation in open chromatin near other genes that define the benign and malignant chromatin modules (Fig. S5D). These data support bona fide epigenetic priming of divergent fate-associated programs in early, pre-neoplastic *Kras*-mutant cells.

To better connect primed chromatin landscapes to their transcriptional outputs, we next sought to integrate scATAC-seq and scRNA-seq profiles from comparable stages. Clustering and cell-state annotation demonstrated that cell-states derived from scRNA-seq data largely match those derived from scATAC-seq data at the broad cluster level, including those corresponding to *Nr5a2*^+^ acinar, *Neurod1*^+^ neuroendocrine, *Pou2f3*^+^ tuft, and *Nes*^+^ progenitor cells (Fig. S5A). However, we also found substantial epigenomic heterogeneity within each scATAC-seq cluster. To explore this heterogeneity in more detail, we applied an algorithm that aggregates highly similar cells into granular cell-states, or metacells (27, 46, 47). Metacells provide much higher resolution than clusters, but aggregate cells sufficiently to reduce sparsity and improve statistical power for comparison.

After separately identifying metacells for each scRNA-seq and scATAC-seq modality, we developed a framework to map between them based on similarity between a gene’s expression and its proximal chromatin accessibility (Fig. S6A–C) (27). This integrative analysis showed the expected correspondence between the accessibility and expression programs of comparable cell-states (Fig. 3C). However, we also observed extensive off-diagonal correspondence, indicating that features of the chromatin landscape are shared across diverse gene expression states. Specifically, we found diverse pre-malignant transcriptional states (tuft, neuroendocrine, progenitor, and gastric) to broadly correlate with the ADM epigenomic state, reflecting the known acinar history of these *Ptf1a* lineage-sorted cells (23, 26). In other cases, these correspondences may indicate widespread transcriptional “poising” of regulatory elements near unexpressed genes. Such effects were particularly evident in apex *Nes*^+^ progenitor cells, which exist in pre-neoplastic tissues at 48 hpi but establish chromatin landscapes that are highly correlated with those of late-stage malignant populations (Figs. 3C and S5B). While these similarities can be partially explained by lineage relationships within each data modality, the greater off-diagonal (inter-cell-state) correlation existing across modalities (Fig. S6D) suggests that subpopulations of pre-neoplastic *Kras*-mutant cells are epigenetically primed to engage neoplasia transcriptional programs later in progression (see Fig. 2C).

We sought to quantify the degree of epigenetic plasticity, which we define as the amount of diversity in transcriptional phenotypes that is enabled (or restricted) by a given chromatin accessibility landscape. To first determine these potential transcriptional phenotypes, we used a simple classifier to identify gene expression patterns that discriminate cell-states. Assuming that proximal open chromatin conveys the potential for a gene’s activation, we then applied the classifier to predict cell-states based on accessibility proximal to genes, rather than gene expression (Fig. 3D). We reasoned that for a given epigenomic state, uncertainty in such predictions serves as a measure of epigenetic plasticity. Following this logic, high plasticity is characterized by many accessible loci that define multiple discrete transcriptional states and thus produce high classifier prediction uncertainty. In contrast, low plasticity is defined by restricted potential diversity and prediction certainty. Applying this approach to epigenomic metacells identified populations of varying plasticity (Figs. 3E,F and S6E), with the most plastic states exhibiting striking overlap with the apex cells identified by CellRank (such as *Nes*^+^ progenitors, *Tff2*^+^ gastric cells) and experimentally validated cells-of-origin from lineage tracing studies (such as *Pou2f3*^+^ tuft cells; (28, 42–44)) (Fig. 3F). Some of these states arise largely from pre-neoplastic conditions (K1-K2), aligning with observations on priming toward future neoplastic states. Notably, all plastic states identified in this analysis have no clear analog in normal or regenerating pancreata (see Figs. 1B, S2A,B), although a deeper exploration of physiological plasticity would be required to fully contrast normal and disease mechanisms.

To expose potential unifying features of distinct plastic cell-states, we used gene set enrichment analysis (GSEA) (48) to identify gene signatures within populations displaying high plasticity scores (Table S4). This analysis revealed robust and consistent upregulation of sets related to cell-cell communication (Fig. S6F), with Cytokine-Cytokine Receptor Interaction yielding the top association (normalized enrichment score = 2.155, adjusted p value = 0.000) (Fig. 3G). A substantial fraction of these plasticity-associated genes encoded inflammatory mediators, receptors, or ligands involved in cell-cell communication, including those previously associated with malignant progression (*Csf2*, *Cxcl1*, and *Cxcr2* (49)) (Table S5). Accordingly, plasticity increases significantly (p value = 0.006; one-tailed t-test, t = 2.5511) upon injury in the context of *Kras* mutation (K2 vs. K1) (Fig. 3H), suggesting an interplay between highly plastic cells and immune infiltrates flooding the pre-neoplastic tissue environment in this context (Fig. 3I). Together, our results indicate that epithelial plasticity in pre-malignant cells is directly associated with an increased, epigenetically-encoded propensity for ligand-receptor mediated communication with the immune microenvironment.

### Calligraphy charts cell-state-specific communication repertoires and their interactions

The dominance of the association between plasticity and cell-cell communication drove us to investigate how heterotypic interactions may result from plasticity or enhance it in the *Kras-*mutant pancreatic epithelium. We hypothesized that chromatin remodeling of receptor and ligand gene loci (hereafter, ‘communication genes’) contributes to plasticity in pre-neoplasia by enabling cells to respond to inductive signals from the environment.

**Figure 4.**
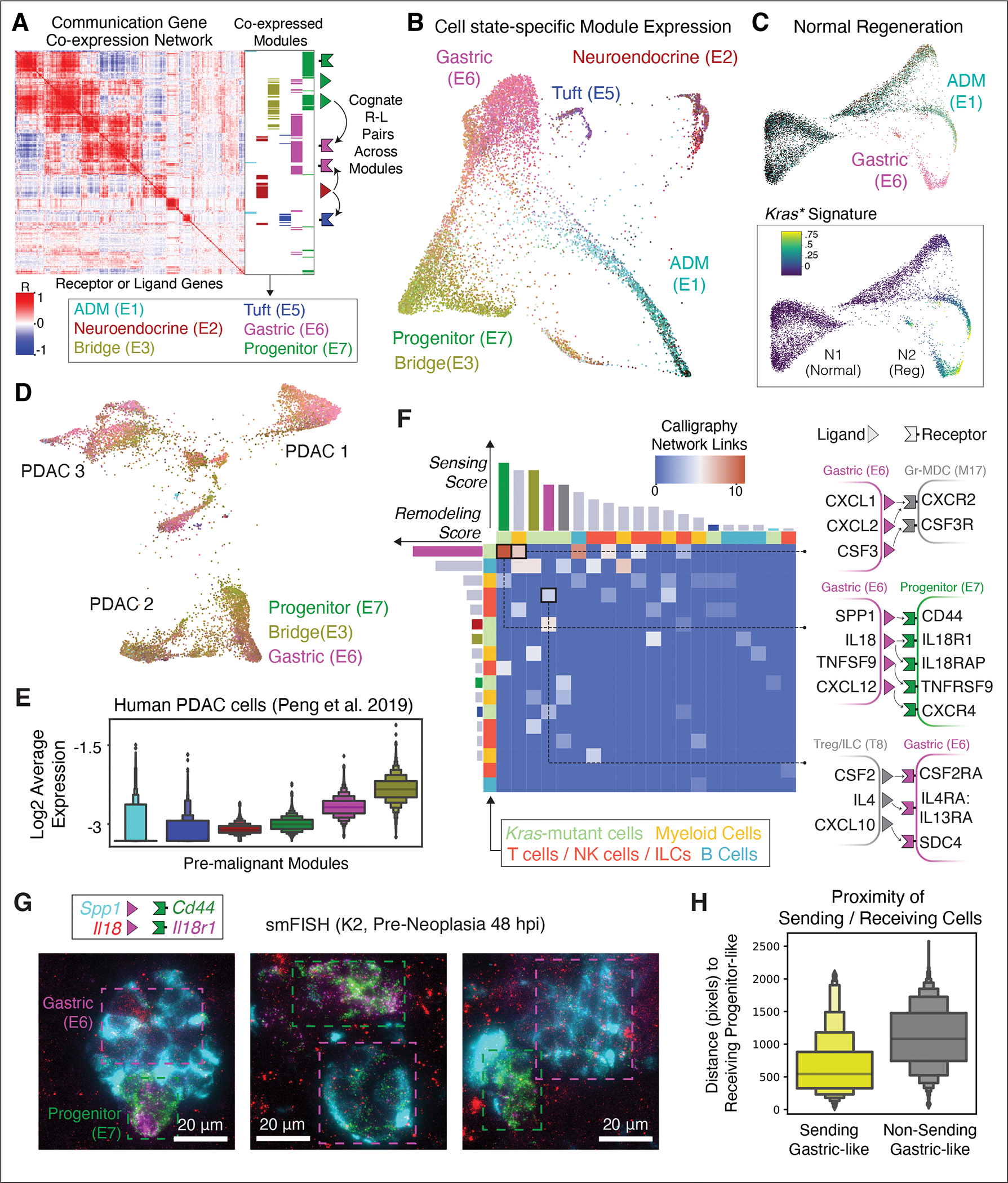
Inferred epithelial-immune crosstalk in plastic neoplastic states. (**A**) Calligraphy-inferred ‘communication modules’ in pre-malignant *Kras-*mutant epithelial cells (K1-K3, n = 6 mice). Each row or column represents one receptor or ligand; value at intersection indicates correlation in expression (Pearson r) of that gene pair across pre-malignant cells. Blocks of highly correlated genes denote partially overlapping modules (annotated at right) that tend to co-express in the same cell-states. Schematic (far right) describes the second step of Calligraphy (see Fig. S11A). (**B**) FDL of K1–K3 epithelial cells with color values based on relative communication module gene expression (27). (**C**) FDL of *Kras*^WT^ pancreas cells before and after injury (N1-N2, n = 4 mice), colored by K1–K3 communication module expression as in (B) (top) or *Kras* mutant signature gene expression (bottom, (23)), scaled between 1st and 99th percentile. (**D**) FDL of malignant cells (K5-K6, n = 3 mice), colored as in (B). (**E**) Communication module expression in human pancreatic tumor scRNA-seq data (32), colored as in (B). (**F**) Pairwise crosstalk between communication modules inferred by Calligraphy from epithelial or immune scRNA-seq data (one module per row or column), colored gray for immune or as in (A) for epithelial modules. Heat values represent number of inferred cognate R-L pair interactions across each communicating module pair; some contributing receptors or ligands are shown at right. Bars quantify total inferred edge counts, representing remodeling (row) or sensing (column) interactions for that module. (**G**) Two smFISH fields of view reveal the spatial proximity of sending (magenta box) to receiving (green box) mKate2^+^ epithelial cells. The expression of two Gastric (E6) module ligands (cyan and red), as well as two Progenitor (E7) receptors (magenta and green) overlap spatially in these three examples. Scale bars, 20 μm. (**H**) Distance between each receiving progenitor cell (*Il18r1*^hi^ *Cd44*^hi^) and double-positive sending gastric cell (*Il18*^hi^ *Spp1*^hi^), versus randomly selected non-sending gastric cells (*Il18*^lo^ *Spp1*^lo^).

The delineation of communication events requires an assessment of the communication propensities of each cell-state (defined by its expressed receptors and ligands), which may then be used to link interacting cell-states based on prior knowledge of receptor-ligand binding partners. As a first step, we characterized communication gene accessibility and expression across cell-states of the pre-malignant epithelium using the scATAC-seq and scRNAseq datasets generated above. Each plastic cell-state reveals substantial variability in chromatin accessibility near communication genes, consistent with distinct molecular repertoires for potential communication (Fig. S7A). To identify trends of coordinated gene expression, we searched for co-expression between any two communication genes (testing all combinations of two receptors, two ligands, and one of each) in individual cells across the pre-malignant epithelium and found a high degree of block structure in pairwise co-expression. This pattern implies that communication capabilities are driven by ‘communication modules’; sets of communication genes that are mutually expressed in the same cell populations (Fig. 4A).

We next sought to infer actual cell-cell signaling interactions that may occur between cells expressing different communication modules. Although several methods have been developed to predict cellular interactions from single-cell data (50), their inference relies on weak signals arising from the noisy expression of a single cognate receptor-ligand (R-L) pair across fixed cell-states. We therefore developed our own approach, Calligraphy, that leverages the observed modularity in communication gene expression to infer potential cell-cell signaling events (27). Calligraphy first identifies communication modules—thereby establishing the incoming and outgoing communication each cell-state can participate in. Next, Calligraphy identifies communication events between cell-states based on prior knowledge of cognate R-L binding partners. Unlike previous methods which test interactions on individual R-L pairs, Calligraphy draws inferences across entire sets of genes, making the output insensitive to noise in any single gene (50).

Using Calligraphy (27), we obtained seven communication modules of genes that are co-expressed across the pre-malignant pancreatic epithelium (Fig. 4A and Table S6) and are reproducible *in situ* in smFISH data (Fig. S7B). Mapping average expression of communication genes back onto the pre-malignant epithelium revealed that most cells express a single dominant module, making it possible to annotate cells by their corresponding module (Fig. 4B). Strikingly, cell-states defined solely by communication gene expression coincide with those identified by clustering the entire transcriptome (Figs. S7C–E). We further observe similar patterns in scATAC-seq data, where each subpopulation maintains open chromatin around genes of distinct communication modules, supporting an epigenetic basis for the emergence of these programs (Fig. S7F).

Of note, normal and regenerating epithelial cells (N1, N2) showed much less diversity in communication module expression compared to their *Kras*-mutant counterparts, with most cells maintaining very low module expression (Fig. 4C). Among cells with wild-type *Kras*, a small injury-induced population does express communication modules associated with the Gastric (E6) cell-state, likely reflecting the expected trans/dedifferentiation of acinar cells under inflammatory conditions (51). These cells also expressed high levels of transcripts derived from a mutant *Kras* signature (23), implying that high level RAS signaling may play a role in normal regeneration and that KRAS mutation might stabilize such transient, injury-induced communication modules.

Moreover, communication modules established in the pre-malignant pancreas are maintained in advanced cancers (K5, K6), with most cells expressing at least one of the Gastric (E6), Progenitor (E7), or Bridge (E3) modules (Fig. 4D). These modules (as well as their corresponding cell-states) are observed in an analogous mouse model of pre-neoplasia with activation of mutant *Kras* in adult acinar cells (52) (Fig. S8). Furthermore, these communication modules are conserved in human PDAC derived from multiple patients (32) (Fig. 4E). The distinct behavior of communication modules in early neoplasia across multiple model systems and their persistence in advanced murine and human PDAC implies a functional role in pancreatic tumorigenesis.

### Extensive epithelial-immune interactions drive oncogenic tissue remodeling

The striking distinction of communication modules that arise in *Kras*-mutant epithelial cells during inflammation compared to normal regeneration implicate one or more signaling nodes in early PDAC development (20–22). Tissue damage produces inflammation and changes in immune cell-state and composition that contribute to neoplasia (53); thus we investigated how mutant *Kras*-driven epithelial communication modules interact with infiltrating and tissue-resident immune cells. scRNA-seq analysis of immune cells (CD45^+^ sorted) from *Kras*-mutant tissues, before and after induction of pancreatitis (K1–K3), identified all expected immune subtypes, including both abundant (macrophage) and rare (Treg, ILC) types (Fig. S9) (27). As expected, injury-induced inflammation causes dramatic remodeling of the immune cell landscape, including the enrichment or depletion of specific lymphoid and myeloid cell-states (Fig. S10A).

Applying Calligraphy to these data identified consistent and structured communication modules defining distinct immune populations. To achieve even greater resolution, we ran Calligraphy separately on T cells/ILCs/NK cells, myeloid cells, and B cells, and found numerous modules containing known regulators as well as candidates for pancreatic tumorigenesis (Fig. S10B,C). For example, T cell/ILC/NK cell module 8 is highly expressed in ILC2, ILC3/LTi and Treg cells; these cells express the receptor for IL-33 (*Il1rl1*/*Il1rap*), a ligand that accelerates the formation of mucinous PanIN lesions (23).

Reasoning that such rapid immune and epithelial remodeling could arise through heterotypic crosstalk in pancreata undergoing neoplastic transformation, we utilized a feature in Calligraphy to nominate potential cell-cell interactions that drive this process (Fig. S11A) (27). To limit our search to tumorigenesis-specific crosstalk following tissue inflammation, we filtered Calligraphy modules to retain those cognate R-L pairs in which at least one partner is selectively upregulated in *Kras*-mutant (K2) relative to *Kras*-wild type (N2) epithelial cells (23). This filtering reduced the space of possible interacting molecules from 340 total communication genes down to 55 receptors and 46 ligands potentially involved in tumorigenesis-associated communication. We then assumed that two cell-states potentially interact if their associated modules are enriched in the number of shared cognate R-L pairs spanning them (27). Whereas CellphoneDB (50) predicts a highly dense network of 720 out of 729 (98.8%) of possible interactions, involving nearly all pairwise combinations of cell-states, Calligraphy identified a sparser, more interpretable network of potential neoplasia-specific interactions between the *Kras*-mutant epithelium and the immune environment (5.6% of possible interactions, Fig. S11B,C and Table S7).

Within Calligraphy’s context-specific network were apparent ‘master communication hubs’ that participate in numerous interactions. We calculated a receiving score (ability to sense the environment via expressed receptors) and a transmission score (ability to remodel the environment via expressed ligands) based on the number of Calligraphy’s statistically significant incoming and outgoing edges for each module (p value < 0.1) (27). The two most prominent hubs for transmitting and receiving interactions are the epithelial Gastric (E6) and Progenitor (E7) modules, respectively (Fig. 4F), which correspond to ‘high-plasticity’ populations identified above. These same communication hubs are enriched in advanced mouse and human PDAC (Fig. 4D,E).

To validate predicted communication networks, we mapped interacting modules to their spatial context, leveraging smFISH data including probes for transcripts marking distinct cell-states and their corresponding communication genes. We identified cells with concordant communication gene expression patterns across space for module E6-expressing gastric cells (*Anxa10*^+^ *Il18*^+^ *Spp1*^+^) and module E7-expressing progenitor cells (*Nes*^+^ *Il18rl*^+^ *Cd44*^+^) (Figs. 4G and S7B) in pre-neoplastic cells subject to inflammation (K2). Calculating distances between receiving (progenitor) cells and their closest sending (gastric) cells, against a random control set of gastric cells which do not express these ligands, showed a significant enrichment (t-test, p value < 0.01) of receiving cells in the vicinity of their interacting sending cells (Fig. 4H).

### Neoplastic tissue remodeling involves feedback communication loops

**Figure 5.**
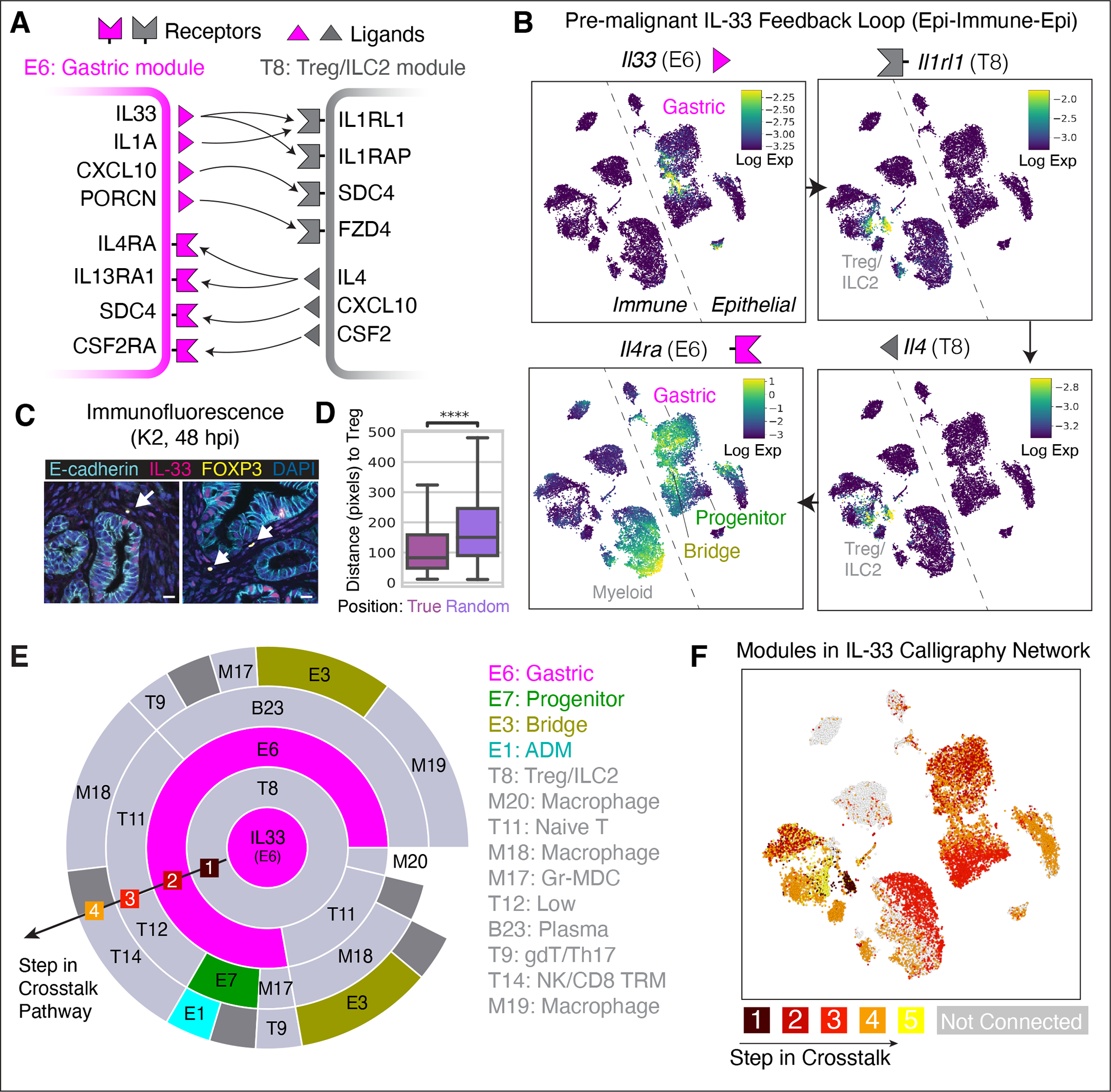
*Kras*-mutant epithelial states participate in a feedback loop with immune populations. (**A**) A feedback loop identified by Calligraphy in the pre-malignant pancreas. Arrows depict cognate R-L interactions. (**B**) tSNEs of immune and epithelial scRNA-seq data from pre-malignant stages (K1–K3, n = 6 mice), displaying imputed expression (69) of key genes from the loop in (A). Arrows between plots indicate sequential steps of the loop. (**C**) Co-immunofluorescence (co-IF) images showing co-expression of IL-33 and E-cadherin (epithelial marker), and apposition of FOXP3-expressing Tregs (arrows) and IL-33-expressing epithelial cells. Scale bar, 10 μm. (**D**) Distance in pixels (0.325 μm per pixel) of IL-33^+^ epithelial cells to Tregs against a null model of spatial distribution in co-IF data pooled across all biological replicates from K2 tissue. Distances are calculated between each IL-33^+^ epithelial (E-cadherin^+^) cell and its closest Treg (CD3^+^ FOXP3^+^). Asterisks, significant difference (one-tailed, un-paired t-test, p value < 0.0001) compared to random distances calculated by permuting epithelial cells. (**E**) IL-33-centric crosstalk paths originating from epithelial Gastric module E6 (central circle, magenta), with each outward concentric circle illustrating possible communication paths from inner to outer modules based on links inferred by Calligraphy. Arc length is proportional to the number of inner-module ligands that can bind to cognate receptors in the outer module. (**F**) tSNE as in (B), colored according to the step in which communication events from the IL-33-centric path in (E) reach the module expressed by that cell. Cells are assigned to their highest-expressed module, and each module is scored by the earliest step in which it appears along any paths through the Calligraphy network emanating from E6-derived IL-33. Cells expressing modules which are not downstream of IL-33 are colored in gray.

One of our most striking observations is the dramatic remodeling of epithelial and immune compartments within 24 to 48 hpi (see Figs. S2D and S10A). This remodeling is highly reproducible and expansive, involving numerous new cell-states that are quickly adopted by most cells. The dynamics of such a rapid and robust response suggest a feedback loop, by which immune cell intermediates may amplify tumor-promoting epithelial cues (54). We probed the Calligraphy module-module interaction network to systematically enumerate cycles involving any epithelial or immune subsets and identified only one feedback loop in the system (Fig. 5A) (27). The loop includes the Gastric (E6) hub module, which is maintained in late disease, and further involves cytokines and receptors with reported roles in KRAS-driven pancreatic tumorigenesis, including IL1A, IL-33, and IL4RA (55, 56). Specifically, it engages Treg and ILC2 cells via IL-33 signaling before feeding back to epithelial cells (Fig. 5B).

*Il33* is expressed during pancreatitis by a small subset (4%) of TFF1/ANXA10^+^ *Kras*-mutant Gastric module-expressing epithelial cells and is predicted to initiate signaling to Tregs and ILCs (Module T8) by binding with its cognate receptor *Il1rl1* and co-receptor *Il1rap* (Figs. 5B and S12A–D). Supporting the relevance of these interactions, immunofluorescence (IF) data reveal that IL-33-expressing epithelial cells (IL-33^+^ mKate2^+^) and rare Tregs (Foxp3^+^) are in close spatial proximity in *Kras*-mutant pancreata under injury conditions (distance vs. randomly permuted positions, t-test p value < 0.01 for all IF images collected across five independent mice) (Figs. 5C,D and S12E). Subsequently, many receiving cells that express *Il1rl1* (Module T8) also express the Th2 cytokine gene *Il4* (Fisher’s exact test; odds ratio = 21.47, p value = 9.88 × 10^−35^), consistent with the known role of IL-33 in triggering Th2-type immune responses (57). Module T8 cells then apparently signal through IL-4 (*Il4)* back to the Gastric Module (E6) via the IL-4 receptor (*Il4ra*), thereby closing the loop and potentially propagating signals to other modules in both immune and epithelial compartments (Fig. 5B).

The broad expression of the IL-4 receptor across *Kras*-mutant epithelial cell-states, including gastric, tuft cell and *Nes*^+^ progenitor populations (Figs. 5B and S12A,C), implies that this signaling loop has a system-wide impact on pre-malignant tissue (45% of pre-malignant epithelial cells appear impacted). In contrast, few wild-type normal pancreas cells express both sending (*Il33*) and receiving (*Il4ra*) factors, and do so at low levels, even during injury-induced regeneration (Figs. S12F,G).

A particular strength of Calligraphy is its ability to dissect the complexity inherent to tissue crosstalk by constructing communication circuits linking a cascade of signaling events between multiple communication modules in a serial fashion, thus mapping cell populations that are potentially both directly and indirectly affected by epithelial-derived IL-33. Calligraphy predicts that IL-33-driven communication has a large impact on pre-malignant tissue, and the proportion of affected tissue increases via signaling cascades between communication modules that each utilize multiple cognate R-L pairs, ultimately reaching the vast majority of the pre-malignant pancreas (72% of cells, Fig. 5E,F). While it is unlikely that the IL-33 loop is solely responsible for KRAS-driven tumor progression in the context of the high complexity of observed intercellular communication in the pre-malignant tissue, the number of populations that appear directly and indirectly impacted by epithelial IL-33 expression suggests that this communication circuit plays an important role in driving tumorigenesis.

### KRAS-dependent IL-33 feedback loop directs rapid tissue remodeling in early tumorigenesis

**Figure 6.**
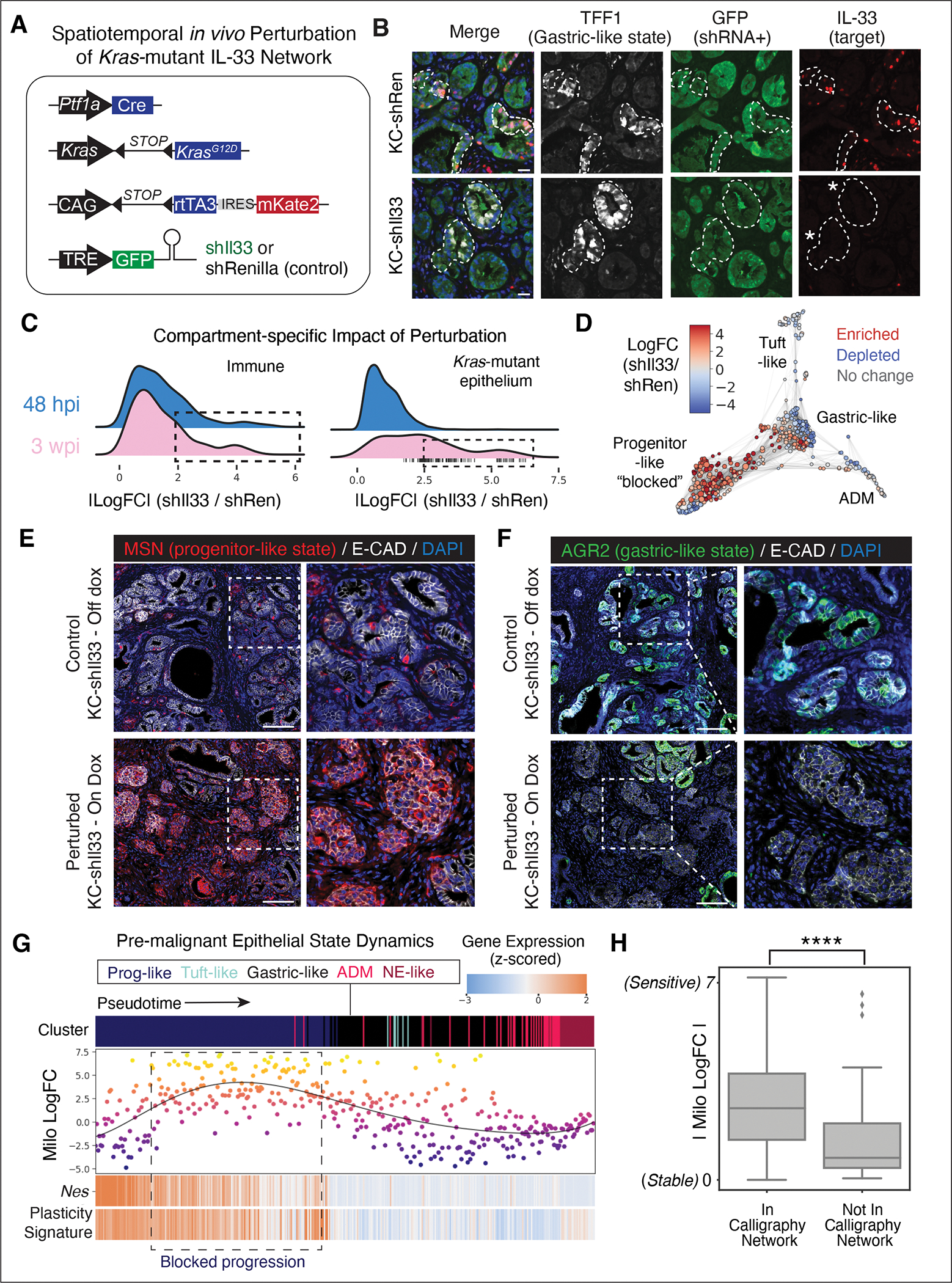
Spatiotemporal in vivo perturbation of *Il33* impairs neoplastic progression. (**A**) Mouse models for inducible repression of *Il33* (KC-sh*Il33*, 2 independent strains) or Renilla control (KC-shRen), restricted to *Kras*-mutant epithelial cells by *Ptf1a*-Cre expression. (**B**) Representative IF of pancreata from control (top) or KC-sh*Il33* (bottom) mice placed on dox at 5 weeks of age and analyzed 9 days later at the 48 hpi timepoint (K2). *Kras*-mutant epithelial cells, not surrounding stroma, express *Il33* shRNA marked by GFP in KC-sh*Il33*; TFF1 marks cell-state in which IL-33 is activated at 48 hpi in control but not sh*Il33* animals. Dashed lines demark epithelium-stroma boundary, asterisks highlight suppression of *Il33* in TFF1^+^ metaplastic cells of KC-sh*Il33* mice. DAPI marks nuclei (blue). Scale bar, 20 μm. (**C**) Milo (60) log fold change (logFC) magnitudes across cell neighborhoods (n = 5 mice), with higher values indicating greater impact of IL-33 perturbation. Rightward distribution shifts (dotted lines) indicate a larger impact on particular cell-states; vertical dashed lines indicate neighborhoods with significant (adjusted p < 0.1) shifts according to Milo, appearing only in K3 epithelia. (**D**) FDL of Milo neighborhoods colored by logFC of abundance in sh*Il33* samples relative to controls, at the late (3 wpi) timepoint. (**E,F**) Representative IF in pancreata from KC-sh*Il33* mice placed on-dox (bottom) or off-dox (top) at 3 wpi (K3), showing (**E**) aberrant accumulation of progenitor-like state (MSN^+^) in epithelial cells (E-cadherin^+^) of IL-33-perturbed animals at 48 hpi and (**F**) depletion of gastric-like (AGR2^+^) states upon epithelial IL-33 suppression. DAPI marks nuclei (blue). Scale bar, 100 μm. (**G**) Impacts of *Il33* perturbation across *Kras*-mutant epithelial neighborhoods at K3 (3 wpi). Top, pseudotime-ordered neighborhoods (columns) colored by cell-state. Middle, neighborhoods plotted and colored by Milo logFC; higher logFC denotes greater abundance in sh*Il33* relative to control. Bottom, *Nes* and plasticity-associated gene expression (Fig. 3F) (27); heatmap colors scaled to ±2 s.d. from mean. (**H**) Milo logFC of neighborhoods mapped to modules that are (left) or are not (right) downstream of Calligraphy’s IL-33-centric network; asterisks, indicate significance (unpaired, one-tailed t-test, p value = 1.24 X 10^−7^).

Whereas previous studies have implicated stroma-derived IL-33 in disease phenotypes (58, 59), Calligraphy identified a feedback loop driven by IL-33 expressed from epithelial cell-states. To determine how IL-33 derived specifically from the epithelium contributes to early neoplasia, we developed a GEMM that enables specific *Il33* suppression in lineage-traced *Kras*-mutant epithelial cells. Animals were produced from multi-allelic embryonic stem cells engineered to harbor a conditional *Kras*^G12D^ allele together with a doxycycline (dox)-inducible GFP-coupled short hairpin RNA (shRNA) capable of suppressing *Il33* (KC-sh*Il33*), allowing potent *Il33* suppression in the epithelial compartment following dox administration (Fig. 6A,B). Additionally, a separate cohort of animals was produced harboring a control shRNA (shRen) to control for potential perturbations of the RNA interference machinery and dox. scRNA-seq and spatial imaging (Imaging Mass Cytometry, IMC) were performed on each model assessing epithelial and immune compartments at an early time-point (48 hpi), when inflammation unleashes neoplastic remodeling (K2), and later (3 weeks post-injury, or wpi), when PanIN lesions normally emerge (K3). As expected, IL-33 expression remains intact in non-epithelial pancreatic cells (*Il33*^+^ *Vim*^+^ or *Il33*^+^ *aSMA*^+^) (Fig. S13A–D) in shIl33 animals on dox, and is specifically abrogated in *Kras* mutant cells expressing the gastric markers TFF1 and ANXA10 (Figs. 6B and S13A,B).

Analysis of co-embedded scRNA-seq data derived from control or KC-sh*Il33* on-dox mice show that IL-33 perturbation profoundly shifted the observed cell-states within both epithelial and immune compartments. We applied the Milo algorithm (60), which characterizes such local shifts (as opposed to loss or gain of entire clusters) by grouping similar cells into ‘neighborhoods’ and identifying those neighborhoods which are differentially abundant between perturbed and control conditions. Consistent with an epithelial-to-immune crosstalk, we found that *Il33* suppression in the *Kras*-mutant epithelium results in rapid remodeling of the immune landscape, with multiple immune subpopulations shifting in abundance by 48 hpi (Fig. 6C). Epithelial remodeling is delayed by comparison; a lack of substantial changes at the early time point is followed by dramatic remodeling of many epithelial cell-states at 3 wpi (K3). By this time, the perturbation of IL-33-mediated crosstalk generates evidence of neoplastic epithelial remodeling, with a shift to more cells in progenitor-like and fewer in gastric-like states (Figs. 6D and S13E,F), also seen by immunofluorescence data (Fig. 6E,F).

To gain insights into the impact of IL-33 on the dynamics of cell-state transitions, we used Palantir (61) to infer a pseudotime ordering of epithelial neighborhoods at 3 wpi, beginning from the *Nes*^+^ progenitor state (Fig. S13E). Ordering Milo log fold-change values along this pseudotime axis confirms that more IL-33-perturbed epithelial cells accumulate in earlier states expressing progenitor markers (*Nes*) and other genes associated with a plastic state (plasticity score correlation p value < 0.01) (Figs. 6D,G). Although *Il33* is expressed in only a small fraction of *Kras*-mutant epithelial cells, *Il33* perturbation results in marked changes in the cell-state composition of the pre-malignant pancreas, apparently by preventing the transition from a plastic progenitor-like state into distinct PanIN populations, such as the gastric-like cells that are normally abundant in unperturbed epithelia by 3 wpi.

The widespread changes in cell-state due to IL-33 perturbation support Calligraphy’s prediction of the relevance of this feedback loop. To more directly link the specific predicted interacting partners with observed perturbation-induced changes, we mapped each Milo neighborhood to Calligraphy communication modules (Fig. S13G) and evaluated the extent to which cell-states predicted to be downstream of IL-33-mediated crosstalk overlap with cell-states impacted by the perturbation. Qualitatively, we found the largest impact of *Il33* perturbation on Progenitor and Bridge modules, both of which are predicted to be downstream of IL-33 and express IL-4 receptor (*Il4ra)* (see Fig. 5B,E); whereas the only two modules not downstream of IL-33 are those with the smallest effect sizes (E2 and E5). Quantitatively, cell-states predicted by Calligraphy to participate in the IL-33 network were more significantly affected by the *Il33* perturbation (one-sided t-test; t = −5.25, p value = 1.24 X 10^−7^) (Fig. 6H). These results functionally validate Calligraphy as an approach to infer both communication circuits and the specific subpopulations impacted (directly and indirectly) upon perturbation of such networks.

## Discussion

While much is known about the molecular processes affecting tumor progression to advanced PDAC, pancreatic cancer is diagnosed late, and the paucity in molecular studies of early neoplasia has left us with little knowledge of how it emerges from a relatively homogeneous epithelium. By combining single-cell sequencing of mouse models with computational analysis, we found that permissive chromatin states in *Kras*-mutant cells diversify the communication programs available to pre-neoplastic tissue, expanding downstream crosstalk throughout the tumor microenvironment. Moreover, in the *Kras*-mutant context, epigenetic reprogramming and the emergence of cancer-driving populations is remarkably dynamic, occurring within two days of insult by inflammation.

Mutation is known to drive plasticity in lung cancer via the loss of AT1 or AT2 lineage identity and acquisition of a phenotype intermediate between these states (17, 18). In the pancreas, a similar loss of acinar identity and gain of an intermediate acinar-ductal state have long been observed in both tumorigenesis and regeneration; thus, traditional notions of plasticity are insufficient to describe its contribution to disease. We defined plasticity as the potential of a cell to manifest diverse future fates, motivating a generalizable plasticity score that tracks with the degree of epigenetic priming. This score nominated several highly plastic cell-states, in which open chromatin unlocks access to multiple distinct gene programs observed in benign lesions or malignant disease and revealed that inflammation enhances plasticity across these states.

To better elucidate the emergence of plastic states, we sought to reconcile prior work proposing different cells-of-origin for neoplasia. Our GEMMs harbor mutant *Kras* in all acinar cells, allowing us to comprehensively explore which states can initiate tumorigenesis. Using CellRank (39), we traced the origins of epithelial transcriptional diversity to multiple ‘apex’ progenitor populations that correspond with experimentally determined cells-of-origin. These populations also exhibit high plasticity scores and unify prior work by suggesting that neoplasia can arise from multiple *Kras*-mutant cell-states through distinct responses to inflammation. Moreover, our *Ptf1a-*Cre model traces this diversity back to a predominantly acinar-like state, supported by the fact that nearly all pre-malignant epithelial cell-states have an acinar-like chromatin state (epigenetic ‘memory’), which itself maps to a CellRank-predicted apex state. While non-acinar lineage cells can also undergo neoplastic transformation in mice (21, 62), our results agree with the observed loss of normal lineage identity upon *Kras* mutation and inflammation (63) and reveal apex states which may emerge following this transition. Among apex states, multiple unbiased analyses in particular support a *Nes*^+^ progenitor-like state (44, 45), which displays PDAC-associated chromatin alterations, expresses progenitor-associated genes, and scores highest for our plasticity metric—all hallmarks of highly plastic cells.

Our plasticity score was most correlated with cytokine and receptor genes, implying that plastic populations are primed to both signal and respond to the environment. Addressing this, we asked how cell-cell communication may drive rapid tissue remodeling. Communication inference approaches failed to find specific signals among the large number of cytokines and receptors expressed across cell populations. We therefore developed Calligraphy to leverage modularity in gene expression for greater power and robustness over testing individual receptor-ligand gene pairs, allowing us to focus on neoplasia-specific communication networks.

Calligraphy identified modules of co-expressed communication genes that, surprisingly, mapped one-to-one to transcriptional cell-states, implying that communication is critical for establishing cell-state diversity within the pre-malignant pancreas. These networks were largely absent from normal pancreas, with only one being induced in a rare subpopulation of cells that emerges upon tissue damage. The same module has the highest propensity for tissue remodeling and persists in advanced murine and human cancers, demonstrating that cancer commandeers gene programs used during normal regeneration.

Our analyses revealed a feedback loop initiated by IL-33 signaling from epithelial cells expressing the Gastric module to Th2 cytokine-expressing Tregs and ILCs, which signal back to the epithelium (among multiple other routes). These findings link previous results on the relevance of Th2 signaling in PDAC tumorigenesis (56) to those on the role IL-33 in this process (23). Spatial analysis revealed co-localization of signaling populations in the loop, and epithelial *Il33* knockdown in a GEMM impaired inflammation-driven remodeling of plastic populations, blocking the emergence of gastric-like state cells that are otherwise abundant in PanIN lesions. This mechanism can be driven solely by epithelium-derived IL-33, despite the high stromal IL-33 expression previously implicated in disease phenotypes (58, 59). Further, the results of in vivo *Il33* perturbation support Calligraphy inference, by matching predictions of which populations are perturbed, to what degree, and in what temporal sequence. Other modules defined herein are likely also to have functional importance. Future work can extend this approach to other niche components such as fibroblasts or endothelial cells and should expose additional communication with potential for therapeutic or diagnostic exploitation.

PDAC is frequently detected too late for curative intervention, a detailed understanding of early neoplastic events may enable the development of rational strategies to prevent, detect, and intercept tumors before they progress to an intractable stage. Our results show that GEMMs can be used to study and perturb early events, revealing epigenetically plastic cell-states in neoplasia that are not observed in the normal or regenerative pancreas. Further efforts to understand neoplasia-specific communication networks driving PDAC initiation hold promise for the development of therapeutics that block early cancer progression, and may also be effective against advanced disease.

## Materials and methods summary

### Experimental design:

Samples from GEMMs were collected to span the entire range of PDAC progression (K1-K6) as well as regenerating pancreata (N1-N2). Additional samples were collected from GEMMs enabling selective genetic perturbation of pre-malignant *Kras*-mutant cells. Table S8 summarizes experimental conditions (27). All animal experiments were performed in accordance with the Institutional Animal Care and Use Committee (IACUC)-approved protocol (11–06–018).

### Generation of bulk and single-cell omics data:

Tissue dissociation and cell preparations for bulk and single-cell ATAC-seq were performed as previously described (23). For scRNA-seq analysis, FACS-sorted epithelial or immune cells were encapsulated and processed following 10x Genomics user manual (Reagent Kit 3’ v2) as described (27).

### Spatial and immunophenotyping data:

Tissues were processed and stained for imaging (IF/IHC, IMC, H&E, and smFISH) or FACS analyses (27). Tables S9 and S10 summarize panels used for multiplexed IMC and smFISH. IMC data was collected using Hyperion Imaging System and CyTOF Software v7.0.8493.0 (Fluidigm). smFISH imaging was performed on a Nikon Ti2 inverted microscope. FACS data was acquired using a 5-laser BD LSRFortessa and analyzed using FlowJo v10.0.

### Computational analysis:

scRNA-seq data were processed with SEQC (64), filtered with a custom pipeline (27), and log library size normalized. scATAC-seq data were processed with ArchR (65). Processed transcriptomic and epigenomic datasets were analyzed with custom Python scripts for visualization, cell-state annotation, metacell inference, multimodal integration, plasticity scoring, and Calligraphy communication inference, among other analyses fully described in (27). smFISH image analysis was performed on maximum projection images with segmentation on the DAPI channel using Mesmer (66) and Python code for phenotyping and spatial analyses.

## Supplementary Material

TableS1

TableS3

TableS4

TableS5

TableS6

TableS10

TableS14

TableS18

TableS7

Supp Text

## Data Availability

All sequencing data have been deposited at the Gene Expression Omnibus (GEO) under accession GSE207943. An interactive data browser to plot gene expression trends on tSNE or FDL visualizations of scRNA-seq data is accessible at http://pdac-progression-browser.us-east-1.elasticbeanstalk.com. Code for data analysis is available at https://github.com/dpeerlab/pdac-progression (DOI: 10.5281/zenodo.7738450). KC-shIL33 ESCs for the production of EPO-GEMMs are available from the corresponding author (S.W.L.) upon request.
