# Supplementary material for: Epigenetic plasticity cooperates with cell-cell interactions to direct pancreatic tumorigenesis
Source: Science. Author manuscript; Available in PMC 2023 Jul 3. (PMC10316746; doi:10.1126/science.add5327)
Supplement: Supp Text [file NIHMS1900743-supplement-Supp_Text.pdf]

Materials and Methods

Mouse models

All animal experiments in this study were performed in accordance with protocols approved by the Memorial Sloan Kettering Institutional Animal Care and Use Committee (approval number: 11-06-018). Mice were maintained under specific pathogen-free conditions, and food and water were provided ad libitum. In all experiments with PDAC models, tumors did not exceed a maximum volume corresponding to 10% of the mouse's body weight (typically 12 mm diameter). Mice were evaluated daily for signs of distress or end-point criteria. Specifically, mice were immediately euthanized if they presented signs of cachexia, weight loss of more than 20% of initial weight or breathing difficulties, or if they developed tumors of 12 mm in diameter. No tumors exceeded this limit.

Generation and authentication of KC-shIl33 embryonic stem cell clones*:* KC-sh*Il33* mouse embryonic stem (mES) cells were generated by targeting established KC embryonic stem (ES) cells (*Ptf1a-cre*;*LSL-Kras*^{G12D}*;RIK;CHC* (*70*)*)* with two independent GFP-linked *Il33* shRNAs (shIl33.668 and shIl33.327) cloned into miRE-based targeting constructs (*71*), as previously described (*70*, *72*). Targeted ES cells were selected and functionally tested for single integration of the GFP-linked shRNA element into the *CHC* locus as previously described (*72*). Before injection, ES cells were cultured briefly for expansion in KOSR+2i medium (*73*). Two clones (KC- shIl33.668 clone 3 and KC-shIl33.327 clone 2) were used for cohort generation, single-cell omics and phenotypic analyses. The identity and genotype of the ES cells, resulting chimaeric mice and their progeny were authenticated by genomic PCR using a common Col1a1 primer (5′-CACCCTGAAAACTTTGCCCC-3′) paired with a transgene specific primer: shRen.713: 5′-GTATAGATAAGCATTATAATTCCTA-3′; shIl33.668: 5′-TTCAAATGAAACAAAGTCC-3′;

shIl33.327: 5′-TTAAAAGTGAAGTTCCTTGGA-3′, yielding a product of around ~250 bp. ES cells were confirmed to be negative for mycoplasma and other microorganisms before injection.

Mouse alleles*:* All alleles have been previously described. *Ptf1a-cre* (*27*), *LSL-Kras^{G12D}* (*74*), *p53^{fl}* (*30*, *75*)*, CHC* (*76*), and *LSL-rtTA3-IRES-mKate2 (RIK)* (*77*) strains were interbred and maintained on mixed Bl6/129J backgrounds. Combinations of these alleles enable selective isolation of epithelial cells from pancreatic tissues. Specifically, we used the pancreas-specific Cre driver *Ptf1a-cre* and the lineage-tracing allele *RIK* that, by themselves or in combination with a Cre-activatable *Kras^{G12D}* allele, enable tagging of pancreatic epithelial cells that contain wild-type or mutant *Kras* by the fluorescent reporter mKate2. An additional *p53* floxed (*p53$^{fl/+}$*) or mutant (*p53^{R172H}*) allele accelerates the transition to malignancy (*30*, *75*). **Table S11** summarizes the nomenclature used for multiallelic strains used in this work.

Cohort generation: C, KC and KPC mice were generated by strain intercrossing. To generate KC-shRen and KC-sh*Il33* mice, chimaeric cohorts of male mice derived from the ES cells described above were generated by the Center for Pancreatic Cancer Research (CPCR) at Memorial Sloan Kettering Cancer Center (MSKCC) or the Rodent Genetic Engineering Core at New York University as previously described (*70*). Only mice with a coat color chimaerism of over 95% were included for experiments.

Acute pancreatitis*:* To compare the effects of tissue injury in the transcriptional and chromatin accessibility landscapes of mutant-*Kras*-and wild-type-*Kras*-expressing pancreatic epithelial cells, C, KC, KC-sh*Ren* or KC-sh*Il33* 5-week-old male mice were treated with eight-hourly intraperitoneal injections of 80 µg per kg of caerulein (Bachem) or PBS for two consecutive

days. Mice from the same genotype and age groups were randomly assigned to PBS and caerulein sex-matched groups.

Epithelial-specific *Il33* perturbation*:* For induction of shRNA expression, KC-sh*Ren* or KC-sh*Il33* mice were switched to a doxycycline diet (625 mg per kg, Harlan Teklad) that was changed twice weekly at 4 weeks of age to induce shRNA expression, and were subsequently treated with caerulein (acute pancreatitis protocol) 6 days thereafter to study contribution to cell-cell networks and tissue phenotype during injury (pancreatitis)-accelerated neoplasia.

Sample collection timepoints: Samples were collected to span the entire range of PDAC progression, from initiation to metastasis. In malignant tissue states (K5, K6), PDAC cells were isolated from primary or metastatic cancer lesions arising in autochthonous transgenic models (KPC) that were macro-dissected away from pre-malignant or normal tissue. **Table S8** summarizes the conditions and timepoints of sample collection.

Immunofluorescence, immunohistochemical and H&E staining

Data collection: Tissues were fixed overnight in 10% neutral buffered formalin (Richard-Allan Scientific), embedded in paraffin, cut into 5 μm sections, and immunofluorescence (IF), immunohistochemical (IHC) or H&E stainings were performed following standard protocols, as previously described (*23*). The following antibodies were used: mKate2 (Evrogen Cat# AB233, RRID:AB_2571743, 1:1000), CD45 (Abcam Cat# ab25386, RRID:AB_470499, 1:100), TFF1 (Cedarlane/OriGene Technologies Cat# TA322883, 1:100), GFP (Abcam Cat# ab13970, RRID:AB_300798, 1:1000), IL-33 (R and D Systems Cat# AF3626, RRID:AB_884269, 1:150),

FoxP3 (Thermo Fisher Scientific Cat# 14-5773-82, RRID:AB_467576, 1:100), MSN (Proteintech Cat# 26053-1-AP, RRID:AB_2880353, 1:100), E-cadherin (BD Biosciences Cat# 610181, RRID:AB_397580, 1:500), AGR2 (Novus Biologicals Cat# NBP2-27393, 1:200). CD45 IHC was performed on a Bond Rx autostainer (Leica Biosystems) with Histowiz. H&E and IHC slide scanning was performed with Histowiz. IF images were acquired on a Zeiss AxioImager microscope using a 10× (Zeiss NA 0.3) or 20× (Zeiss NA 0.17) objective, an ORCA/ER CCD camera (Hamamatsu Photonics), and Axiovision or Zeiss (ZEN 2.3) software.

Computational image analysis: To analyze IF images (**Fig. 5C,D**), we first segmented them into individual cells. For segmentation, we sharpened the nuclear staining DAPI channel by subtracting a smoothed version of the image, blurred with Gaussian with a standard deviation of 20, followed by logging with a pseudo-count of 1, thresholding to the bottom 10th and top 99th percentiles, and scaling between 0 and 1. Mesmer (*66*), the current state-of-the-art bioimage segmentation algorithm, requires both a nuclear and membrane marker. As we lack an explicit membrane marker, we sought to forge a pseudo-channel from the reciprocal of the processed DAPI image under the assumption that in dense tissues such as the pancreas, there would most likely be a cell membrane wherever there are no nuclei. We visually inspected that Mesmer under this configuration works well, and from the segmentation masks, measured the average raw marker intensity in each segmented cell.

As fluorescent intensities varied between samples, we carried on individual analysis for each and phenotyped cells based on deviations from average expression within each sample. Based on z-scored logged expression profiles, with a pseudo-count of 0.1, our positivity threshold for FoxP3, CD3, IL-33 and ECAD was 2.3, 0.5, 1 and 0.5 respectively. Cells which were positive for both

FoxP3 and CD3 we marked as Tregs, cells positive for ECAD and IL-33 as IL-33^{+} Epi, and cells ECAD^{+} and IL-33^{-} as IL-33^{-} Epi.

To ascertain whether Tregs were in proximity to IL-33^{+} Epi cells, we measured the distance from each Treg to its closest IL-33^{+} Epi cell. We subset only to Tregs that were no further than 32.5 μm from any epithelial cells, to remove confounding effects from cells which were too far to interact with either IL-33^{+} or IL-33^{-} Epi cells. For a comparative null distribution, we randomly sample *n* epithelia cells, where *n* is the number of IL-33^{+} Epi cells in the sample, and measured the distance of each Treg to the closest cell in the random sample, doing so 100 times, each time selecting a new random sample for increased statistical power. Using a two-sided t-test, we found that Tregs were significantly closer to IL-33^{+} Epi cells compared to IL-33^{-} Epi cells in all 7 images across 5 individual mice, with the largest p-value being $p = 1.493 \cdot 10^{-3}$. When looking at the distances measured from all samples, the p-value was $p = 1.565 \cdot 10^{-131}$.

Imaging Mass Cytometry (IMC)

Antibodies were optimized via immunofluorescence and conjugations were carried out in house and by the Single Cell and Imaging Mass Cytometry Platform at the Goodman Cancer Research Centre (McGill University), using Maxpar Conjugation Kits (Fluidigm), according to manufacturer's instructions. Deparaffinization and heat-induced epitope retrieval were performed using the Ventana Discovery Ultra auto-stainer platform (Roche Diagnostics). FFPE slides were incubated in EZ Prep solution (preformulated, Roche Diagnostics) at 70 °C to deparaffinize, followed by antigen-retrieval in standard Cell Conditioning 1 solution (CC1,

preformulated; Roche Diagnostics) at 95 °C. Slides were then washed in 1× PBS, blocked in Dako Serum-free Protein Block solution (Agilent), followed by antibody staining overnight at 4 °C as described by Fluidigm for FFPE tissues. Tissues were stained with a panel of multiplexed metal-conjugated antibodies (**Table S9** specifies the antibodies and metals shown in **Fig. S13**). IMC images were acquired at a resolution of roughly 1 µm, frequency of 200 Hz and area of 1 mm^2, with Hyperion Imaging System and CyTOF Software v7.0.8493.0. (Fluidigm).

Sliding windows were used to extract regional information from the Imaging Mass Cytometry image data. The size of the window was 30x30 pixels. The images were padded with 0 values to be exact division of the window size. The average value of a maker is used to represent the marker expression in the window (**Fig. S13C,D**).

Single Molecule Fluorescence in situ Hybridization (smFISH) collection

Probe design for multiplex single molecule FISH: We built upon published software (*78*, *79*) to design custom panels for single molecule mRNA FISH. This design strategy relies on pre-computation of all possible 30 mer sequences found in mouse cDNAs (Ensembl GRCm38.p6), augmented with coding sequences of fluorescent proteins engineered into our mouse model. We excluded pseudogenes from the potential pool of mRNAs to design probes for. We compute multiple scores for each 30 mer, including Tm, GC content, and potential for hybridization with rRNAs and tRNAs. We used the following parameters to include a 30 mer into our candidate probe-set: GC-content (43-63%), Tm (66-76C), excluding 30 mers that contain at least a 15 mer present in a rRNA or tRNA.

In addition, we computed expression-informed penalties to estimate the specificity of each candidate probe. We adapted published software (*78*, *79*) to include single-cell information into the estimation of specificities scores. We reasoned that incorporating single-cell information would decrease the chances of selecting probes with off-target binding to highly-expressed genes in rare cell populations. To do so, we considered our single-cell data from epithelial and immune compartments of the injured pancreas. Furthermore, we leveraged published single-cell profiling of pancreatic tissue in a timecourse of *Kras*-driven transformation to incorporate information about fibroblast, pericyte and endothelial gene expression (*52*). Since our spatial analysis is focused on the pre-malignant-stage of pancreatic tumorigenesis, we excluded cancer-associated samples for the purpose of computing specificity scores.

To summarize single-cell gene expression as a function of cell-state in distinct cellular compartments (epithelial, immune, fibroblast, pericyte and endothelial), we used SEACells (v0.2.0), a recently developed algorithm for the identification of compact single-cell neighborhoods, or metacells, that collectively recapitulate heterogeneity in single-cell data, including rare cell-states (*47*). For each cellular compartment, we used standard log library-size normalization and dimensionality reduction using principal component analysis (PCA) (n_pcs=100) for pre-processing. Next, we ran SEACells, using the following parameters: n_waypoint_eigs = 10 (default) and waypoint_proportion = 0.9 (default). Since cellular compartments differ in terms of dataset size and cell-state heterogeneity, we selected the number of metacells per compartment such that the median number of individual cells per metacell was comparable between cellular compartments:

- Epithelial: 300 metacells, median size = 79 individual cells

- Immune: 150 metacells, median size = 69.5 individual cells
- Fibroblasts: 100 metacells, median size = 88 individual cells
- Pericytes: 15 metacells, median size = 63 individual cells
- Endothelial: 50 metacells, median size = 85.5 individual cells

Next, we computed a summarized gene expression matrix X of dimensions n x m, where n is the number of metacells across all cellular compartments, and m is the number of genes in the dataset. In this matrix, xij is the average normalized linear counts of gene j across individual cells in metacell i. We normalized our summarized expression matrix X by dividing each row by the total counts per metacell, and scaled by an arbitrary factor of 2000. Lastly, we identified the maximum expression per gene across all metacells, and used these as inputs to compute specificity penalties during probe design. This strategy penalizes off-target binding to highly expressed genes, even when such high expression occurs in a rare subpopulation of cells.

Transcription-wide specificity was computed as published (*78*), with the exception that we assumed that all isoforms of a given gene contribute uniformly to its total expression. We operated under this assumption because our single-cell datasets lack isoform-specific expression information. To compute the specificity score, each 30 mer was represented as a collection of overlapping 17 mer sequences (sliding windows with 1 bp shift). For each 17 mer, we calculated the fraction of times such sequence came from the on-target gene (any isoform) out all the times it appeared in the transcriptome, weighing occurrences by gene expression, to penalize off-target binding to highly-expressed genes. A final score was computed for each 30 mer by averaging the specificity scores of its constituent 17 mers. This specificity score ranges from 0 (when none of

the occurrences would come from the on-target gene) to 1 (when all occurrences come from the on-target gene). Following suggested parameters from the original MERFISH publications (*78*, *79*), we considered 30 mers with a specificity score greater than 0.75 as candidates for our panels.

Pre-computed 30 mer sequences that passed the aforementioned filters were used to compile primary probes for a select list of query genes. We selected Ensembl canonical isoforms to design probes against a particular gene. We aimed to select 92 non-overlapping probes per gene. Whenever this wasn't possible due to transcript length, homology to other genes, or other sequence properties, we allowed a maximum overlap of 20 bp between probes. The use of overlapping probes was previously reported to maximize smFISH signal (*80*) due to the probabilistic nature of probe-mRNA binding. Lastly, we appended readout sequences to each probe, which serve as recognition sequences for fluorescently labeled readout probes. In the case of genes for which we were not able to generate at least 75 probes, we added two or four copies of the selected readout sequence in order to amplify the fluorescent signal coming from such probes. Sequences of probes used in this study are included in **Table S10**.

Sample preparation for multiplex single molecule FISH: Dissected pancreata were rinsed in 1X PBS at room temperature, and fixed with 4% PFA 1X PBS solution for 3-4 h at 4 °C. Tissues were then transferred to 4% PFA 30% sucrose 1X PBS, and incubated overnight at 4 °C (*81*). For long-term preservation, fixed tissues were rinsed in 1X PBS, gently dried with a Kimwipe, and submerged in Tissue Plus O.C.T. Compound (Fisher Healthcare, 4585) in a cryomold (Tissue-Tek, 4557). Molds were placed on dry ice until all O.C.T. was visibly frozen, and stored at -80 °C long-term.

Coverslip preparation for smFISH staining: Coverslips for smFISH staining were prepared as previously described (*82*). Briefly, 40 mm-diameter #1.5 coverslips (Bioptechs, 0420-0323-2) were cleaned in batches by arranging them in a wafer boat (Entegris A23-0215) and immersing them in a 1:1 mix of 37% HCl and methanol at room temperature for 30 min. Coverslips were then washed 2 times with Milli-Q water, and one time with 70% ethanol, followed by gently drying with nitrogen gas. Cleaned coverslips were coated with a silane layer to allow stabilization of a polyacrylamide gel during smFISH staining, following published protocols (*82*). To do so, coverslips were submerged in 0.1% (vol/vol) triethylamine (Millipore, TX1200) and 0.2% (vol/vol) allyltrichlorosilane (Sigma, 107778) in chloroform for 30 min at room temperature. They were washed once with chloroform, once with 100% ethanol and dried up using nitrogen gas. Coverslips were stored long-term in a desiccated chamber.

Poly-lysine coating of coverslips: To prepare coverslips for staining individual samples, silinized coverslips were coated with 0.1 mg/mL Poly-D lysine (Thermo Scientific A3890401) at room temperature for 1 h in a 6 cm tissue culture plate. They were then washed 1 time with 1X PBS, and 3 times with nuclease-free water. Coverslips were lifted after each wash, using either tweezers or a needle, to ensure that both sides of the coverslips were exposed to the solution. Coverslips were left to dry for at least 2 h in a tissue culture hood before proceeding to tissue sectioning.

Tissue sectioning, fixation and permeabilization for smFISH staining: Tissue section preparation was conducted following published protocols (*81*). Tissue sections of 10 µm thickness were collected using a cryostat, and mounted into poly-D lysine coated coverslips. Coverslips and tissue sections were placed face-up on a 6 cm tissue culture dish, which was used as a vessel

format for all subsequent wash/incubation steps. Coverslips were dried for 5-10 min at 50 °C and placed on dry ice until completion of sectioning of all samples. Next, plates with coverslips were transferred to ice, and treated with 3 mL 1X PBS to melt the O.C.T., and fixed at room temperature with 4% PFA 1X PBS for 10 min. Coverslips were then washed three times with 1X PBS, and treated with ice-cold 70% ethanol and maintained at 4 °C overnight for permeabilization.

Pre-staining treatment of permeabilized tissues: After overnight incubation with 70% ethanol, coverslips were rehydrated with 1X PBS on ice for 10 min. To bleach endogenous fluorescence of lineage reporters, tissues were exposed to a bleaching solution composed of 3% hydrogen peroxide (Fisher, H325-500), 1:600 37% HCl (vol/vol) 1X PBS, and placed under a heat lamp for 1 h (*83*). They were then washed 2 times with 1X PBS, and one time with 2X SSC. Next, they were treated with pre-warmed (37 °C) digestion solution containing a final concentration of 20 μg/mL proteinase K (Sigma, 3115836001) 2X SSC solution, and incubated at 37 °C for 10 min. This step is suggested for enhancing permeabilization of probes in an optimized protocol for RNA staining in pancreatic tissue (*81*). To remove proteinase K, coverslips were washed 3 times with 2X SSC. To prepare coverslips for hybridization, they were treated with pre-hybridization solution, composed of 30% formamide (Thermo Scientific, AM9344) 2X SSC and incubated for at least 3 h at 37 °C, as previously described (*81*).

Staining with primary probes: Primary probes were diluted at a 100 nM final concentration per probe in 3H staining buffer, composed of 30% formamide, 10% dextran sulfate (Sigma Aldrich, D8906-50G), 1 mg/mL yeast tRNA (Thermo Fisher Scientific, 15401029) 2X SSC (*82*). In addition, this staining solution had a final concentration of 2 μM anchor probe, a 15 nt sequence

of alternating dT and thymidine-locked nucleic acid (dT+) with a 5′-acrydite modification (Integrated DNA Technologies), designed to anchor all poly-adenylated RNAs to a polyacrylamide gel in subsequent steps. Next, hybridization chambers were prepared by attaching parafilm on the surface of a 6 cm tissue culture dish. Upon completion of pre-hybridization incubation, a 100 μL droplet of hybridization solution + probes (100 nM per probe) was placed on the center of the hybridization chamber, and coverslips were placed face down so that the hybridization solution uniformly covered the tissue, taking care of removing bubbles that may have formed in the parafilm-coverslip interface. Hybridization chambers were placed on a 15 cm dish, with a wet Kimwipe used as a humidity buffer, and incubated at 37 °C for 36 h-48 h.

Post-hybridization wash: Upon completion of incubation with primary staining solution, post-hybridization wash buffer composed of 30% formamide 2X SSC was prepared, and pre-heated to 37 °C. Coverslips were then washed face-up with post-hybridization wash buffer at 47 °C for 30 min. This washing step was repeated for a second 30 min incubation with fresh post-hybridization wash buffer. Lastly, coverslips were transferred to 2X SSC solution and maintained at 4 °C until the next step.

Gel embedding: Samples were embedded on a thin layer of polyacrylamide gel, to allow subsequent tissue-clearing through digestion of protein and lipids. To prepare the workspace for gel embedding, microscope glass slides (Premier, 6101) were washed with 70% ethanol and RNAse away (Fisher Scientific, 21-402-178), placed on top of a lab bench, and covered with 0.5 mL gel slick (Lonza, 50640), the excess of which was cleaned with a Kimwipe. The gel solution was composed of 4% (vol/vol) of 19:1 acrylamide/bis-acrylamide (BioRad, 1610144), 60 mM Tris·HCl pH 8 (Invitrogen, 15568-025), 0.3 M NaCl (Boston Bioproducts, R-244), supplemented

with the polymerizing agents ammonium persulfate (Sigma, 09913) and TEMED (Sigma, T7024) at final concentrations of 0.03% (wt/vol) and 0.15% (vol/vol), respectively, as previously described (*82*). The solution was then degassed using a vacuum chamber (Thermo Scientific, 53050609) until bubbles stopped rising to the surface of the solution. Coverslips were rinsed two times with gel solution. A 100 µL droplet of gel solution was placed on a glass slide, and coverslips were placed face-down on the slide so that the gel solution spread evenly at the slide-coverslip interface. Polymerization completed in the course 2 h at room temperature, after which gel-embedded coverslips were lifted from the glass slide with the aid of a razor-blade, and transferred to a 6 cm tissue culture dish with 2X SSC.

Digestion: Gel embedded samples were subjected to an overnight treatment with digestion solution, aimed at clearing proteins and lipids from the samples, improving the signal to noise for RNA detection. Digestion solution was composed of 2% SDS (Invitrogen, AM9822), 0.25 % TritonX (Acros organics, 327371000), 1:100 dilution of proteinase K (NEB, P8107S) 2X SSC. Samples were incubated overnight in digestion solution at 37 °C. Following overnight digestion, samples were rinsed one time with 2X SSC, transferred into a separate plate with 2X SSC, and washed for 30 min with gentle agitation. The 2X SSC solution was replaced, for a second 30 min wash.

Staining with secondary probes: We used readout probes constituted by a 20 bp oligonucleotide conjugated to a fluorophore (Alexa Fluor 488, Cy3B, Cy5 or Alexa Fluor 750) via a disulfide bond. Fluorescent conjugated probes were purchased from Biosynthesis Inc. The secondary staining solution was composed of 5% ethylene carbonate (Sigma Aldrich, E26258-100G) 2X SSC. The secondary staining solution was supplemented by a secondary readout probe for each fluorescent

color at a 3 nM final concentration, and with DAPI at a 1 μM final concentration. Secondary staining was conducted following the same procedure for the primary staining step with the exception that it was conducted for 20 min at room temperature, covering samples with aluminum foil. Following hybridization, samples were washed once with a 10% ethylene carbonate 2X SSC solution for 20 min with gentle agitation, and three times with 2X SSC for 5 min per wash.

Iterative smFISH imaging: We prepared the following buffers for iterative smFISH imaging: (1) Wash buffer: 10% ethylene carbonate 2X SSC, 2.5 mL per staining round; (2) Cleavage buffer: 10% TCEP (Sigma-Aldrich, 646547-10X1ML) 2X SSC, 3 mL per cleavage round. TCEP in the cleavage buffer allows reduction of disulfide bond linking fluorophores to oligonucleotides in readout probes for rapid extinction of fluorescent signal; (3) Imaging buffer: 10% glucose 2X SSC, supplemented with catalase (Sigma-Aldrich C3515, 17.5 μg/mL final concentration) and glucose oxidase (Sigma-Aldrich, G2133; 1.4 mg/mL final concentration), 2 mL per imaging round. Imaging buffer was stored under a layer of 1.5 mL mineral oil to minimize oxygen in solution during sequential rounds of staining and imaging; (4) 2X SSC, 40-50 mL per experiment. Furthermore, we prepared readout probe mixes for each round of staining. Readout probes were diluted to a final 3 nM concentration per probe, in 5% ethylene carbonate 2X SSC, supplemented with Murine RNAse inhibitor (NEB, M0314S; 1:400 dilution). Combinations of readout sequences and target mRNA species are defined in **Table S10**. Buffers and readout probe mixes were loaded into a custom-build fluidics control system (*84*) that can interface with the NIS Elements image acquisition software (v 5.31.02) using custom macros.

Coverslips were mounted in a commercial flow chamber (Bioptechs, FCS2) sandwitched between a 0.75 mm-thick flow chamber gaskets (Bioptechs, 1907-100; DIE, F18524), a micro-aqueduct slide (Bioptechs, 130119-5NC) and a second 0.75 mm-thick flow chamber gaskets

(Bioptechs, 1907-100; DIE, 449673-A), as previously described (*78*). To mount samples into the flow chamber, we first cut the gel so that it would fit in its entirety within the rectangular opening of the flow chamber gasket. We placed the flow chamber for imaging on a Nikon Ti2 inverted microscope using the FCS2 stage adapter (Bioptechs, 060319-2-2611), and used our fluidics system to flow in 20X SSC into the sample in order to eliminate bubbles in the tubbing and chamber. Next, we flowed imaging buffer into the sample and generated a low magnification map of the entire tissue using a 20X Plan APO objective (Nikon, MRD00205). We then switch objectives to a high magnification 60X Plan APO immersion oil objective (N.A. 1.4, W.D. 0.13 mm, FOV 25 mm; Nikon, MRD01605) required to resolve individual mRNAs. We used tape to minimize the movement of the plate-holder during sequential rounds of imaging, which we found to be important to prevent positional drift throughout the experiment.

Imaging cycles were conducted using the following parameters:

- Staining. Flow staining buffer for 4 min at a rate of 0.5 mL/min. Incubate for 20 min.
- Wash. Flow wash buffer for 5 min at a rate of 0.4 mL/min.
- Imaging. Flow imaging buffer for 3 min 40 seconds at a rate of 0.5 mL/min. Take 7 z-stacks per field of view, using a 1 µm step size, for a coverage of -3 µm to 3 µm around the mid-plane, using perfect focus throughout the entire experiment.
- Cleavage. Flow cleavage buffer for 4 min at a rate of 0.5 mL/min. Flow cleavage buffer for 10 min at a rate of 0.1 mL per min. Incubate for 10 min. Flow 2 X SCC for 5 min at a rate of 0.5 mL per minute.

To verify that each cleavage round effectively eliminated fluorescent signals, and to allow identification and computational subtraction of autofluorescence from non-cleavable sources, we took images after each staining and cleavage round. In addition, we collected images from FOVs without tissue or sources of bright autofluorescence that would allow us to estimate non-uniform illumination and detection profiles in each fluorescent channel, and correct for these in downstream image processing steps.

smFISH image processing and analysis

Initial processing: To collapse z-stacks into a single 2D image, maximum projection images were generated using the Nikon Elements software's maximum projection function. After each round of FISH imaging, we took an additional image with cleaved fluorophores to capture the background signal for each channel. As the microscope has unequal sensitivity to the 5 different fluorophores, we also imaged each fluorophore's flat field to capture its bias. We corrected raw FISH images by subtracting the background signal of each gene and then dividing by the flatfield bias of the conjugated fluorophore, then thresholding to 0 to correct any negative-valued pixels.

We used the DAPI image from the first round of staining to perform nuclear segmentation on our corrected iterative FISH data. For accurate segmentation, we first sharpened the DAPI staining by applying an inverse blur filter, subtracting the original image blurred with a Gaussian with standard deviation of 20 pixels from the original images, multiplying by a factor of 2, and adding that to the original images. We rectified any pixels with values below zero, log-transformed the image with a pseudocount of 1 to sharpen, thresholded the log-transformed image to between the

20th and 95th percentile, and scaled resulting pixel values to between 0 and 1. We applied Mesmer (*66*) for segmentation with the approach described above in "Immunofluorescence, immunohistochemical and H&E staining."

Segmented cell phenotyping: To analyze individual cells in our smFISH data, we summarized gene expression per cell by averaging the signal for each gene over all pixels within each segmentation boundary. The resulting cell-by-gene expression matrix was subjected to PCA for dimensionality reduction. The first PC correlated strongly with the total signal per cell (sum of signal across all genes), an artifact which may relate to technical distortions across or within single FOVs. We thus performed all downstream analysis on the first 50 PCs excluding PC1 to limit the impact of this technical bias. We also excluded 6 out of 47 FOVs which appeared as outliers in embeddings even with PC1 exclusion. Known co-expressed markers (*Msn*, *Nes*, and *Cd44*, all of which mark progenitor-like cells) were much more coherent in 2D embeddings compared to embeddings that include PC1, which would otherwise be heavily skewed by total signal per cell. In particular, by excluding PC1, RIK allele expression marking epithelial cells became clearly separated from non-epithelial (stromal and immune) populations in 2D space. This enabled coarse phenotyping of each population by first selecting epithelial cells with high RIK expression (signal > 0.5, determined by inspection of the total RIK distribution and thresholding for the upper mode).

We re-computed PCA on epithelial cells, discarding PCs with high total signal correlation as above. In a 2D embedding built on these PCs, we noticed a continuum spanning progenitor-like (*Msn*$^{+}$, *Nes*$^{+}$) and gastric-like (*Anxa10*$^{+}$, *Gkn1*$^{+}$) populations, with many cells apparently intermediate for markers of both or lacking expression of either. To annotate cells, we thus

inspected the relative expression of these marker sets across cells to determine four categories: progenitor-like, gastric-like, transitional (spanning progenitor and gastric phenotypes), or neither. This was accomplished by first clustering epithelial cells with PhenoGraph (k = 30) (*85*) based on log-expression (pseudocount = 0.1) of these 4 markers. This clustering was found to more faithfully capture the distinction between states than clustering based on all markers imaged, which contributed noise in cases where markers were not expressed highly or robustly. As such, inspection of per-cluster marker expression allowed annotation of the four aforementioned classes, visualized in **Fig. S3E**. Gastric and progenitor annotations were used for downstream spatial proximity analyses described below.

Spatial Proximity Analysis: We sought to explore the spatial distribution of several cell-states identified using single-cell RNA-seq data, including those identified with the Calligraphy algorithm (see "Heterotypic cell-cell communication analysis with Calligraphy" below) as participating in specific receptor-ligand-driven communication events. In particular, we set out to determine whether computationally predicted "sending" gastric populations (expressing ligands *Spp1*, *Il18*) and the "recipient" progenitor cells (expressing receptors *Cd44*, *Il18r1*) are in close spatial proximity based on smFISH data (see **Fig. 4F**). To this end, we leveraged gastric and progenitor cell annotations above along with expression of receptor and ligand genes to identify sending and receiving cells. We began by ensuring that gastric and progenitor marker genes used for phenotyping (see "Segmented cell phenotyping" above) are indeed expressed in the same cells as the receptors and ligands of interest (**Fig. S7B**). Then, within each gastric or progenitor category, we identified cells with high levels of expression of both ligands or both receptors respectively. Specifically, each gastric cell expressing both ligands above their 25th percentile was considered a sending cell, and each progenitor cell expressing both receptors above their

25th percentile is likewise considered a receiving cell. This approach identified 1,503 sending cells and 1,989 receiving cells out of 8,789 total epithelial cells, several of which are visualized in close spatial proximity in **Fig. 4G**.

To test the significance of spatial proximity between pairs of cell-states, we first computed the distance between each receiving progenitor cell (double positive for both receptors) and its closest sending gastric cell (double positive for both ligands). As a negative control, we computed the distance between the receiving cell and 100 randomly selected non-sending gastric cells (not positive for both ligands) within the same FOV. If fewer non-sending (control) cells than sending cells existed in a particular FOV, we discarded that receiving cell from the analysis, so as to not bias the results based on regions with low heterogeneity. We then applied an un-paired t-test to determine the statistical significance of the difference between distances spanning receiving cells and sending versus non-sending cells (**Fig. 4H**).

Tissue dissociation and single cell analyses (scRNA-seq, scATAC-seq, bulk ATAC-seq)

For scRNA-seq and scATAC-seq collection, lineage-traced (mKate2$^+$) epithelial cells or CD45$^+$ immune cells were freshly isolated from pancreatic tissues from C, KC, KPC, or KC-shRNA mice by FACS sorting. Specifically, pancreases were finely chopped with scissors and incubated with digestion buffer containing 1 mg/mL collagenase V (Sigma-Aldrich, C9263), 2 U/mL Dispase (Life Technologies, 17105041) dissolved in HBSS with Mg^{2+} and Ca^{2+} (Thermo Fisher Scientific, 14025076) supplemented with 0.1 mg/mL DNase I (Sigma, DN25-100MG) and 0.1 mg/mL soybean trypsin inhibitor (STI) (Sigma, T9003), in gentleMACS C Tubes (Miltenyi

Biotec) for 42 min at 37 °C using the gentleMACS Octo Dissociator. Normal (non-fibrotic) pancreas samples were dissociated as above, except that the digestion buffer contained 1 mg/mL collagenase D (Sigma-Aldrich, 11088858001) instead of collagenase V. After enzymatic dissociation, samples were washed with PBS and further digested with a 0.05% solution of Trypsin-EDTA (15400054, Thermo Fisher Scientific) diluted in PBS for 5 min at 37 °C. Trypsin digestion was neutralized with FACS buffer (10 mM EGTA and 2% FBS in PBS) containing DNase I and STI. Samples were then washed in FACS buffer containing DNase I and STI, and filtered through a 100 μm strainer. Cell suspensions were blocked for 5 min at room temperature with rat anti-mouse CD16/CD32 with Fcblock (BD Biosciences, Clone 2.4G2) in FACS buffer containing DNase I and STI, and an APC-conjugated CD45 antibody (Clone 30-F11, Biolegend, 1:200) or APC-Cy7 CD45 antibody (Clone 30-F11, Biolegend, 1:200) was then added and incubated for 10 min at 4 °C. Cells were then washed once in FACS buffer containing DNase I and STI, filtered through a 40 μm strainer and resuspended in FACS buffer containing DNase I and STI and 300 nM DAPI as a live-cell marker. Sorts were performed on BD FACSAria I or BD FACSAria III cell sorters (Becton Dickinson) for mKate2 (co-expressing GFP for on-doxycycline shRNA mice), excluding $DAPI^{+}$ and $CD45^{+}$ cells. FACS-sorted cells were collected in 2 % FBS in PBS.

FACS-based Immunophenotyping

Collection of immune cells for immunophenotyping followed the dissociation protocol used to isolate epithelial cells, with the following differences: (i) digestion buffer did not include dispase, and (ii) trypsin digestion step was not performed, to optimally preserve surface epitopes.

For multi-parametric flow cytometry analysis, cell suspensions were stained with LIVE/DEAD fixable viability dye (Invitrogen) for 30 min at 4 °C. After this, cells were washed, incubated with Fc block (BD, 1:200) for 15 min at 4 °C, and then stained with a cocktail of conjugated antibodies for 30 min at 4 °C. After staining cells were washed and fixed (BD Cytofix) for 20 min at 4 °C, washed again, and stored for analysis. Samples were analyzed in a BD LSRFortessa with 5 lasers, where gates were set by use of fluorescence-minus-one (FMO) controls.

The following antibodies were used to quantify the fraction and identity of IL1RL1 ST2 receptor expressing cells: AF700 CD45 (clone 30-F11; BioLegend Cat# 103128, RRID:AB_493715), BUV395 CD11b (clone M1/70; BD Biosciences Cat# 563553, RRID:AB_2738276), PerCP Cy5.5 Nkp46 (clone 29A1.4; BioLegend Cat# 137609, RRID:AB_10642684), PE eFluor 610 CD3e (clone 145-2C11; Thermo Fisher Scientific Cat# 61-0031-82, RRID:AB_2574514), PE Cy7 CD8 (clone 53-6.7; BioLegend Cat# 100722, RRID:AB_31276), Strep BUV661 (BD Biosciences Cat# 612979, RRID:AB_2870251), biotin-ST2 (clone RMST2-33; Thermo Fisher Scientific Cat# 13-9333-82, RRID:AB_2572809), BV650 CD19 (clone 1D3; BD Biosciences Cat# 563235, RRID:AB_2738085), APC-Cy7 Gr1 (clone RB6-8C5; BioLegend Cat# 108424, RRID:AB_2137485), BV605 CD4 (clone RM4.5; BD Biosciences Cat# 563151, RRID:AB_2687549), APC F4/80 (clone BM8; BioLegend, 123116), BV785 CD44 (clone IM7; BioLegend Cat# 103041, RRID:AB_11218802), BV711 EpCAM (clone G8.8; BioLegend Cat# 118233, RRID:AB_2632775), PE CD11c (clone N418; BioLegend Cat# 117307, RRID:AB_313776), FITC MHCII (clone M5/114.15.2; Thermo Fisher Scientific Cat# 11-5321-82, RRID:AB_465232).

Encapsulation and sequencing of scRNA-seq samples

Cells were resuspended in 1X PBS and BSA (0.04%) and checked for viability using 0.2% (w/v) Trypan Blue staining (Countess II). All sequencing experiments were performed on samples with a minimum of 80% viable cells. Single-cell encapsulation and scRNA-seq library prep of FACS-sorted cell suspensions was performed on the Chromium instrument (10x Genomics) following the user manual (Reagent Kit 3' v2). Each sample loaded onto the cartridge contained approximately 5,000 cells at a final dilution of ~500 cells/µl. Transcriptomes of encapsulated cells were barcoded during reverse transcription and the resulting cDNA was purified with DynaBeads, followed by amplification per the user manual. Next, the PCR-amplified product was fragmented, A-tailed, purified with 1.2X SPRI beads, ligated to sequencing adapters and indexed by PCR. Indexed DNA libraries were double-size purified (0.6–0.8X) with SPRI beads and sequenced on an Illumina sequencer (R1 – 26 cycles, i7 – 8 cycles, R2 – 70 cycles or higher) to a depth of >50 million reads per sample (>13,000 reads/cell) at MSKCC's Integrated Genomics Operation Core Facility.

Encapsulation and sequencing of scATAC-seq samples

Approximately 50,000 mKate2$^+$ epithelial (mKate2$^+$;CD45$^-$;DAPI$^-$) cells were isolated from pre-malignant pancreata by FACS and subjected to scATAC-seq protocol (10X Genomics, CG000168 RevA) (*86*). In brief, FACS-sorted cells were lysed in cold lysis buffer (0.1% NP-40, 0.1% Tween 20, 0.01% digitonin, 10 mM NaCl, 3 mM $MgCl_2$ and 10 mM Tris-HCl (pH 7.4)), washed and processed according to 'Nuclei Isolation for Single-Cell ATAC Sequencing'

protocol (CG000169 RevD), according to the manufacturer's instructions. The resulting nuclei suspension was subjected to transposition reaction for 60 min at 37 °C and then encapsulated in microfluidic droplets using a 10X Chromium instrument following the manufacturer's instructions with a targeted nuclei recovery of approximately 5,000. Barcoded DNA material was cleaned and prepared for sequencing according to the Chromium Single Cell ATAC Reagent Kits User Guide (10X Genomics; CG000168 RevA). Purified libraries were assessed using a Bioanalyzer High-Sensitivity DNA Analysis kit (Agilent) and sequenced on an Illumina platform at approximately 150 million reads (R1 50 bp, R2 50 bp, i7 8 bp, i5 16 bp) per 1 sample (around 5,000 nuclei) at MSKCC's Integrated Genomics Operation Core.

Single-cell RNA-seq data processing and basic analysis

Custom cluster-based filtering pipeline: All scRNA-seq datasets were initially processed (de-multiplexed, barcode-corrected, aligned, UMI-corrected) with SEQC (*64*) using mouse genome mm10 and default parameters for the v2 3' scRNA-seq kit. After constructing a preliminary molecule count matrix from all barcodes, SEQC filters empty drops and poor quality cells using four main criteria: cell library sizes (total transcript counts), cell coverage (average reads per molecule), mitochondrial (MT) RNA content reflecting dead or dying cells, and library complexity (number of unique genes captured by library size). However, we find that scRNA-seq data from pancreas are prone to increased technical noise, including higher MT RNA expression; thus, we opted to bypass SEQC's automated filtering to ensure our analysis did not inadvertently discard relevant cells. Instead, we developed a manual, cluster-based filtering pipeline that relies on the metrics listed above plus additional inclusion criteria for calling real,

high-quality cell populations. The guiding principle of this pipeline is that low quality or dying cells tend to form distinct phenotypic clusters with poor technical characteristics, which can be removed by iterative rounds of clustering and filtering. Manual filtering also has the advantage of allowing system-specific biological knowledge to guide filtering decisions, which is especially important when rare populations may be confounded with technical features of the data (for example, smaller cells correlate with low library sizes, or specialized cell types express a restricted set of genes, resulting in low library complexity).

We first performed a liberal per-sample filtering step to remove obvious empty drops. Specifically, we retained the 15,000 barcodes with highest total transcript counts (~5,000 cells were loaded into the lane), a permissive threshold that avoids excluding real cells with lower library size. We then clustered our data with PhenoGraph (*85*) and nominated clusters potentially comprising empty drops or dying cells based on one or more SEQC metrics of coverage, MT content and library complexity. To further assess nominated low-quality clusters, we constructed per-cluster gene-gene correlation matrices and inspected the matrix block structure, which represents co-regulated gene modules. We reason that the tight regulatory programs driving biological functions are loosened in dying cells, which should be reflected in a breakdown of modularity in gene-gene co-expression matrices. To suggest potential real populations, we also looked for coherent expression of pancreas or immune marker gene programs in each population. Finally, we removed clusters that 1) had substantially poorer-than-average SEQC metrics, and/or 2) lacked co-regulated gene modules and evidence of coherently expressed gene programs. We retained any clusters for which there was uncertainty.

After filtering each sample individually, we pooled sets of samples to increase our power to detect rare populations, and repeated clustering and filtering iteratively until only high-quality cells remained. Following the iterative filtering of pooled sets, filtered count matrices were generated independently for each sample. To guide pooling and downstream analyses, samples were grouped into three pre-defined cohorts which address questions associated with (1) full tumor progression ("Progression Cohort"), (2) the impacts of *Il33* perturbation on particular stages of progression ("Perturbation Cohort"), and (3) involvement of the immune microenvironment in both of these cases ("Immune Cohort") (see **Table S12** for assigned cohorts, filtering groups and filtered count matrix statistics).

The following sections describe the cohort-specific analysis of filtered count matrices, including data integration, normalization, dimensionality reduction, and visualization:

Progression Cohort (N1–K6 epithelial cells, sorted based on mKate2^{+}): The Progression Cohort (**Table S12**) contains all epithelial datasets from normal and regenerating pancreata (N1, N2), *Kras*-mutant pre-malignant pancreata with and without injury (K1–K4), and *Kras/p53*-altered malignant tumors (K5, K6), forming the basis for our investigation of tumor progression at various scales. All filtered datasets from this cohort were combined into a single data matrix without additional data harmonization, given the high degree of similarity and overlap between our biological replicates (**Fig. S2D**), thus allowing us to preserve the original counts. Standard log library size normalization was applied to the combined data using a scaling factor of 10,000 in lieu of median library size. We removed ribosomal genes, MT genes, *Malat1*, and genes expressed in fewer than 20 cells, resulting in a count matrix containing 16,828 genes.

An additional round of filtering was performed to remove low-quality cells which do not form separate clusters and hence passed the custom filtering described above. We inspected the log-library-size distribution of each sample separately and filtered cells below a manually determined threshold for the lower mode. By performing cluster-level analysis first (allowing us to detect populations that can only be discovered upon sample aggregation), we could ensure that entire cell types were not lost in subsequent cell-level filtering of each sample by library size. Finally, we clustered cells with PhenoGraph and removed 3 remaining outlier clusters with very low library sizes, mainly consisting of acinar cells from the basal pancreas (N1) condition. The resulting dataset comprised 30,661 high-quality cells.

After removing putative empty drops and low-quality cells, we ran DoubletDetection (*87*) for each sample individually with default parameters. Given that we expected this data to contain cells residing along continuous trajectories, we were conservative in doublet filtering so as not to remove false positives arising from true intermediate cell-states. Hence, we removed only discrete PhenoGraph clusters (k = 40, computed on all samples in the combined count matrix) which contained a high fraction (>15%) of doublets, for a total of 214 cells across samples. Finally, we removed clusters that express mesenchymal markers typically associated with fibroblasts (*Acta2, Col1a1, Lum*), representing potential stromal contaminants. Four clearly stroma-like clusters were filtered, resulting in a final dataset of 28,131 high-confidence mKate2^{+} epithelial cells.

Next, highly variable gene (HVG) selection was performed in a similar fashion to (*88*). Briefly, for each gene, we computed the mean and standard deviation of normalized (unlogged) expression across all cells, then fit a Lowess regression model to the trend of log(coefficients of

variation, pseudocount = 0.1) vs. log(mean expression). HVGs were identified by coefficients of variation that substantially exceed mean expression according to this trend, generating high residuals in the regression. To avoid biasing toward highly expressed genes, we binned genes by mean expression (40 total bins of expressed genes) and selected the top 200 with high residuals in each bin, resulting in a total of 8,000 genes for downstream analysis. We included a large number of HVGs for Progression Cohort analysis to ensure that we retained important pancreas and cancer-associated genes and captured the spectrum of normal and disease states. To analyze subsets of this cohort, including specific stages or clusters, we chose fewer HVGs to focus on variability specific to that set (see **Table S13** for description of each analysis and parameters).

Next, we performed PCA on the filtered, log-normalized Progression Cohort matrix restricted to 8,000 HVGs. We used automatic knee point detection based on the cumulative variance explained per component to determine a cutoff for the number of PCs to carry forward, selecting 49 top PCs which explained ~41% of the variance. We further verified that adding subsequent PCs has trivial impact on the overall variance explained (inclusion of 50 additional PCs explains <3% further variance). To visualize the full map in 2 dimensions, we ran tSNE using the bhtSNE package (*89*) on selected PCs with perplexity = 150 and theta = 0.5. We then ran diffusion maps and selected 13 diffusion components (DCs) via Eigen gap to obtain a non-linear embedding of cells for downstream analysis.

To assess whether the DCs represent known PDAC biology, we examined how published signatures of normal regeneration and disease states were distributed across DCs. We obtained published gene sets derived from bulk RNA-seq of human tumors and matched normal samples. Differentially expressed genes (DEGs) between human PDAC vs human normal samples were

determined by > 2-fold change in gene expression with adjusted p value < 0.05; (*34*, *35*), as well as in complementary mouse models (DEGs between murine PDAC vs. normal, determined by > 2-fold change in gene expression with adjusted p value < 0.05; (*23*)). For human gene sets, we mapped orthologous gene names to obtain equivalent mouse gene sets with Ensembl annotations. We then obtained z-scores for each gene to scale log-normalized features for comparison, and computed the average z-score across all captured genes per set. We visualized these scores for each gene set and sorted cells by DC 1, which roughly captures the order of progression determined from our scRNA-seq data (**Fig. 1C**). This visualization supports that diffusion maps derived from our unbiased Progression Cohort analysis effectively capture biologically meaningful axes of disease state variation.

Our next step was to define discrete cell types within the phenotypic map by clustering with PhenoGraph on selected PCs using the Leiden algorithm for community detection. First, we performed a grid search over values of k and clustering resolution to ensure robustness to these parameters. Specifically, we computed the pairwise Rand index between each pair of clusterings and found that the clusters are similar within small windows of parameters (small perturbations in k had little impact on the clustering within reason; Rand index > 0.9 for nearly all combinations of $k = 35 \ldots 50$ for fixed resolution = 0.5). We chose one coarse clustering (k = 35, resolution = 0.5), which captures major populations (for instance, grouping all neuroendocrine populations) (**Fig. S1B**) and one more refined clustering (k = 50, resolution = 1.0) which captures highly resolved structure (separating clusters along resting-to-injured axes) (**Fig. S1C**). We annotated clusters (**Figs. 1B** and **S1B,C**) by inspecting DEGs and boxplots of log-normalized gene expression across clusters to identify cell types based on the marker lists in **Table S2**. For known intermediate cell-states (for example, Acinar-to-ductal metaplasia, or ADM), a

combination of these markers may be expressed. Other markers are expressed by a more specific subset of the cells assigned to each coarse state (for example, *Ins* expressed in rare pancreatic cells amongst Neuroendocrine-like cells). Drop-out is observed for lowly expressed genes (such as transcription factors *Ptf1a* and *Bhlha15* in acinar cells).

Finally, we also computed separate embeddings and analyses on subsets of the Progression Cohort, including individual stages or clusters (**Table S13**). In all cases, we performed the same steps above for gene selection, dimensionality reduction and visualization on the log-normalized count matrix of that subset, so as to capture the HVGs and major axes of variation within each group. To maintain consistent cell type annotations, however, each subset analysis retained the original PhenoGraph clusters computed on the entire Progression Cohort (**Fig. S1B**). In some cases, we chose to visualize a subset with a force-directed layout (FDL) built on a cell-cell affinity graph which accounts for uneven densities along the cellular manifold as in MAGIC (*69*). In brief, we first computed a k-nearest neighbor (kNN) graph on cells based on Euclidean distance between points in PC space using k = 30 by default. This kNN graph was then converted to a cell-cell affinity matrix by applying an adaptive Gaussian kernel (width = 10 for k = 30) to edges on the graph and symmetrizing the graph, from which an FDL was computed using Harmony's plot.force_directed_layout function (*90*). This visualization emphasizes the non-linear continuous structure of the phenotypic manifold measured by a graph, as opposed to tSNE built on PCs, which emphasizes separation of cell-states across linear dimensions. The choice between FDL and tSNE for each visualization was based on the expected continuity of discrete cell-states across the progression. For instance, acinar cells of the basal pancreas are expected to be extremely distinct from cell-states of the malignant pancreas. In this case, any spurious edges in the cell-cell affinity graph connecting basal acinar cells to pre-malignant or malignant (K1–

K6) cells may inappropriately display a continuity between these populations. Parameters used for each visualization and analysis metrics for the Progression Cohort subsets are noted in **Table S13**.

Perturbation Cohort, shIL33 epithelial cells sorted on mKate2$^+$GFP$^+$CD45$^-$: The Perturbation Cohort consists of epithelial *Il33* depleted and control samples to address the effects of cytokine signaling on progression. Individual datasets from this cohort were combined according to stage (K2/K2-sh*Il33* and K3/K3-sh*Il33*) to compare across perturbation and control conditions at each time point, resulting in separate K2 and K3 matrices. We filtered data from each stage for low library size and contaminating mesenchymal cells, as well as for lowly expressed genes (captured in <20 cells) and for ribosomal and MT genes, and log-normalized as described for the Progression Cohort. PCA was applied to 5,000 HVGs in each stage determined using the approach from (*88*) as above. Using automatic knee point detection, we retained 53 PCs describing 28% of the variance for stage K2 and 51 PCs describing 33% of the variance for stage K3.

Immune Cohort (K1–K5 & shIL33 immune cells, sorted based on CD45$^+$mKate2$^-$): The immune data was processed with slightly different steps compared to the epithelial data, due to its distinct structural features and the extensive knowledge available on immune subsets. Briefly, we performed the initial preprocessing in two different batches hereafter referred to as Immune Cohorts 1 and 2 (corresponding to Filtering Groups 5 and 6, respectively, **Table S12**) because these samples were acquired at different timepoints. After removing low quality cells and doublets in each Immune Cohort, we merged Immune Cohorts 1 and 2 to jointly annotate cell types thereby increasing power in detecting rare cell types and ensuring consistent annotations

across samples. Cell type annotation was performed in a hierarchical manner first annotating major immune subtypes (T cells, B/plasma cells, myeloid cells) by clustering on the dominant PCs only. After partitioning the data into major immune subtypes, re-normalizing and re-clustering the data on a higher number of PCs, we annotated granular cell types within each major immune subtype. The annotated data was then combined with the epithelial data for the cellular crosstalk analysis. The detailed parameters of this analysis are outlined below.

Immune cohorts 1 and 2 were processed separately as outlined for the Progression Cohort above. After this initial processing, we detected heterotypic doublets (for example, cells expressing both *Cd3e/Cd3f/Cd3g* and *Cd19/Ms4a1/Ighd*) and therefore introduced a doublet filtering step. Our strategy was based on the idea that doublets will form distinct clusters in phenotypic space, and we chose to cluster cells from all samples within each Group (rather than individually) to ensure that smaller clusters would not be lost.

To prepare for clustering, we filtered genes detected in fewer than 20 cells, as well as MT and ribosomal genes, and cells expressing fewer than 200 genes. We normalized gene expression to median library size and log1p-transformed the data instead of employing scran normalization (used for cell type annotation, below) to avoid nonsensical scran size factors (size factor <0) which may be assigned to a minority of low-quality cells. We then computed HVGs using scanpy's scanpy.pp.highly_variable_genes function with the seurat_v3 method on raw gene expression counts. However, this method tends to discard genes relevant to cell type annotation, which results in worse cell type separation among the resulting clusters. We therefore added a manually curated list of marker genes to the computed list of HVGs for downstream applications including PC calculation and tSNE embedding (**Table S14**). This list includes 425 key genes for

discriminating cell identities or functions of T cells, innate lymphoid cells including NK cells, B cells including plasma cells, and myeloid cells including dendritic cells. After testing a range of 5,000–15,000 HVGs and all genes, we selected 10,000 HVGs and added the 425 marker genes to calculate PCs for both Immune Cohorts, as this led to the best separation of easily confounded cell types in clustering (particularly dendritic cells, mast cells, innate lymphoid cells and plasma cells).

We then clustered the data on PCs using PhenoGraph, selecting the number of PCs by the knee point of the PC vs. explained variance curve (calculated using the kneed package v0.7.0), or the fewest PCs that explain >20% of total variance, whichever is higher. Based on this procedure, PhenoGraph was calculated on the first 9 PCs explaining 23.2% or 6 PCs explaining 22.8% of total variance for Immune Cohorts 1 and 2 respectively. We selected $k = 10$ for kNN graph construction because this most clearly delineated clusters containing 20% or more doublets from those with lower doublet content.

To call doublets, we applied DoubletDetection to each immune cohort, and in addition, re-ran DoubletDetection for each sample in Immune Cohort 2 because this detected additional doublets. To filter called doublets, we first eliminated entire clusters comprising >20% doublets, then removed all remaining annotated doublets. We made sure not to remove entire cell subpopulations with biologically sensical phenotypes. Moreover, we removed remaining clusters of obvious heterotypic doublets manually (for example, cells expressing both *Cd3e/Cd3f/Cd3g* and *Cd19/Ms4a1/Ighd*); these generally overlapped with DoubletDetection annotations but did not reach the 20% threshold. Using this procedure, we removed 3,062 and 349 cells, yielding a final dataset of 22,658 and 12,768 cells for Immune Cohorts 1 and 2, respectively.

To prepare for cell type annotation (**Fig. S9A)**, we combined Immune Cohorts 1 and 2 to increase the power to detect rare immune subsets, and to maintain consistent annotation across analyses. We first partitioned the immune data into major subsets (T/ILC/NK cell, B cell, myeloid cells), focusing on the first few dominant PCs which separated these subsets, and performed finer clustering and annotation on each subset individually as outlined below.

We used scran to normalize the count matrix of the combined Immune Cohort, because median library size normalization can artificially generate differential expression between cells with highly differing library sizes and rates of drop-out, such as leukocytes. Scran circumvents this problem by normalizing cells using cluster-specific size factors (*91*). After normalizing raw counts by scran and log1p-transforming the data, we re-calculated HVGs and added the 425 marker genes to the top 7,500 highly-variable genes, which we used to calculate the top 6 PCs explaining 23.6% of total variance. This small number of dominant PCs was used to define the major immune subtypes, whereas normalization and clustering of refined subsets involved many more PCs (see below and **Table S15** for parameters used in Calligraphy analysis). We clustered the data in PC space using the immune cell workflow described above and PhenoGraph k = 50, and ensured that the clustering was stable in a window of adjacent parameters as explained above with a pairwise Rand index of > 0.7.

To assign cell type labels, we manually assessed patterns of mean z-scored marker gene expression in clusters using our custom marker genes (**Table S14**). We also calculated DEGs for each cluster versus all other clusters with scanpy's scanpy.tl.rank_gene_groups function using the Wilcoxon rank sum test with Benjamini-Hochberg correction. This simple differential expression method was chosen for scalability, given the number of comparisons required for cell

type annotation. We then calculated a Szymkiewicz-Simpson overlap coefficient (size of the intersection divided by size of the smaller set) between the top 1,000 DEGs and each marker gene set to assign cell type labels to clusters. This procedure separated leukocytes into three major populations: T/ILC/NK, B/plasma and myeloid cells.

To prepare for more granular cell annotations, we partitioned immune subpopulations and repeated normalization and clustering for each subset separately. We normalized raw counts using scran, log1p-transformed the data, then repeated the calculation of HVGs and added cell-type-specific marker genes (T/ILC/NK, B or myeloid markers) to the top 7,500 HVGs. PCA yielded 48 PCs explaining 20.09% variance for T/ILC/NK cells, 69 PCs explaining 20.05% variance for B cells and 50 PCs explaining 28.9% variance for myeloid cells. We re-clustered the data with PhenoGraph with $k = 40$ for T/ILC/NK cells, $k = 30$ for B cells and $k = 50$ for myeloid cells, choosing a k value that gives stable clustering in a window of adjacent k parameters as explained above (pairwise Rand indices > 0.7).

We next iterated our annotation procedure using marker genes specific to subtypes of T/ILC/NK, B or myeloid cells (**Table S14**). The rationale for our iterative partitioning approach is based on (1) computational considerations, as it removes major cell-type-specific signals which may confound more granular subtyping, and (2) our biological knowledge of granular cell type markers, which is often limited to comparisons within major subpopulations; for example, differentially expressed markers between tissue-resident memory (TRM) and naive T cells are well defined, but we know less about how naive T cell markers and TRM markers differ from plasmacytoid dendritic cell markers. A notable drawback of this approach is that during re-normalization with scran, some cells with lower library sizes (including granulocytes and naive T

cells) can obtain nonsensical negative size factors, leading to automatic removal. These cells could not be annotated on a more granular level and were removed from further analyses (1.04 % of all cells, mostly lower-quality T cells). We also made sure that no known immune cell subpopulations were removed in their entirety by this caveat of scran normalization. Final immune cell annotations are presented in **Fig. S9A**, along with their marker expression patterns in **Fig. S9C**.

Finally, downstream crosstalk analyses were based on these annotated immune cell subsets derived from samples selected for comparison with pre-malignant Progression Cohort ("pre-malignant immune integration" set) with distinct processing parameters described in **Table S13**.

Combined embedding of immune and epithelial data: In addition to the above analyses, we generated one combined embedding including subsets of both Immune and Progression Cohorts to visualize patterns of crosstalk between epithelial cells and their microenvironment (see "Heterotypic cell-cell communication analysis" below). For this analysis, we combined cells of the Progression Cohort pre-malignant Immune Integration (K1–K3) set (**Table S13**) and the three immune subsets above (**Table S15**). We log-normalized the combined data with standard library size normalization, computed 8,000 HVGs as above, and performed PCA selecting 40 PCs explaining ~36% of the overall variance. tSNE was applied to these PCs with perplexity = 150.

Human scRNA-seq data analysis: In addition to the above GEMM-derived data sets collected in this study, we analyzed previously published data from primary tumors of patients and normal pancreas. We acquired a processed scRNA-seq count matrix combining across samples from

(*32*). Median library size normalization was performed. MT and ribosomal genes were removed, as well as genes expressed in fewer than 20 cells and *MALAT1*.

The dataset contained both abundant stromal cells including fibroblasts and endothelial cells as well as immune cells expressing CD45. To exclude these from the analysis for comparison with our epithelial (mKate2$^+$) murine datasets, we performed PhenoGraph clustering with k=100 to capture major subsets and removed stromal clusters (expressing *LUM*, *PLVAP*, and low levels of epithelial markers *CDH1* and *EPCAM*) and immune clusters (expressing CD45 plus subset specific markers such as *CD79A*, *CD3E*, *MS4A1*). The resulting dataset consisted of 20,386 epithelial cells and 20,830 genes for downstream analysis.

Ptf1a-CreER scRNA-seq data analysis: We sought to validate the heterogeneity observed in our mouse model (with Cre- driven embryonic activation of mutant *Kras*) in an alternative model with adult activation of oncogenic *Kras* (*Ptf1a*-CreER). To this end, we obtained a publicly-available scRNA-seq count matrix from Schlesinger and colleagues (*52*). Using the cell count matrix and cell-type annotations from the original paper, we began by first filtering non-epithelial clusters, retaining only clusters 4, 15, 18, 9. We applied an additional filter to remove any remaining cells negative for tdTomato expression, which marks *Ptf1a*-expressing acinar cells with mutant *Kras*. With these filters, we are left with a set of 5,303 cells comparable to those collected in the current study (acinar originating -epithelial cells), but with adult activation of *Kras* mutation. We then applied basic processing steps as above, first filtering any genes expressed in fewer than 20 cells, removing *Malat1*, and log library size normalizing to a scale of 10,000 counts per cell. With the approach above described in (*88*), we selected 1,000 highly variable genes, on which we computed 61 PCs explaining 62.35% of the variance.

To compare the heterogeneity within this published dataset to that observed in newly-generated scRNA-seq data, we began by clustering published data on selected PCs using PhenoGraph with default parameters (k=30). We took the intersection of genes identified in both datasets, and computed the Pearson correlation pairwise between each cluster in the published dataset and each in the newly -collected data (coarse PhenoGraph clusters in **Fig. S1B,** restricting to most comparable conditions K1-K3). This was done excluding clusters in the newly collected data which were exceedingly rare in conditions K1-K3 (< 200 cells). These pairwise correlations are presented in **Fig. S8A**, which shows a high degree of correspondence between cell-states across each dataset.

Comparing across cell annotation sets: The above scRNA-seq analyses generate layers of annotations for each cell based on sample of origin (for example, cells collected from mice harboring *Kras*-mutant allele and subjected to pancreatic injury are labeled "K2") and on the outputs of our analyses (for example, each cell is assigned to both a refined and coarse PhenoGraph cluster). We leverage different annotation sets (sometimes more than one) depending on the goal of each downstream analysis, with the specific annotation(s) labeled in the corresponding figure panel, legend, or methods description. While the different annotations do not map one-to-one, they are related; we clarify how they overlap by visualizing each on our entire Progression Cohort dataset, coloring each cell by a single annotation in **Figs. 1B** (stage of progression), **S1B** (coarse PhenoGraph cluster), **S1C** (refined PhenoGraph cluster), and **S2D** (individual biological replicate). Most refined clusters largely map to a single coarser cluster, as visualized in **Fig. S1B,C**, which indicates nomenclature used in the manuscript. The clusters span multiple states in our experimental design and the text indicates when these are stage-

specific or stage-enriched. We also quantify overlap between these sets by visualizing distributions of cell stage per PhenoGraph cluster in **Fig. S1D**.

Each of the four apex states (see section "Prediction of initiating pre-malignant states with scVelo and CellRank") can be mapped exclusively to a coarse cluster: apex "Nes^+ Origin" to cluster 1, apex "$Tff2^+$ Origin" to cluster 0, apex "$Aldh1b1^+$" or "Neuroendocrine-like Origin" to cluster 9, and apex "Differentiated Acinar Origin" to cluster 3 (see **Fig. 2A**). No apex cells are derived from any cluster beyond these four. Metacells (see section "Metacells algorithm") are assigned to a single, dominant refined cluster for downstream analysis. The refined clusters for each metacell are color-coded based on cell-state annotation in **Fig. S6A** and in the top row and right column in **Fig. 3C**, such that metacells from two distinct clusters will be colored the same when their annotation is the same.

Prediction of initiating pre-malignant states with scVelo and CellRank

Unbiased analysis of the Progression Cohort with diffusion maps reproduced the general chronology from normal (N1) to malignant (K5, K6) states (**Fig. 1C**). However, substantial heterogeneity within each pre-malignant condition, as well as overlapping cell-states across distant time points (for example, mixing of neuroendocrine-like cells across K1–K3 conditions), suggests that cells are undergoing state transitions that cannot be defined by time series information alone. Most pseudo-time approaches assume cell-states transition from less to more differentiated states from a known origin (*61*). However, regeneration and tumorigenesis involve both differentiation and de-differentiation and origins are not well characterized. Thus, we chose

to use CellRank (*39*), an approach which integrates multiple lines of information to infer directionality in an unbiased manner (without strong assumptions about origin populations or directionality typically required for trajectory inference (*61*)).

The first input to CellRank are RNA velocity (*39*, *41*) vectors, which have been shown to successfully infer directionality in systems of differentiating cells using ratios of spliced and unspliced transcripts. The RNA velocity vector of a given cell predicts which genes are currently being up- or downregulated and points towards the likely future state of that cell. However, RNA velocity is only able to capture short term transcriptional dynamics (~1 day) and we therefore limited our analysis to the earliest time points (K1 and K2). We used the pre-processing functions of Velocyto (*41*) to efficiently separate reads from K1 and K2 BAM files into spliced and unspliced count matrices, and to generate loom files for downstream velocity algorithms. However, Velocyto assumes that a subset of cells are in steady state, which is unlikely to be true of cells responding to oncogene activation and/or injury as in our case; we thus opted to use the scVelo algorithm (*40*), which can model expression dynamics which are not in steady state. We first applied log-normalization and filtering of lowly expressed genes (<20 spliced and/or <20 unspliced transcripts) in scVelo to retain 9,294 genes. We then applied the scVelo velocity estimation in "dynamic" mode with default parameters on this subset of genes.

However, individual velocity estimates are known to be very noisy and these individual vectors do not reveal origin states. Moreover, projecting the high-dimensional velocity vectors to 2D often fails to faithfully capture the global transcriptional dynamics. To infer global dynamics of the system, CellRank combines gene expression similarity with RNA velocity to robustly estimate directed trajectories of cells. The robustness is gained through the use of the similarity-

based neighbor graph and cell-state transitions are modeled as directed random walks along this graph. The more a neighboring cell lies in the direction of the velocity vector, the higher its transition probability. However, unlike RNA velocity, all transitions are enforced to remain within the phenotypic manifold. By globally modeling the cell-state transitions as a Markov chain, CellRank is able to successfully identify initial ('apex') states, inferred to be the sources of the cell-states observed in the data.

Previous authors have noted that velocity algorithms are most appropriate for capturing time scales on the order of hours *(41)*. For this reason, we limited CellRank analysis to early timepoints (K1-K2) which are most relevant for this time scale. K1-K2 timepoints are also expected to be the most dynamic, as cells are actively responding to acute inflammation. Importantly, because we only applied CellRank in these early timepoints (where robust endpoints may not be present), we chose not to apply full trajectory analysis (identification of distinct branches and endpoints) to avoid spurious detection of endpoints. We thus ran CellRank strictly for initial state detection with default parameters, identifying four high-confidence originating apex populations with an initial state probability cutoff automatically determined by the algorithm's eigengap-based threshold (**Figs. 2A** and **S3B**). These four apex states overlap exclusively with coarse PhenoGraph clusters 0, 1, 3, and 9 (see **Fig. S1B**). In other words, all cells annotated as apex states by CellRank's initial state probability cutoff belong to these clusters. A set of marker genes, derived from populations whose initiating potential was discerned through Cre-mediated lineage tracing of neoplastic pancreas in previous works (*26*, *42–45*), exhibited strong concordance with CellRank-predicted initial states (**Fig. S3C**). As a control, the above steps were followed identically for application of CellRank to regenerating

pancreatic epithelia (N1-N2). This identified only a single high-probability apex state associated with an acinar-like state.

We ensured robustness of this approach by performing a downsampling analysis, each time randomly sampling the dataset to 75% of its original size and repeating the above steps for velocity and CellRank inference. For each of 500 such trials, we recorded the CellRank initial state probabilities and the individual apex states found by thresholding these probabilities. We found that CellRank's per-cell initial state probability (probability that a cell is an apex state) is highly stable (average Pearson correlation vs. full dataset inference r=0.6474). The discrete states that come out from thresholding this probability are concordantly very stable: an acinar initial state (corresponding to coarse PhenoGraph cluster 3) exists in 100% of the trials, and a Nes^{+} progenitor state (corresponding to coarse PhenoGraph cluster 1) exists in 90% of the trials. No additional apex states beyond those reported in the paper were found in the vast majority (90%) of trials.

Bulk ATAC-seq data generation, processing and integration

The substantial heterogeneity generated from high-potential oncogenic states identified by CellRank occurs rapidly (24–48 hpi), a time scale that is more consistent with changes in chromatin accessibility than genetic mutation. To test whether epigenetic features can explain observed scRNA-seq heterogeneity, we integrated bulk ATAC-seq data from published studies for stages N1-N2, K1-K2, and K5 (*23*). We also collected additional samples from intermediate pre-malignant conditions K3-K4 to complete the Progression series. To do so, we generated new

epigenomic data from mKate2+ cells FACS-isolated from benign neoplasia (PanIN) tissue states (K3, K4), as previously described (*23*), and analyzed it with previously generated bulk ATAC-seq profiles from mKate2+ cells isolated from pancreata representing normal (N1), regenerating (N2), pre-neoplastic (K1, K2) and malignant (K5) states (extracted from GSE132440).

These data sets were processed as described in (*23*). Briefly, trimmed paired-end reads were aligned to mm9, peaks were called for each sample individually with MACS2 (*92*), and a global atlas was derived by merging peaks from each sample within 500 bp of one another. Read counts within each peak were normalized with DEseq2 (*93*) to account for differences in per-sample sequencing depth.

Identification of chromatin modules describing progression: We sought to learn broad accessibility patterns over stages, and to map these to single cells. We first called differentially accessible peaks across early stages (N1-N2, K1-K4) in our bulk ATAC-seq data using DEseq2 (log_2-transformed fold change ≥ 0.58 and FDR ≤ 0.1). To identify global progression trends relevant to early progression, we performed PCA on normalized differential peaks contrasting all pre-malignant conditions pairwise, then visualized the first two PCs explaining ~54% of total variance (**Fig. 2B**). The distribution of samples in this embedding suggests consistent, distinct genome-wide accessibility patterns defining replicates (individual mice) of the same stage.

Next, to study the specific accessibility patterns underlying these trends, we applied kmeans with n = 15 clusters to peaks based on the z-scored accessibility across samples. This clustering organized peaks into several dominant patterns of regulatory dynamics (henceforth “chromatin modules”), comprising peaks only accessible in normal pancreas (Normal chromatin module, n = 20,140 peaks), peaks which become most accessible in early PanINs (Benign Neoplasia

chromatin module, n =26,122 peaks), and peaks which become most accessible in PDAC (Malignant chromatin module, n = 23,145 peaks) (**Fig. 2C**). Notably, we observed that peaks of the Benign Neoplasia module are closed in malignancy, despite the chronological relationship between pre-malignant PanIN lesions (K3, K4) and their eventual malignant PDAC states (K5, K6). However, some trends exist across modules: both Benign Neoplasia and Malignancy-associated peaks are accessible to some extent in K1 and/or K2 before becoming predominantly accessible in K3-K4 and K5-K6, respectively.

To group peak clusters according to these patterns, we removed clusters from downstream analysis if they did not clearly fit in one of the 3 modules of interest (discarding 11,483 peaks), and visualized the resulting peak groups by heatmap, where values are normalized with DEseq2 and z-scored for each row to highlight changes in accessibility along progression (**Fig. 2C**). Manual curation was preferred over automated cluster grouping to ensure that clusters lacking coherent patterns over progression were not included in each module.

Integration of bulk chromatin with single-cell RNA-seq: We investigated chromatin-directed gene expression at the single-cell level by integrating bulk-derived chromatin modules with K1–K6 scRNA-seq data from the Progression Cohort (**Table S13**). First, we mapped peaks to associated genes based on distance as in (*23*). We then aggregated all genes mapping to peaks associated with each module, yielding three module-associated gene signatures. We identified distinct genes in each signature to define mutually exclusive patterns associated with each chromatin module (**Table S3**). Finally, we z-scored the expression of each individual gene across each cell to emphasize those that are relatively overexpressed in each cell, and visualized each module's relative activity as the average z-score of the genes in that module (**Fig. 2C**, right).

Predictive model of late-stage fates

Our analyses of chromatin dynamics generated three complementary observations: (1) a divergence in PC space between Benign Neoplasia (K3, K4) and Malignant (K5, K6) stages (**Fig. 2B**), (2) an early increase in accessibility (K1, K2) of regulatory elements that become highly accessible in Benign Neoplasia and Malignant modules (**Fig. 2C**), and (3) an association of early (K1, K2) cell-state expression with one or more late-stage modules. Based on this, we hypothesized that *Kras*-mutant cells are epigenetically primed, such that their early chromatin accessibility patterns establish propensities for Benign and/or Malignant fates. To define epigenetic priming in concrete terms, we assert that cells primed for different fates can be discerned by the presence of open chromatin proximal to fate-specific genes, prior to the establishment of fate-associated states.

To quantitatively evaluate this hypothesis, we first assumed that gene expression is a reasonable surrogate for accessibility of that gene's regulatory elements for at least a subset of genes, an assumption which derives from the observed correspondence between expression state and chromatin modules derived from bulk ATAC-seq (**Fig. 2C**). We thus aimed to devise a computational framework which can identify transcriptional signatures that discriminate between Benign and Malignant phenotypes, then use it to probe for these programs in early tumorigenesis. To this end, we trained a classifier to distinguish Benign and Malignant fate labels using single-cell samples from late timepoints (PanIN K3, K4 and PDAC K5, K6). The resulting expression-based predictive model of cellular fate was used to interrogate cells from early pre-neoplastic time points (K1, K2) for these fate-associated programs. We assume the

inferred probability distribution of distinct fates for each early cell reveals its tendency to skew toward particular expression endpoints.

Specifically, we used a logistic regression multi-class classifier on log-normalized expression features to predict stage labels. We randomly split late-stage cells (K3–K6) into a training set (10,758 cells, or 80% of late-stage data) and a held-out validation set (2,689 cells/20%). We trained the model with sklearn (*94*), which provides a maximum likelihood estimate of logistic regression parameters to predict stage labels (K3, K4, K5, or K6) in the training data. Our model performed well on held-out data (accuracy = 0.9966, precision = 0.9973), suggesting that gene expression features are informative of late-stage cell fates and that our classification strategy captures fate-relevant patterns. We further inspected coefficients of our fitted models to identify strongly predictive genes for each late-stage phenotype, finding expected programs such as EMT (*Epcam and Vim*), MYC activity, and tumor suppressor activity distinguishing Benign from Malignant phenotypes (**Fig. S4B**).

Next, we applied our trained classifier to pre-neoplastic (K1, K2) cells, and visualized the per-cell probabilities of each fate category. We collapsed the classifier into Benign and Malignant probabilities by summing probabilities for K3-K4 and K5-K6 cells, respectively (**Figs. 2D** and **S4C**). We then binned early cells into categories: highly associated with a Benign state (Malignant probability <0.4); highly associated with a Malignant state (Malignant probability >0.6); and mixed state (0.4 <= Malignant probability <= 0.6), representing rare cells (<5% of K1-K2 cells) that express a composite Benign-Malignant program (**Fig. 2D**). We find uninjured (K1) cells are enriched in the Benign class, while injured cells are enriched in the Malignant class (Fisher's exact test, odds ratio = 17.3671, p value = ~0.0), recapitulating previous reports

that injury shifts the oncogenic pancreas epithelia toward a cancer-like state (*23*). To visualize intermediate states, we computed the density of these cells in PC space (using selected PCs, described above in "Single-cell RNA-seq data processing") with a Gaussian kernel density estimate via scipy.stats.gaussian_kde with default bandwidth parameters, which we visualized in two dimensions on a FDL (**Fig. S4D**).

Intermediate or fuzzy classification to one or more states can occur for one of two reasons: cells either simultaneously express programs of more than one class, or cells can express neither class-associated program. As such, it was important to ensure that both Benign and Malignant programs were detectable in a majority of our mixed probability cells (0.4 <= Malignant probability <= 0.6) to designate them as composite states. We developed a gene signature for each program by extracting the genes with the top 200 coefficients (by coefficient value) amongst Benign (K3) and Malignant (K5) logistic regression models. We then intersected these gene lists with Benign chromatin module and Malignant chromatin module genes respectively to ensure that these transcriptional programs were primarily associated with underlying epigenetic differences. We then computed a separate Benign and Malignant signature score per cell by taking the average z-scored expression of each gene in the signature. **Fig. S4E** confirms that true Benign (K3, K4; pink contour) and Malignant (K5, K6; purple contour) cells are clearly distinguishable based on these values, and further shows that the majority of mixed probability cells (orange) assume an intermediate value between these modes. Importantly, many of these mixed cells highly express both programs, suggesting that many assume a truly composite phenotype with respect to late-stage fates.

Single-cell ATAC-seq processing and analysis

Initial processing: To extend and support our analysis of chromatin dynamics, we analyzed scATAC-seq data from comparable or paired (in other words, from the same mouse, **Table S16**) pre-malignant and malignant conditions (K1–K3, K5). Two samples were derived from a previously published study (*23*) and the remaining were collected for this work as described above.

Samples were processed individually using a modified CellRanger ATAC pipeline as described in (*23*) to derive alignments to mm10, then analyzed with ArchR (*65*), a tunable pipeline for filtering, normalization, dimensionality reduction and visualization of aligned scATAC-seq fragments. We chose ArchR for its flexibility in representing accessibility features in defined regulatory elements, proximal to genes of interest, or globally in bins along the genome. This presents an advantage over approaches focused on accessibility solely in inferred regulatory elements (called peaks), which may disregard dynamics driving rare cell populations (from false negative peak calls) or erratic dysregulation that may occur in cancer (resulting in many noisy peak calls). We applied a custom modified version of ArchR (*47*) which is better suited to capture chromatin features of rare populations, as described below.

To begin, individual CellRanger ATAC fragment files from all stages (K1–K3 and K5) were combined into a single dataset in ArchR, and basic filtering was performed with parameters filterFrags = 8000 (determined by manual inspection of the fragment count distribution) and maxFrags = 1e+7. Doublet score computation and doublet filtering were performed with default parameters, resulting in 19,666 cells. For consistency with scRNA-seq analysis, we removed

mesenchymal contaminants, yielding a final count of 18,211 cells. We then derived the two standard ArchR feature representations for our scATAC-seq data:

> "Tile Features" bin counts in 500 bp windows across the accessible genome, which allows flexible representation of the data without peak calling error or bias toward coding regions. Tile features tend to produce embeddings with the most faithful phenotypic structure, hence we used them for major downstream analyses such as dimensionality reduction, visualization and clustering.

> "Gene Score Features" are distance-weighted aggregations of accessibility proximal to each gene that allow functional interpretation of the data. Using our modified pipeline, we only computed gene scores on regions identified as highly variable across clusters to improve the extent of heterogeneity captured by these values. We used the resulting scores for cell type annotation and integration with scRNA-seq features (See "Identifying and integrating pancreas metacells" below).

Dimensionality reduction and visualization: To compute a low-dimensional embedding of single cell epigenomes, we applied the standard approach for dimensionality reduction implemented in ArchR, which involves Latent Semantic Indexing (LSI). This approach, designed to handle low-frequency count data commonly encountered in document analysis, is better suited to analysis of scATAC-seq data compared to PCA, which implicitly models the data as a continuous-valued Gaussian. This reflects the near-binary and highly sparse nature of scATAC-seq data (maximum 2 reads per transposition site), which is very dissimilar from bulk data (upwards of 50 million reads per profile). Specifically, we applied ArchR's iterative LSI function to the tile matrix on 150,000 highly variable features. Each round of LSI generates a preliminary low-dimensional

embedding from which a clustering is estimated. Variable tile features are then computed across clusters to select the most informative tiles with respect to the major phenotypes. In our case, we modified parameters in each LSI iteration to capture more refined cell types; a clustering resolution parameter of 0.1 distinguished small cell populations (for example, tuft cells) better than default parameters. LSI components from ArchR were then used as input to compute visualizations, and for downstream analysis in python. As described above (see "Single cell RNA-seq data processing"), we computed an FDL on the cell-cell affinity matrix constructed from Euclidean distances in LSI embedding coordinates (as opposed to PCs used for scRNA-seq) (**Figs. 3A** and **S5A,B,D**). LSI components are directly visualized in **Figs. 3B** and **S5B**.

Clustering and annotation: To then identify discrete cell types, we clustered scATAC-seq data with PhenoGraph (k=30) using the Leiden algorithm on LSI component features. At this level of resolution, we achieved desirable granularity with respect to rare cell types (for example, tuft cells), but also observed several groups of highly-related clusters with shared accessibility at key markers (**Table S17**) which may potentially originate from a single cell type. To quantitatively identify these phenotypically-similar clusters, we examined each initial PhenoGraph cluster for overlap in accessibility patterns proximal to genes. We first performed a Wilcoxon rank test (the default approach in ArchR for differential analysis) for each gene's accessibility in one cluster versus all others based on values in the gene score matrix (described above) using the getMarkerFeatures function. For each cluster, we filtered the resulting significant genes (FDR<=0.01) to retain those with a log2 fold change of at least 1.25 as marker genes. We then computed the percentage of shared marker genes between each pair of clusters, indicating extent of phenotypic similarity between clusters at the chromatin level. We observed substantial sharing of marker genes between several pairs of clusters (minimum 15% shared genes), which we

reasoned could be merged for annotation and downstream analysis. Resulting merged clusters are displayed in **Fig. S5A**.

We then identified marker genes for the merged clusters once again following the Wilcoxon rank method on gene scores in one merged cluster versus all others. We visualized shared marker genes between pairs of merged clusters as before and observed elimination of high inter-cluster sharing (<15% shared), thus confirming the merged clusters as highly phenotypically distinct. Lastly, to identify the cell type of each cluster and further validate the merging, we computed the average gene accessibility scores of every marker gene and compared the results against known biological signatures for various pancreas populations. We identified individual merged clusters associating with gastric-like, *Nes*$^{+}$ progenitor-like, PDAC, neuroendocrine-like, ADM, and tuft cell populations, in concordance with our scRNA-seq analysis, according to the annotation criteria in **Table S17**.

ATAC-seq signal tracks: We visualized signal tracks of scATAC-seq profiles aggregated over clusters or samples using custom python code utilizing the tabix package (*95*) (**Figs. S5C** and **S7A**). For each track, we aggregated reads from fragment files in a 5 kb to 50 kb window around a gene, and normalized coverage to the total depth of cells included in the cluster or sample set of interest, to provide comparable scale across tracks. Coverage was smoothed using a window size of 100 bp to aid visualization.

Metacells algorithm

Clustering and cell-type annotation suggest that cell-states derived from scRNA-seq and scATAC-seq data match at the broad cell-type level, but we also find substantial heterogeneity within each cluster. For example, the *Nes*$^+$ Progenitor population (coarse PhenoGraph Cluster 1) includes a spectrum of phenotypes primed for distinct fates (PDAC probabilities spanning from nearly 0 to 0.9999). We therefore needed a higher-resolution mapping between epigenetic and transcriptional states, yet the extreme sparsity of scATAC-seq hinders our ability to characterize chromatin states of individual cells. Our first goal for data integration is thus to overcome the sparsity of single-cell profiles while maintaining a high-resolution of diversity in cell-states.

We addressed this challenge by developing an algorithm that aggregates cells which share the same state at an intermediate resolution between single cells and typical cell-state clusters, inspired by the metacell approach (*46*, *47*). Each metacell represents a distinct, highly granular and homogenous cell-state, such that differences between cells within a given metacell are due to technical noise rather than biological disparities. As such, aggregating counts across cells in each metacell overcomes noise and dropout, providing a robust characterization of that distinct cell-state. The concept of metacells was introduced by Baran and colleagues (*46*), but the accompanying method is unsuitable for scATAC-seq, and it removes outliers aggressively. Our algorithm is non-parametric (requiring little parameter tuning by the user) and flexible to diverse modalities, including both scRNA-seq and scATAC-seq. In this work, we aimed to derive metacells from epigenomic and transcriptomic data separately, so that they could then be reliably integrated across the span of progression.

Our algorithm first constructs a kNN graph over cells, determining neighbors based on Euclidean distances in PC space for scRNA-seq or iterative LSI space for scATAC-seq (computed as in "Single-cell RNA-seq data processing" and "Single-cell ATAC-seq processing" above). The algorithm then constructs a shared nearest neighbor (SNN) graph by pruning edges in the kNN graph that are not bidirectional. The remaining edges are mutually highly similar to one another and better reflect the phenotypic manifold; they are weighted using an adaptive bandwidth Gaussian kernel as previously described (*69*). Grouping highly similar cells within small neighborhoods on the SNN graph should then produce a representative sampling of cell-states, provided that the resulting metacells (1) are reasonably distinct from one another and (2) sample all regions of the graph to capture the full extent of heterogeneity. To encode this intuition, metacells are initialized with cells that occupy an "extreme" position on the manifold according to their leverage, representing geodesic distance to all other cells in the graph (*96*).

We first compute leverage for each cell *i*:

$$leverage_i = \sum_l |v_{l,i}|^2$$

where $v_{l,i}$ is the ith element of the lth eigenvector of the normalized SNN graph. Representative cells (metacell "centroids") are then initialized by randomly sampling cells in SNN graph space in proportion to their leverage using the following sampling procedure for each cell *i*:

$$P(centroid_i) \sim Bernoulli(\frac{log(r)}{\varepsilon^2} \times leverage_i),$$

where *r* is a user-defined rank parameter scaling the Bernoulli probability parameter (and therefore controlling the number of centroids selected) and ε is an error tolerance parameter.

Unselected (non-centroid) cells are then assigned to the nearest centroid to form groupings of highly similar cells, after which the centroid is updated iteratively by picking a new cell that minimizes the distance to cells of that group. On each iteration, metacells consisting of fewer cells than a user-defined threshold are pruned to limit sparsity of the resulting profiles. This procedure is continued until no centroids are updated between iterations or a maximum number of iterations is reached; the result is that every cell is assigned to a distinct metacell. Expression or accessibility features are then computed for each metacell by averaging across constituent cells.

Identifying and integrating pancreas metacells

To identify metacells in scATAC-seq data, we applied the above algorithm using the 30 LSI components from ArchR as features for SNN graph construction. For metacell computation, we used k = 30 nearest neighbors, a rank of 200, and minimum metacell size of 10 cells to capture heterogeneity within rare cell populations. This resulted in 264 epigenomic metacells (median 52 cells per metacell, with 75% composed of greater than 37 cells). Each metacell was mainly composed of individual cells from the same PhenoGraph clusters (average entropy in the distribution of cells per cluster in each metacell = 0.067), suggesting that each metacell groups relatively homogenous cells as intended. This enabled us to label each metacell according to the cluster assigned to the majority of its constituent cells (corresponding metacell annotations displayed in **Fig. S6A**).

For scRNA-seq metacells, we pooled a subset of data from comparable conditions (K1–K3, K5, K6; **Table S13**), excluding one tumor for which we did not collect scATAC-seq to avoid confounding intra- and inter-tumor heterogeneity. For one case in which the primary tumor sampled few transcriptomes (n = 261), we included both the primary tumor and metastases—despite lack of scATAC-seq data for metastases—in order to capture enough malignant cells for robust data integration. We applied our metacell algorithm on 49 selected PCs (chosen using the knee point method; **Table S13**) from the log-normalized scRNA-seq data subset (processed as in "Single-cell RNA-seq data processing" for Progression Cohort). Parameters consistent with epigenomic metacell computation (k = 30, rank = 200, 10 minimum cells per metacell) were used to derive a comparable set of 230 transcriptomic metacells, with median 64 cells per metacell and 75% composed of greater than 41 cells. As with epigenomic metacells, each transcriptomic metacell was mainly composed of individual cells from similar PhenoGraph clusters (average entropy = 0.0753).

Next, we constructed a common feature space to match epigenomic to transcriptomic metacells. We treated ArchR gene scores as a proxy for expression, assuming that, on average, cells with high expression for a given gene will also have relatively high accessibility in the vicinity of that gene (and vice versa for low expression). We first averaged gene expression over cells in each transcriptomic metacell, and averaged ArchR gene scores over cells in each epigenomic metacell, to obtain complementary matrices. Next, we z-scored every gene separately within each data modality to derive a standardized metacell matrix for each that was of comparable scale. Finally, we computed a PCA embedding on the standardized, concatenated matrix containing both epigenomic and transcriptomic metacells, to produce a common reduced-dimension space for downstream computation.

To pair RNA and ATAC metacells, we computed a Mutually Nearest Neighbor (MNN) graph across data modalities with k = 30. Magnitude differences between modalities may impact comparison, even with z-scored data; we thus used cosine similarity as a distance metric for nearest neighbor computation to de-emphasize such differences. Moreover, cosine similarity captures an element of gene-gene relationships which is better conserved across data modalities (*64*). Given these mutually nearest pairings, we sought a common visualization across modalities that preserves the general structure of the phenotypic space and reflects both cross-modality and intra-modality cell-state similarities. To achieve this, we first created a metacell-by-metacell combined graph with dimension $N = N_t + N_e$ where N_t and N_e represent the number of transcriptomic and epigenomic metacells, respectively. We defined edges between metacell nodes as a weighted sum of cross-modality MNN edge weights and intra-modality nearest neighbor (NN) graph (also computed on cosine distance) weights:

$$G = \alpha \, G_{MNN} + (1 - \alpha) \, G_{NN},$$

where α is a parameter that defines the trade-off between cross-modality and intra-modality cell-state similarity. We set $\alpha = 0.4$ to slightly favor the phenotypic landscape and projected the data as an FDL on G (**Fig. S6A**). In this layout, we found that metacells group by their predefined clusters, and transcriptomic and epigenomic metacells belonging to the same cell-state tend to co-cluster. Indeed, standardized accessibility and expression show strong concordance for key marker genes (*Ptf1a, Tff2, Nes*, and *Neurod1*) in this visualization (**Fig. S6B**).

We proceeded to further validate the agreement between accessibility and expression for our MNN pairs. For each transcriptomic metacell, we computed a corresponding average accessibility profile across its epigenomic MNN pairs by first averaging the non-standardized

ATAC signal across neighbors of each RNA metacell, then z-scoring the averaged value. We then inspected the per-gene Pearson correlation of expression and aggregated accessibility, finding that the majority of genes have strong positive correlations, particularly cell-state markers such as *Nr5a2, Krt19, Tff2,* and *Nes* (**Fig. S6C**). This demonstrates the consistency in our computationally derived pairings between transcriptomic and epigenomic datasets.

Measuring epigenetic plasticity

With MNN analysis, we matched transcriptomic and epigenomic metacells based on the most similar states. However, various degrees of gene priming—open chromatin in unexpressed regions—should lead to mismatched chromatin and expression features for a subset of genes. We therefore asked whether epigenomic metacells might be similar to additional transcriptional states beyond their MNN pairs. We computed pairwise correlations on expression HVGs between each transcriptomic and epigenomic metacell. In particular, for each vector of ArchR gene accessibility scores in one epigenomic metacell, we computed Pearson correlation to the vector of gene expression values in one transcriptomic metacell across all genes captured in both modalities. This revealed many cases in which metacells from the two modalities were highly correlated despite representing disparate cell-states (**Fig. 3C**).

These relationships may be explained by lack of cell-state separation within a single data modality (for example, cells undergoing continuous lineage transitions may be highly transcriptionally related to neighboring phenotypes immediately up-stream or down-stream along their lineage), or by a true disparity between cells' epigenomic and transcriptomic features. To

control for the former possibility, we repeated this analysis within each modality separately, correlating each pair of transcriptomic profiles or epigenomic profiles. We observed extremely strong on-diagonal correlations in these cases corresponding to identical cell-states, as well as weaker off-diagonal correlations (**Fig. S6D**).

We thus sought to quantify the extent of similarity between disparate cell-states in each comparison (ATAC vs. ATAC, RNA vs. RNA, and ATAC vs. RNA). For each case, we computed the average r_intra, which we define as the average pairwise correlation of all pairs of metacells annotated to the same cell-state, and r_inter, which instead measures the average pairwise correlation of disparate cell-states. The difference between these two metrics (r_intra - r_inter) describes the relative correspondence between identical cell-states versus disparate cell-states within that comparison. We found that intra-cluster similarity is much greater for comparisons within one technology (rintra - rinter = 0.51 for RNA and 0.42 for ATAC) compared to cross-modality similarity (rintra - rinter = 0.26); hence, epigenomic metacells with similarity to multiple transcriptional states represent examples of a mismatch between epigenomic and transcriptomic features which cannot be fully explained by lack of cell-state separation within a single data modality. We hypothesize that these may encode multipotential states, which we define to be a cell's epigenetic plasticity.

Quantifying epigenetic plasticity*:* We assume that (1) plastic cell-states have access to diverse transcriptional programs that drive distinct cell phenotypes, and that (2) a common mechanism for providing access is epigenetic priming, or opening the proximal chromatin of fate-associated genes prior to receiving fate-specifying signals. To quantify this plasticity, we use a classification-based approach to detect cell-type-specific gene expression encoded at the

chromatin level (see similar approach in "Predictive model of late-stage fates"). Importantly, we choose here to employ classification over the correlation-based analysis above for several reasons. For one, classification methods are adept at learning relevant features (gene expression programs) to discriminate classes (cell-states). Such approaches can extrapolate knowledge learned from training data to held-out datasets, or in our case, from one data modality (gene expression) to a second (chromatin accessibility). Second, certain classifiers (for example, regularized classifiers) can detect the specific features that are most predictive at defining classes. This is as opposed to correlation, which treats all features equally and therefore may be impacted by less relevant features (for example, housekeeping genes). We thus reasoned that a classifier trained to learn cell-state-specific features in transcriptomic data could be used to predict expression phenotypes from chromatin-derived features. Furthermore, we posit that uncertainty in such predictions is a measure of plasticity; epigenomic states that map to a wide range of transcriptional cell types have the greatest potential to express diverse phenotypic programs. This concept is summarized in **Fig. 3D**.

We first defined discrete expression phenotypes using our refined PhenoGraph scRNA-seq clusters to capture a finer granularity of transcriptional states, particularly those separating injured and uninjured states (**Fig. S1C**, see "Single-cell RNA-seq data processing" for details). To ensure robust classification, we discarded rare clusters (<200 cells) corresponding to 6 metacells; all remaining clusters had reasonable amounts of data for training. Using this filtered dataset, we trained a multiclass logistic regression classifier with sklearn on transcriptomic metacells to predict cell-state label (PhenoGraph cluster of the metacell center) from aggregated, standardized gene expression features for that metacell. We trained the model on a subset of 60% of transcriptomic metacells (N = 138) and achieved strong predictive performance on a held-out

validation set (accuracy = 0.9457, averaged precision = 0.9489, averaged recall = 0.9444), indicating that the model can successfully identify phenotype-specific features in gene expression.

Our classifier provides a function *f* which takes as input an expression program of metacell *i* (with dimension *D* = number of genes) and outputs a probability distribution over cellular state (with dimension = number of transcriptomic states), where a class label is given as:

$$c_i = argmax_{1 \dots K_t} \, y_i$$

We next asked, for each cell-state, whether using accessibility features as a proxy for expression results in clear mapping to the expected cell-state, or an uncertain mapping to more than one cell-state, suggesting chromatin primed for diverse gene programs. Specifically, we applied our logistic regression model (trained on transcriptomic metacells) to classify epigenomic metacells by using accessibility features for *x*, thereby assigning a most-probable PhenoGraph cluster class (initially identified from expression data) to each epigenomic metacell.

To summarize the predictions, we counted the number of epigenomic metacells from each epigenomic cluster that classified to each transcriptomic cluster. For the set of epigenomic metacells belonging to epigenomic cluster k_e, we computed the number of metacells which classify to transcriptomic cluster k_t as follows:

$$M(k_e, k_t) = \sum_{i \in \{k_e\}} I(c_i = k_t) \, ,$$

where I is the indicator function, and c_i is the class label, as described above. These values define a "confusion matrix" with dimension K_e (number of epigenomic cell-states) by K_t

(number of transcriptomic cell-states), where the element $M(k_e, k_t)$ quantifies the extent to which each epigenomic state (rows) classify to each transcriptome state (columns). We then examined this confusion matrix, standardizing each row to emphasize the tendency of each distinct chromatin profile toward various expression states (**Fig. 3E**). Highly plastic states can thus be identified as epigenomic clusters (rows) containing metacells that map to multiple transcriptomic cell-states (columns), particularly from disparate cell types. Besides this approach, which captures only the most likely transcriptomic class per epigenomic metacell, we further summarize these cross-modality mappings as the average log probability per epigenomic state (row) toward each transcriptomic state (column) in **Fig. S6E**. This emphasizes plasticity in certain states whose probability distribution is peaked near their correct class (for example, tuft cells), but have slight probabilities toward other classes. This is in contrast to acinar-like ADM cells, which consistently classify to ADM states and rarely have any substantial probability of classifying to another state. The distributional representation of **Fig. S6E** is thus well-suited for comparison to the correlation-based analysis in **Fig. 3C**, as it displays each cell-state's tendency across all other states (and not just its most optimal state).

Calculation of plasticity score*:* To enable analysis beyond cluster-level metrics, we quantified this observed plasticity on a per-cell basis by computing Shannon entropy of the probability distribution *y* across classes from the classifier predictions:

$$Plasticity = -\sum_{k_t=1}^{K_t} P(y_{k_t}) \log P(y_{k_t}),$$

for each transcriptomic state *k* = 1...K. This score will be high when the probability is widely distributed across many transcriptomic classes, hence a metacell's epigenome displays accessibility for diverse gene expression programs. Conversely, it will be low when the

distribution peaks at a single cell-state, indicating that a metacell's epigenome is strongly linked with its own expression readout.

Measurement of plasticity on a numerical scale enables us to address questions about which factors (such as gene programs, stage in progression) influence plasticity. In **Fig. 3F**, we visualize its distribution across cell-states to identify the states which display high epigenetic plasticity. Furthermore, we find that plasticity is enhanced upon inflammation by comparing distributions of plasticity scores per metacell in *Kras*-mutant cells (K1) versus those responding to inflammation (K2) (**Fig. 3H**).

Robustness of plasticity approach: To test whether our plasticity metric is robust to cell-states captured (rare populations), we randomly subsampled transcriptomic metacells 100 times, selecting 75% of profiles with replacement for individual trials. Each trial measures whether our discriminative model of cell-state is consistent with respect to observed transcriptomic profiles. Indeed, we found high concordance between plasticity scores derived from each trial and the true scores, with average Pearson correlation coefficient of $r = 0.895$ (minimum $r = 0.703$) between subsampled and true scores for each epigenomic metacell. Subsampling transcriptomic metacells to 50% in this procedure maintained strong concordance (average $r = 0.8565$, minimum $r = 0.6177$).

Annotation of plasticity score with GSEA: Beyond identifying high-plasticity populations, our metric serves as a powerful quantitative tool to explore features of plastic cells. In particular, by ranking metacell profiles by plasticity score, we can correlate gene programs with epigenetic plasticity. To this end, we computed Spearman correlations between each gene's accessibility score and the corresponding plasticity score per cell (**Table S5**), and used these values to rank

genes for Gene Set Enrichment Analysis (GSEA) (*48*). GSEA was performed with the gseapy python package using all genesets included in the "KEGG_2019_Mouse" library (*97*), with an FDR of 0.1 (**Figs. 3G** and **S6F**, and **Table S4**).

Heterotypic cell-cell communication analysis with Calligraphy

Our results collectively suggest that inflammation is associated with plasticity in pre-neoplasia, prompting us to further explore potential interactions with the immune microenvironment in pre-malignancy. Many approaches exist to infer cell-cell interactions via expression of cognate receptor-ligand (R-L) pairs across cell types, defining significant interactions with respect to single pairs of R-L genes against a null model of R-L expression (*50*). However, their sensitivity and specificity is impacted by the limited capture rate for individual genes (particularly stable cell-surface proteins), as well as weak support for each of many potential interaction pairs. Some methods address weak or noisy signal by incorporating gene expression downstream of signaling, but these approaches derive gene pathways from general databases without regard to cell-type-specific mechanisms (*50*). This has the disadvantage of ignoring context-specific, cell-intrinsic signaling, including the many pathways impacted by oncogene activation that are relevant to our setting.

In this study, both bulk and single-cell epigenetic data revealed considerable remodeling of chromatin structure proximal to communication genes (such as those encoding for inflammatory receptor and ligand proteins). Given this pervasive remodeling of a large number of communication genes and the modular nature of gene regulation, we hypothesize that the

remodeling of communication genes is also organized into coregulated modules, which can be exploited to strengthen the detection of true signal in the data. Indeed, expression-based clustering of communication genes exhibits striking modularity (**Fig. 4A**). We therefore designed a new approach to crosstalk inference, Calligraphy, which is context-specific and uses gene coregulation to improve the sensitivity and robustness of inferred interactions.

Calligraphy is rooted in ′modules′ of inflammation-associated genes: each cell can receive signals based on its expressed receptors and send signals based on its expressed ligands. Specifically, modules are sets of communication genes that tend to be mutually expressed in the same populations, and hence summarize the possible incoming and outgoing communication for a particular state. To identify these patterns, Calligraphy builds a co-expression network of communication genes, from which robust inferences can be made across sub-populations representing coherent inflammatory programs. Prior knowledge of potential R-L relationships informs the communication potential across cell-states based on their relative module usage, drawing from data on the numerous communication genes in each module to predict possible interactions. Below, we describe how we infer communication modules de novo from scRNA-seq data, then apply a module-based approach to map potential crosstalk in the pre-malignant pancreas.

Co-expressed communication module detection: We first mapped communication gene co-expression patterns across the pre-malignant landscape. To account for gene drop-out, we imputed gene expression using MAGIC (*69*), which has been shown to increase power to detect co-expression trends in single cell data. Specifically, we applied MAGIC to the log-normalized count matrix of pre-malignant K1–K3 stages, which generates a cell-cell affinity graph using

defaults k = 5 and t = 3. The t parameter was chosen to only produce modest smoothing along the manifold and avoid over-inflating the expression of true negative genes.

We visualized the MAGIC-imputed gene-gene correlation matrix across all annotated communication genes to expose potential modules, with rows and columns ordered according to hierarchical clustering using centroid (UPGMC) linkage in sklearn (*94*) (**Fig. 4A**). The matrix exhibits a striking degree of structure; blocks of mutually correlated communication genes representing inflammatory modules within the oncogenic epithelia are readily apparent. We also observed substantial variability in correlation magnitudes outside of coherent blocks, suggesting the need for a module detection approach that is robust to spurious correlations induced by imputation and noisy data. Furthermore, large blocks of off-diagonal correlation implied substantial sharing of specific communication genes across programs and/or cell-states. This finding highlights an important biological feature of signaling: many individual communication genes are expected to participate in the communication of multiple cell-states, including immune subsets and epithelial states. This motivated a second criterion for our module-based crosstalk approach: flexibility with respect to sharing of receptors or ligands across distinct communication modules.

Our module detection approach with Calligraphy thus begins with thresholding the gene-gene correlation matrix (Pearson correlation coefficient >= 0.4) to derive a graph, in which genes are nodes and correlation-weighted edges connect highly correlated genes. We stored and computed all graph information using the networkx python package for efficient network handling (*98*). To improve module coherence and remove spurious associations, we compute a Jaccard similarity

metric accounting for the degree of neighbor-sharing between all pairs of genes, similar to (*85*), as follows:

$$Jaccard\ (Gene_i, Gene_j) \ = \frac{N_i \cap \ N_j}{N_i \cup \ N_j}$$

where N_i is the set of neighboring genes for gene *i*. We define an upper and lower threshold for this Jaccard metric, retaining existing edges which meet the lower threshold and appending new edges which meet the upper threshold. This step ensures mutual neighbor sharing and thus removes edges which are likely due to spurious correlations.

To group genes according to this graph, typical community detection approaches may target high graph modularity, requiring communities to represent non-overlapping sets of nodes (*85*) which is not ideal for our setting. To allow gene sharing between modules, we applied the Order Statistics Local Optimization Method (OSLOM) algorithm (*99*) for overlapping community detection on the Jaccard-neighbor graph. Briefly, the method clusters an undirected graph by optimizing a measure of cluster significance against a random null network lacking community structure. We applied the entire module-detection approach separately to the epithelial pre-malignant cells and matched samples from the immune data split into (1) T cell, NK cell and innate lymphoid cell (ILC), (2) myeloid cell, and (3) B cell subsets (**Fig. S10B,C** and **Table S15**). These groupings allowed the discovery of more refined modules which correspond with co-expression patterns in rarer immune subsets, including Tregs and plasma cells. Hence, we obtained four sets of communication modules which we term epithelial, T/NK/ILC, myeloid, and B cell sets. Finally, we inspected each module to assess the biological plausibility based on knowledge of cell-state programs. As we occasionally observed nonsensical, lowly expressed

genes in otherwise coherent modules (for example, Natural Killer lectin -like receptors in epithelial modules), we added a filtering step to remove genes expressed in <10 cells in each subset. Performing this filtering post-module inference provides an interpretability benefit, as we were able to assess its impact on module plausibility guided by biological knowledge of gene groupings.

Association of modules with cell-state: To utilize our communication modules for cell-cell signaling inference, we sought to associate cell-states with their expressed modules. As an initial step, we devised a strategy to quantify and visualize relative module expression per cell (**Fig. 4B**). We first defined a color-coding matrix of dimension M x 3, where M is the number of modules, and each row contains a user-defined, 3-dimensional RGB code that specifies a distinct color for its corresponding module. We next assigned a single RGB color to each cell, based on its module usage, as a linear combination of module-specific colors, akin to "pseudo-coloring" used for microscopy images. Specifically, the matrix product of the color-coding matrix with an M x N matrix containing the average module expression in each cell (scaled between 0 and 1) gives a pseudo-coloring of each cell by its relative module expression. Module expression per cell is computed as the log of average normalized expression of each gene in the module. Cells with low expression for all modules will be close to black (R = 0, G = 0, B = 0); cells strongly skewed toward one module will assume a color value similar to that module's code; and cells that highly express two or more modules will assume a hue that is intermediate between module colors. Hence, the predominantly monochrome, module-specific color distribution in the FDL visualization of pre-malignant cells (**Fig. 4B**) implies a strong degree of mutual exclusivity in communication module expression across the epithelia.

The partition of module activity across epithelia suggested that it would be reasonable to annotate each epithelial communication module based on its prevalence within a cell-state. We generated hard assignments for each cell based on the communication module with highest average expression (z-scored across cells for comparable scale), and confirmed that a visualization of the assignments mirrored the module pseudo-coloring (**Fig. S7C**). Checking the extent to which these assignments intersected cell-state definitions computed using PhenoGraph on the full dataset (see Progression Cohort coarse clustering in "Single-cell RNA-seq data processing"), we found that cells of sizable coarse clusters (>200 cells) were largely assigned to a single module, with 62% of cells in each cluster, on average, assigned to the predominant communication module (**Fig. S7D**). Further, the Rand index between clustering and module assignment was relatively strong at 0.35. Given this degree of pairing between cell-states and communication modules, we were able to annotate each module based on the most closely associated cell-state (which cluster the cells associated with a module most frequently originate from; see **Fig. S10C** for immune module visualization). One epithelial communication module had low expression across all major populations, and was not annotated. We repeated this process of module annotation separately on the three major immune subsets (**Fig. S10B,C**).

We hypothesized that the extreme degree of overlap between communication gene modules and cell-states is a special feature of communication genes, and hence would not be reproducible for modules determined on any other set of genes. We thus tested the ability of randomly selected genes to recapitulate the degree of structure observed for receptors and ligands. We began by binning genes into 40 groups based on their average expression across cells, so as to not bias our analysis based on highly- or lowly- expressed genes. To produce a control set, we randomly select a gene from each communication gene's expression-matched bin. We then apply the

OSLOM algorithm as described above (with the same parameters) to derive a set of modules from these non-communication genes. To evaluate matching to cell-state, we compute the rand index between cells' predominant module assignment and coarse PhenoGraph clusters. This was repeated for 200 trials, each time down-sampling genes (dropping 10% each trial) for module computation. Finally, to derive a comparable distribution for communication genes, we randomly sampled an equivalent number of communication genes and repeated rand index computation as above.

Unsurprisingly, we do find that randomly selected genes display a moderate amount of structure across our populations; indeed, gene expression across the entire transcriptome is expected to be highly modular due to tightly controlled regulatory programs, and for this reason we find that modules derived from random genes do organize into population-associated groups. It is also important to note that our module detection algorithm will discard genes with low structure, thereby automatically selecting for genes with a degree of modularity and discarding those without (for example, housekeeping genes). Still, we find that communication modules are consistently more modular with respect to our cell-states compared with random samplings of genes (t-test, p value < 0.01) (**Fig. S7E**).

Comparison of pre-malignant epithelial modules to normal pancreas and cancer: The correspondence between our modules and cell-states suggests that communication module expression can largely explain transcriptional heterogeneity in the pre-malignant epithelia. We asked whether this also holds in normal pancreas, normal regeneration and late-stage malignant disease. To this end, we computed average pre-malignant communication module expression in N1-N2 cells and K5-K6 cells, and visualized module distribution using the pseudo-coloring

approach above (**Figs. 4C,D**). The majority of normal regeneration (N1, N2) cells exhibited low expression across all communication modules (dark pseudo-coloring), suggesting that pre-malignant inflammatory modules are largely inactive outside the oncogenic context. In contrast, malignant cells (K5, K6) expressed high levels of Progenitor, Gastric, and Bridge communication modules.

We additionally integrated our mouse-derived communication modules with publicly available human data (*32*) processed as described above. For each module defined in the pre-malignant pancreas, we computed the log of the average expression of each homologous gene across all human epithelial cells. **Fig. 4E** displays distributions of these scores across cells per module. This reveals that Progenitor, Gastric, and Bridge modules are up-regulated in human PDAC cells, consistent with the notion that these modules persist in advanced murine tumors.

Gene-centric module crosstalk algorithm*:* The communication modules define inflammatory programs that are differentially expressed across cell-states and are largely specific to the pre-malignant context. We next sought to identify module crosstalk that represents heterotypic communication driven by tissue inflammation in the context of oncogene activation. We first filtered the module graph described above, removing nodes which are not differential between mutant *Kras*-associated injury (K2) and regeneration (N2). Specifically, we identified genes upregulated in K2 compared to N2 from bulk RNA-seq data published in (*23*), retaining those with DESeq logFC > 2 and with adjusted p value < 0.05. We also retained nodes which are cognate pairs of these dynamic genes, to capture potential cell-cell interactions and downstream effects of these differential programs. In total, 55 receptors and 46 ligands remained out of 340 initial candidates. For all crosstalk analysis, R-L cognate gene pairs were extracted from (*100*)

and were manually curated to include additional PDAC and immune-relevant R-L pairs not included in the initial list (**Table S18**).

Next, we modified the graph to include directed edges between ligands and their cognate receptors (rather than edges between co-expressed genes), representing potentially interacting molecules involved in cell-cell communication between modules. Similar to previous methods which identify crosstalk events between cell-states, in this gene-centric approach, we consider two modules to be potentially interacting if they share many cognate R-L pairs. As such, we enumerate the number of cognate interactions that occur between all genes in each pair of interacting modules. To identify only module pairs whose counts are higher than chance, we compute a random null distribution R on the pairwise interaction counts by shuffling the module labels for each gene and re-computing the counts for n = 5000 trials. We then compute empirical p values for each interacting pair as 1 - p(R < observed) to identify significant interactions, akin to the procedure in CellPhoneDB (*50*) (**Fig. S11A**). Resulting networks are visualized in **Figs. S11B**,**C**, where each node represents one module and weighted edges represent statistically significant module-module interactions inferred by Calligraphy.

Per-module sender and receiver score: Our crosstalk inference approach provided a comprehensive map of candidate cell-cell interactions across communication modules and their associated cell-states. We visualized these interactions as a graph with modules as nodes and edge weights indicating strength of interaction (number of edges between modules) (**Figs. S11B,C**). We reasoned that modules with numerous interactions across epithelial and immune subtypes represent central ('hub') communicators of injury-driven neoplasia, and sought to quantify this notion. For each module, we summed counts of outgoing and incoming edges in the

full gene-gene interaction network (filtered for statistically significant interactions, p value < 0.1) to quantify a "sensing" and "re-modeling" score, respectively. Visualization of these scores as bar charts along a heatmap of pairwise significant module-module interactions highlights modules with high sending and/or receiving propensity, excluding modules with no incoming or outgoing edges from columns and rows respectively (**Fig. 4F**).

Analysis of sequential paths through crosstalk network: Sensing and re-modeling scores annotate important communication modules with substantial pairwise interactions between cell-states. However, many consequential intercellular events may involve stepwise interaction between more than two epithelial or immune populations. For instance, *feedback loops*, wherein a module signals back to itself through one or more intermediate populations, have been identified in late-stage cancer (*58*). To identify putative feedback loops in our Calligraphy network, we performed a search for cycles using networkx's simple_cycles function. This identified a single loop involving Gastric module (E6) and Treg/ILC module (T8).

A major goal toward translating cell-cell communication networks to actionable targets is the identification of molecules whose expression has widespread impacts on downstream targets. To determine the relative impact of any one ligand within our feedback loop, we computed hierarchical paths beginning with a single "source" ligand, downstream to its immediate binding partners, and from each of these sequentially through all possible paths in the Calligraphy network. For computational efficiency, we began this search by identifying "sink" modules in the network which have no significant outgoing edges and hence represent dead-end absorbing states in a walk on the graph from any origin. For a particular source ligand contained in a particular module, we identified all its receiving modules containing its cognate binding partners

as inferred by Calligraphy. We then rapidly built the downstream hierarchy of all possible paths emanating from these downstream modules by applying networkx's all_simple_paths function to enumerate paths of all possible lengths to each sink. We visualized paths using plotly's sunburst function, annotating the inner circle with source ligand and each outward layer as a possible step along the path hierarchy (**Fig. 5E**).

To then determine the impact of the source ligand on any given module, we annotated each downstream module by the level of its earliest appearance in the hierarchy. Assuming that communications occurring with fewer intermediates are most likely or most potent, these scores signify the putative impact of the source ligand (with lower scores suggesting stronger impact). To then associate these module impact scores to cell-states, we annotate each cell by its most highly expressed module as described above (see "Association of modules with cell state"). Transfer of module impact scores to their associated cells allowed us to assess the breadth of phenotypes which may be affected by expression of a given ligand (**Fig. 5F**).

Comparison to CellPhoneDB: We sought to determine whether the above findings could be recapitulated by a commonly used algorithm for communication inference in scRNA-seq data. CellPhoneDB (*50*) is similar to Calligraphy in its use of receptor-ligand expression patterns for detecting such interactions from dissociated data, even proposing a highly-similar approach for establishment of a null distribution based on permutation. On the other hand, as CellPhoneDB does not leverage co-expression patterns, it applies a separate statistical test for each receptor-ligand pair. It also requires fixed cell-state definitions (clusters) to compare the expression of each receptor or ligand across heterogeneous states.

To compare the performance of CellPhoneDB to Calligraphy, we first began by defining cell-states in our data using coarse PhenoGraph clusters as described above. We ran CellPhoneDB on our scRNA-seq data using method "statistical_analysis" with otherwise default parameters. To understand its output, we first looked for potential interactions by thresholding all corrected p values to an FDR of 0.01. Across all pairs of cell-states, we found that the vast majority (720/729, or ~98%) had at least one significant interaction. For comparison, if we map Calligraphy modules to their predominant cell-states (based on average expression), we get 41 such interactions passing significance (a mere 5.6% of possible interactions).

Single-cell analysis of KC-shRNA cohorts

Analysis of ligand-specific paths in our crosstalk network revealed an IL-33-driven feedback loop involving pre-malignant populations and their microenvironment. To determine the impact of this crosstalk on epithelial cells, we modeled phenotypic shifts in each stage and tissue type of our Perturbation Cohort, comparing sh*Il33* to control samples with the Milo algorithm (*60*). Milo groups cells into partially overlapping local neighborhoods on a kNN graph, and then computes statistics for differential neighborhood abundance across conditions using a negative binomial generalized linear model (GLM). We applied Milo with k = 20 for neighborhood detection and took advantage of its GLM to further account for batch confounding by modeling the experimental mouse cohort as a covariate. We visualized the distribution of log fold changes in each analysis (K2 epithelial and immune, and K3 epithelial and immune) separately as a measure of degree of perturbation in each condition (**Fig. 6C**).

K3 epithelium displayed the strongest and most consistent phenotype for further exploration of cell-states impacted by perturbation. We were primarily interested in understanding whether our perturbation alters the natural progression of disease. To model an axis of progression in this time point, we applied pseudotime inference with Palantir (*61*) to neighborhoods derived from Milo. Palantir requires assignment of a starting cell in order to seed the direction of pseudotime. In our case, we chose to use *Nes*$^+$ progenitor cells as the primary cell of origin, supported by CellRank's identification of this state as an initiating population (**Fig. S3B**) as well as multiple previously described measures suggesting the potential of this population to give rise to downstream cell states (**Figs. S4D** and **3F**).

To identify a *Nes*$^+$ progenitor neighborhood which could serve as a starting cell for pseudotime, we first annotated each neighborhood in Milo using the annotateNhoods function with cell-state annotations assigned to each original single cell as described above for the Progression Cohort (see "Single-cell RNA-seq data processing and basic analysis" for list of marker genes) (**Fig. S13E**). We then computed diffusion maps with Palantir (n_components=10) to identify major axes of variation through the neighborhoods. We selected a starting cell at the extreme of the second DC, which tracked from a *Nes*$^+$ progenitor to downstream cell types. Palantir was run with 500 waypoints to identify an axis of pseudotime from this starting position (**Fig. S13E**). In **Fig. 6G**, we visualize the Milo differential abundance with neighborhoods sorted along this axis along with a 6th-order polynomial regression line fit to the trend of logFC, finding increased abundance in sh*Il33* for the latest subset of *Nes*$^+$ progenitors. We further find that this population maintains expression of genes highly correlated with the plasticity score across epigenomic metacells (Pearson correlation Bonferroni corrected p value < 0.01) based on average, z-scored expression of these genes.

Finally, to interpret these results in light of our inferred module interaction networks, we evaluated the extent to which cell-states predicted to be downstream of IL-33-centric crosstalk pathways overlap with those impacted by the perturbation. To this end, we divided epithelial modules into 'connected' modules which are directly or indirectly downstream of IL-33 in the network and 'unconnected' modules, which are not. We reasoned that connected modules are more likely to be impacted by perturbation, provided that our module interaction network captures true interactions driven by this cytokine. To utilize neighborhood-specific scores from Milo as a measure of these module-level impacts, we first associated modules with neighborhoods, similar to our approach in "Heterotypic cell-cell communication". Specifically, we computed hard module assignments for each neighborhood based on the module with the highest average z-scored expression (**Fig. S13G**). We then compared the average absolute value Milo log fold change in all neighborhoods assigned to IL-33-connected modules versus unconnected modules, finding significantly higher perturbation impact in connected modules (one-sided t-test; $t = -4.6711$, p value = 2.0551×10^{-6}), and thus experimentally validating connections in the inferred IL-33 crosstalk pathway (**Fig. 6H**).

Figure S1

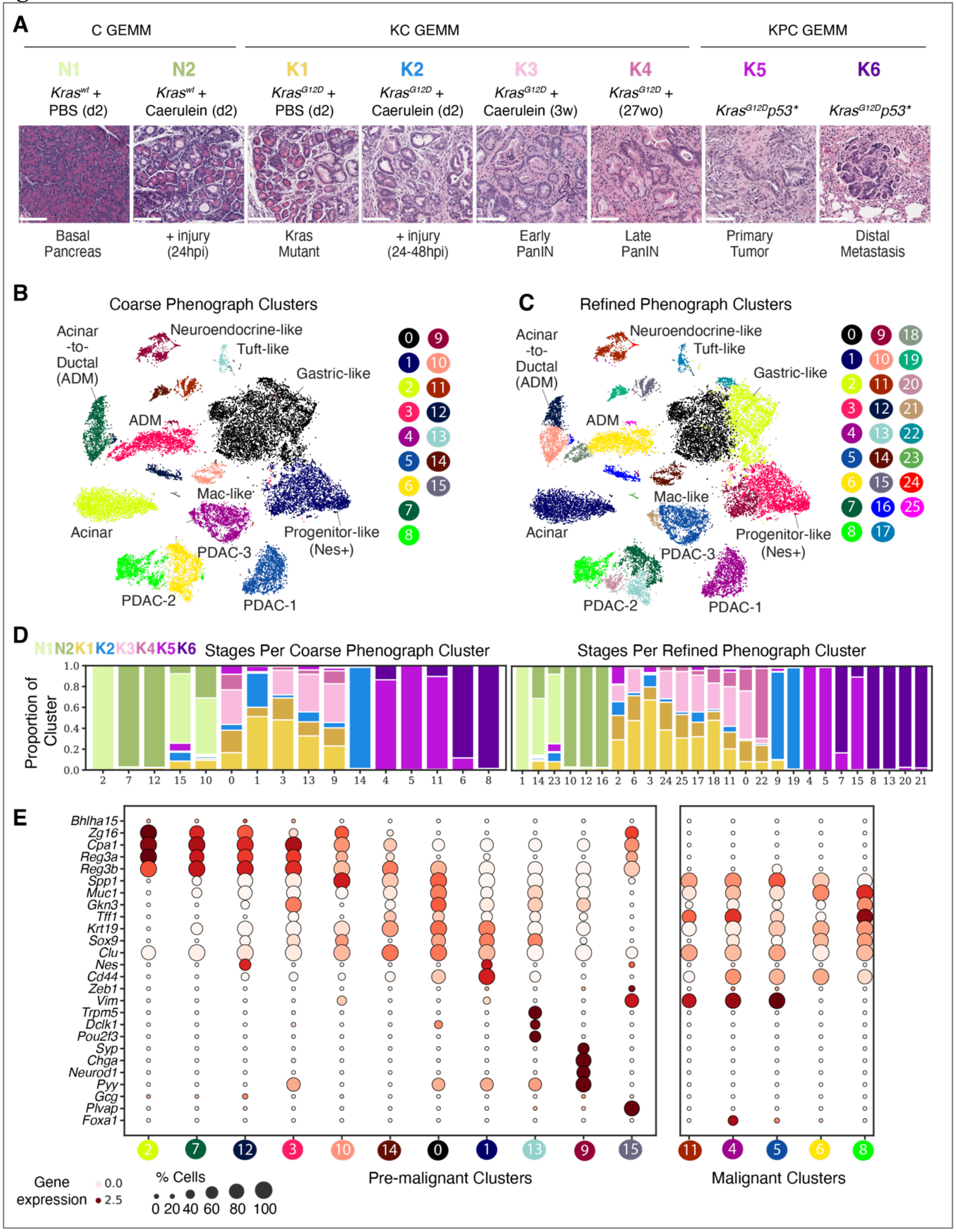

Intra- and inter-tissue epithelial heterogeneity and dynamics during cancer progression. (**A**) Experimental settings to interrogate pancreatic epithelial heterogeneity in vivo. Representative H&E of pancreata from the indicated mouse models and treatment conditions (as in **Fig. 1A**). Scale bar, 100 µm.

(**B**) tSNE visualization of epithelial (mKate2^{+}) scRNA-seq profiles from all collected settings in (A), colored by coarse cluster membership computed with PhenoGraph (*85*); clusters are annotated manually. ADM denotes cells actively undergoing acinar-to-ductal metaplasia in Kras-wild-type or mutant tissue (*31*).
(**C**) tSNE map as in (B), colored by fine cluster membership computed with PhenoGraph.
(**D**) Bar plots showing the proportion of cells from each coarse or refined PhenoGraph cluster obtained from each stage of progression.
(**E**) Expression of pancreatic epithelial cell-state markers (rows) across coarse PhenoGraph clusters (columns). Dot size scales with the proportion of cells in a given cluster that express each gene; color indicates average z-scored, log-normalized expression. Cells in pre-malignant conditions gradually lose expression of acinar-associated markers (*Bhlha15, Zg16, Cpa1*) and gain expression of metaplasia-associated markers (*Krt19, Sox9*). Other populations express distinct cell-state markers including *Syp*, *Neurod1*, and *Pyy* in neuroendocrine-like cells (cluster 9) and *Trpm5*, *Dclk1*, and *Pou2f3* in tuft cells (cluster 13).

Figure S2

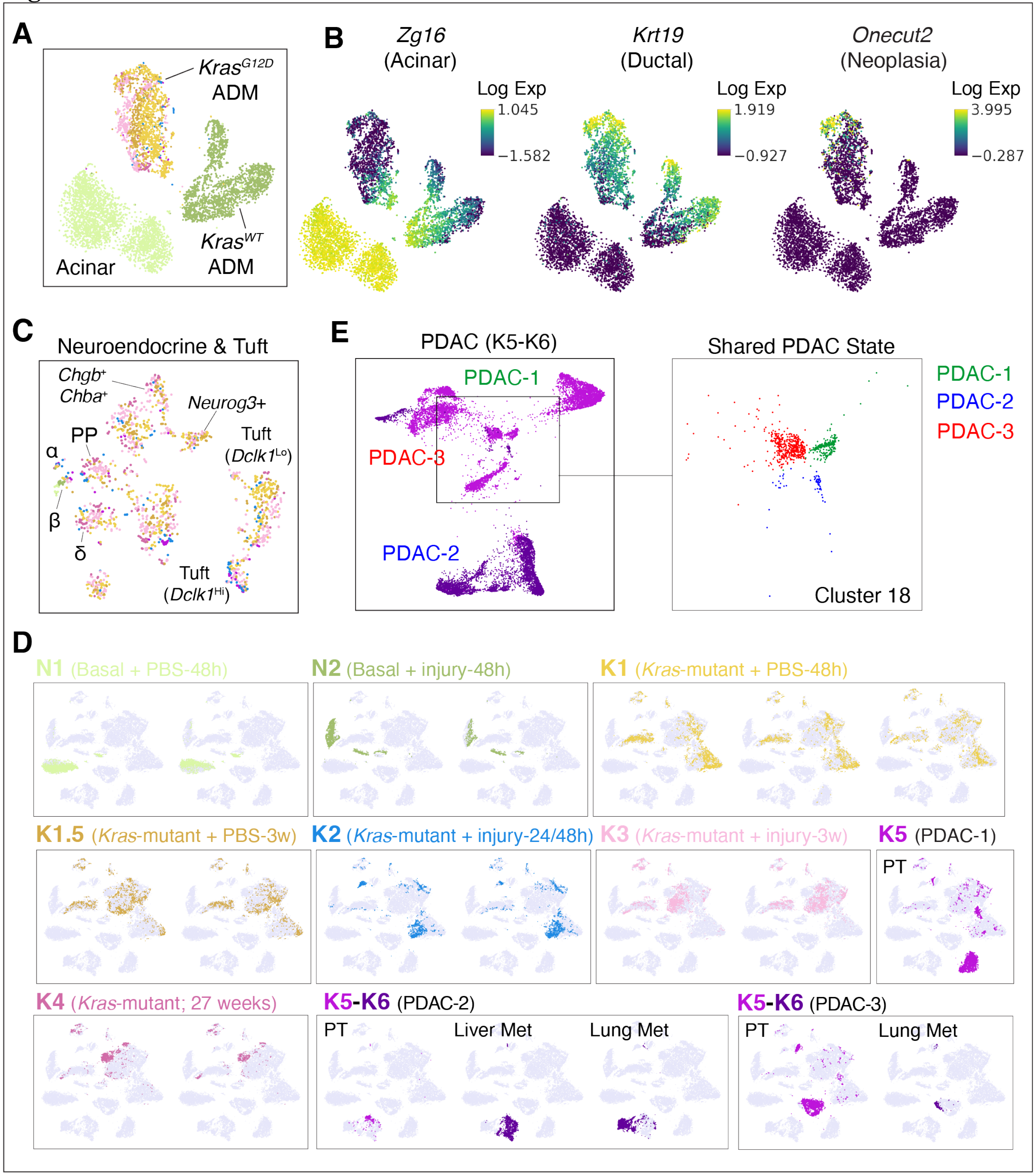

Resolution and reproducibility of pre-malignant epithelial cell states.
(**A**) tSNE map of cells derived from coarse PhenoGraph clusters 2, 3, 7 and 12 (see **Fig. S1B**) undergoing ADM in regeneration (N1, N2) and tumor progression (K1–K6). Normal and oncogenic *Kras* conditions exhibit little overlap.
(**B**) tSNE map as in (A), colored by log-normalized expression of acinar gene *Zg16*, which decreases across both *Kras*-wild-type and *Kras*-mutant ADM; ductal gene *Krt19*, which increases along both; and neoplasia-associated gene *Onecut2* (*52*), which is specifically activated along ADM in the *Kras*-mutant counterpart.

(**C**) tSNE map of neuroendocrine and tuft cells derived from coarse clusters 9 and 13, showing substantial heterogeneity in *Kras*-mutant cells (largely absent from *Kras*-wild-type epithelia, which only contain an extremely rare population of beta -like cells) and rare cell populations captured through epithelial cell enrichment. Substantial mixing of cells across conditions further underscores reproducibility of transcriptomic profiles.
(**D**) tSNE map as in **Fig. 1B**, highlighting biological replicates (independent pancreatic or distal metastases tissue per mice) for each condition (**Table S1)**. Each tSNE represents an individual tissue per mouse, colored to indicate constituent cells. Normal (N1), regenerating (N2) and pre-malignant (K1-K4) pancreatic epithelial cells are highly reproducible across independent individual mice, whereas cells from malignant conditions (K5, K6) diverge. In malignant conditions, primary tumors and metastases derived from a single mouse are grouped together in one box, highlighting both phenotypic overlap in cells derived from the same mouse and metastasis-associated transitions in each.
(**E**) FDL of *Kras*-mutant (mKate2^{+}) cells derived from full-blown PDAC samples (K5, light purple) and isogenic distal metastases (K6, dark purple) highlights phenotypic divergence between individual mice (PDAC-1 to -3), with the exception of a single shared state (cluster 18) from all 3 malignancies (inset).

Figure S3

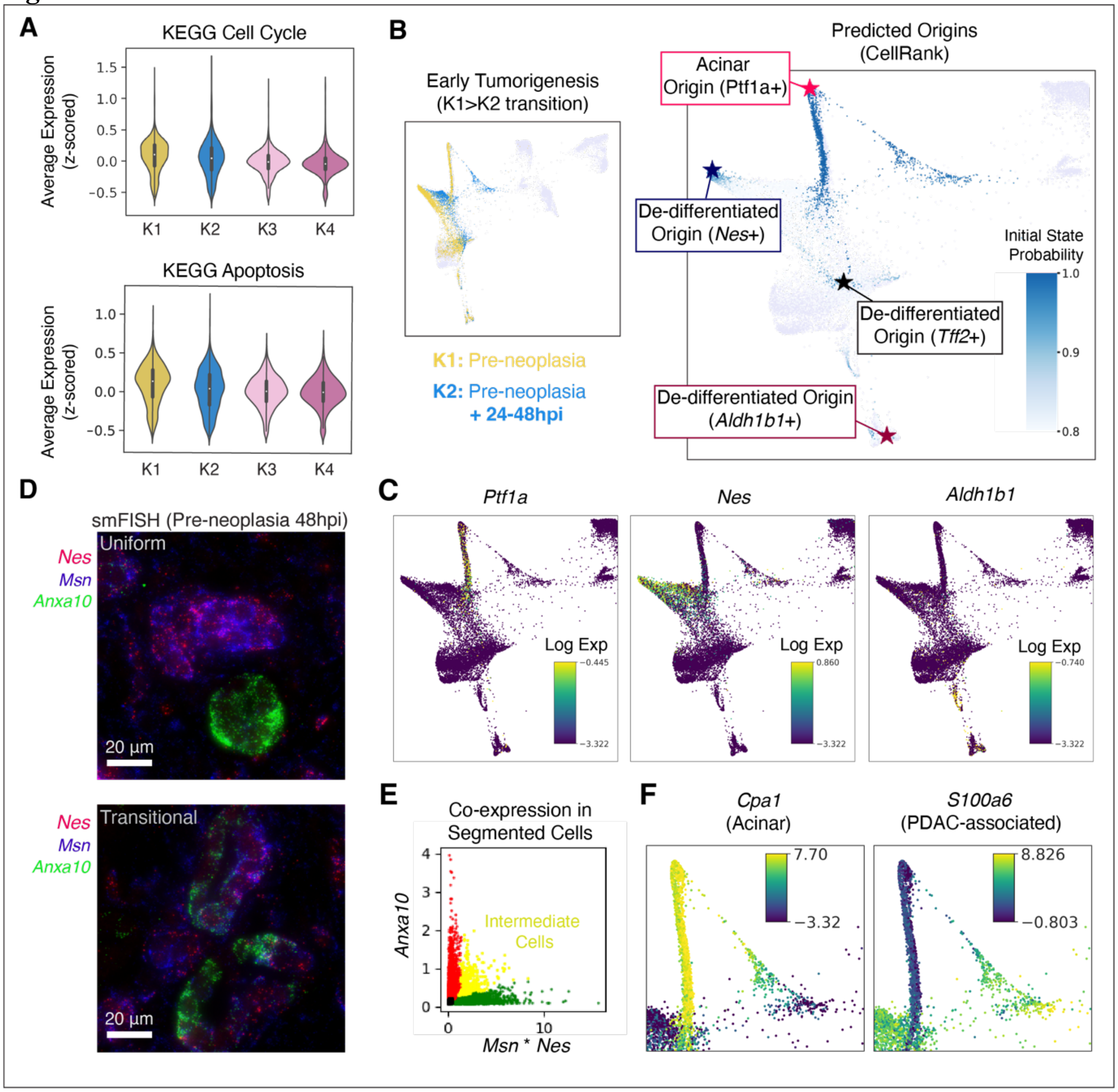

Identification of specific, injury-sensitive *Kras*-mutant subpopulations with multi-lineage potential.

(**A**) Distribution of cell cycle genes (top) and apoptosis genes (bottom) (*97*) expression in all epithelial cells derived from pre-malignant (*Kras* mutant) conditions K1–K4. Expression of these signatures across cells from conditions is computed as an average of z-scored, log-normalized expression per gene.

(**B**) CellRank (*39*) predicts multiple initiating populations of pancreatic tumorigenesis. Left, FDL of all *Kras*-mutant cells (K1–K6) as in **Fig. 2A**, colored to reveal pre-neoplastic cells (K1, K2). Right, region of the same FDL encompassing all K1 and K2 cells; cells are colored by CellRank-computed probability of being an initiating state. High-probability initiating states are indicated with stars and correspond (by marker gene expression in (C)) to initiating populations identified by lineage tracing studies (*26*, *42*–*45*). These include Nes+ (cluster 1), Aldh1b1+ (cluster 9), Tff2+ (cluster 0), and acinar (cluster 3) populations, using coarse PhenoGraph clustering in **Fig. S1B**.

(**C**) Region of FDL in (B) colored by expression of known cell-of-origin marker genes (*26*, *42*–*45*).
(**D**) smFISH data displaying spatial localization of markers for the progenitor-like state (*Nes*, *Msn*) and the gastric-like state (*Anxa10*). In two example images derived from the same mouse (K2 condition, *Kras*-mutant 48 hpi), there are individual lesions composed mainly of either progenitor-like states or gastric-like states ("uniform", top), and those composed of both progenitor-like and gastric states ("transitional", bottom). Scale bar, 20 µm.
(**E**) Expression of progenitor-like markers versus gastric markers in individual cells in smFISH data. Each point represents one segmented cell. Progenitor marker expression is quantified as the product of *Msn* and *Nes* expression in that cell. Cells are colored by their status as mainly progenitor-like (green), mainly gastric-like (red), intermediate (yellow, positive for both progenitor and gastric markers), or neither (black, negative for both progenitor and gastric markers).
(**F**) Region of the FDL in **Fig. 2A** containing the bridge population, which exhibits a gradual decrease in acinar-associated gene expression and concomitant increase in PDAC-associated gene expression.

Figure S4

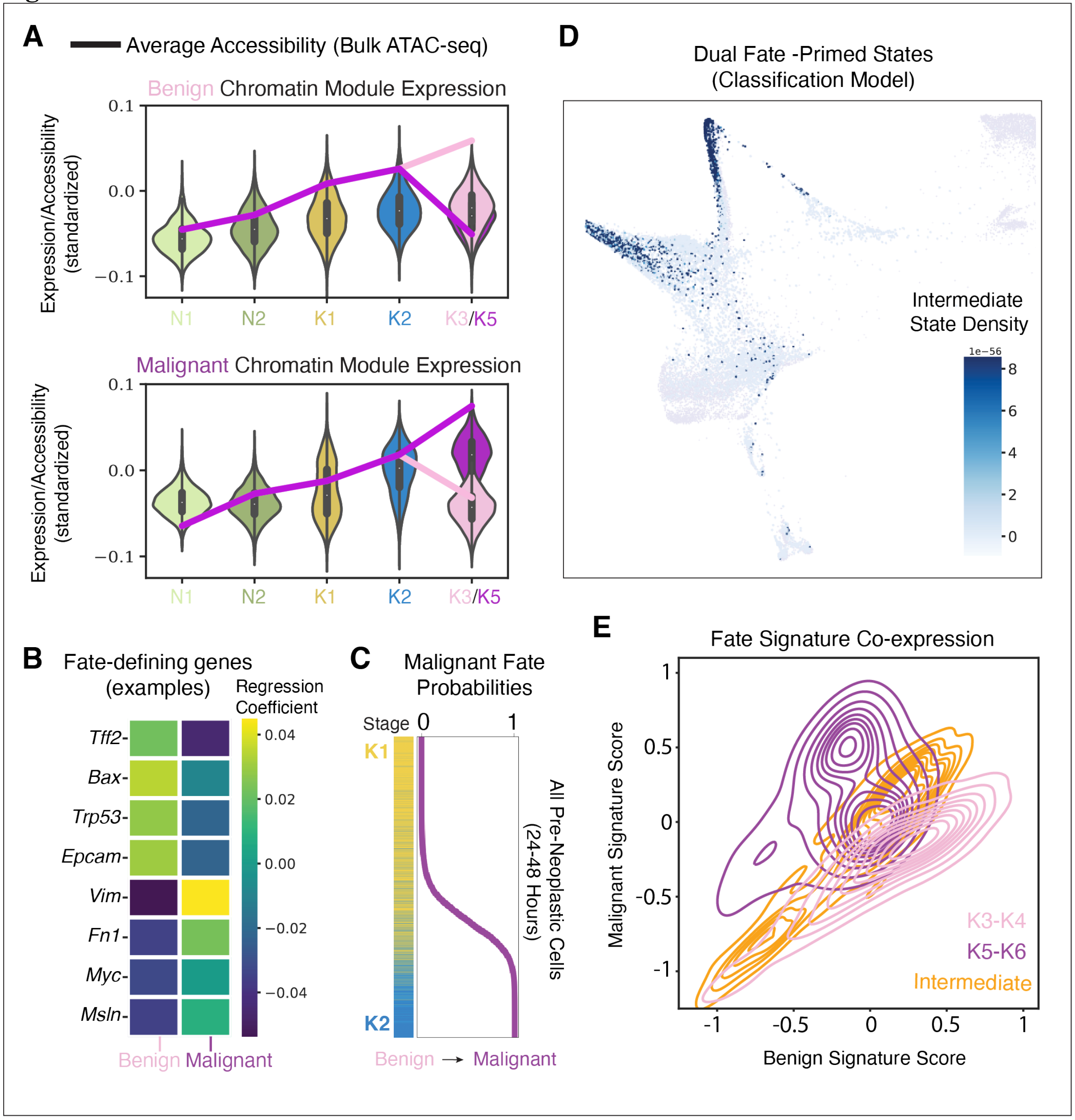

Epigenetic priming for distinct fates in *Kras*-mutant initiating populations.

(**A**) Expression of genes unique to benign or malignant chromatin modules, in all mKate2^{+} epithelial cells derived from the indicated *Kras* wild-type (N1-N2) and mutant (K1-K5) tissue conditions (violin plots). Expression values are z-scored across genes to emphasize the most dominant genes expressed in each cell. Average (standardized) accessibility from bulk ATAC-seq in each sample (lines), reveals a general correspondence between accessibility and expression trends. Substantial changes in malignant module expression between un-injured and injured cells is only observed in the context of mutant *Kras*.

(**B**) Logistic regression model coefficients for Benign (left) or Malignant (right) fates for select genes.

(**C**) Classification of all pre-neoplastic (K1, K2) cells to late-stage fates. Each row represents one cell from K1 (yellow) or K2 (blue) conditions, sorted from highest Benign fate probability (top)

to highest Malignant fate probability (bottom). Corresponding Malignant fate probability is plotted at right.
(**D**) FDL region as in **Fig. S3B**, highlighting cells with an intermediate probability ($0.4 < p < 0.6$) of classifying to a Benign or Malignant fate. Cells are colored by the density of intermediate cells in the phenotypic space (darker corresponds to more intermediate cells in the local region). Density was derived using a Gaussian kernel density estimate of intermediate cells in PC space.
(**E**) Intermediate cells exhibit evidence of dual priming for divergent fates. Contour plots display cell density based on expression of Benign and Malignant gene signatures for benign neoplasia (K3, K4) cells (pink), malignant (K5, K6) cells (purple), and intermediate (composite state) cells from K1 and K2 identified by the classification model (orange). Gene signatures are derived by intersecting genes with the top 200 largest coefficients in the classification models for K3 and K5 classes with genes specifically associated with bulk ATAC-seq benign and adenocarcinoma modules (see **Fig. 2C** and **Table S3**), respectively. A per-cell signature score was computed as the average z-scored expression of each gene in the signature. We observe that individual intermediate cells from K1 and K2 co-express benign and malignant programs.

Figure S5

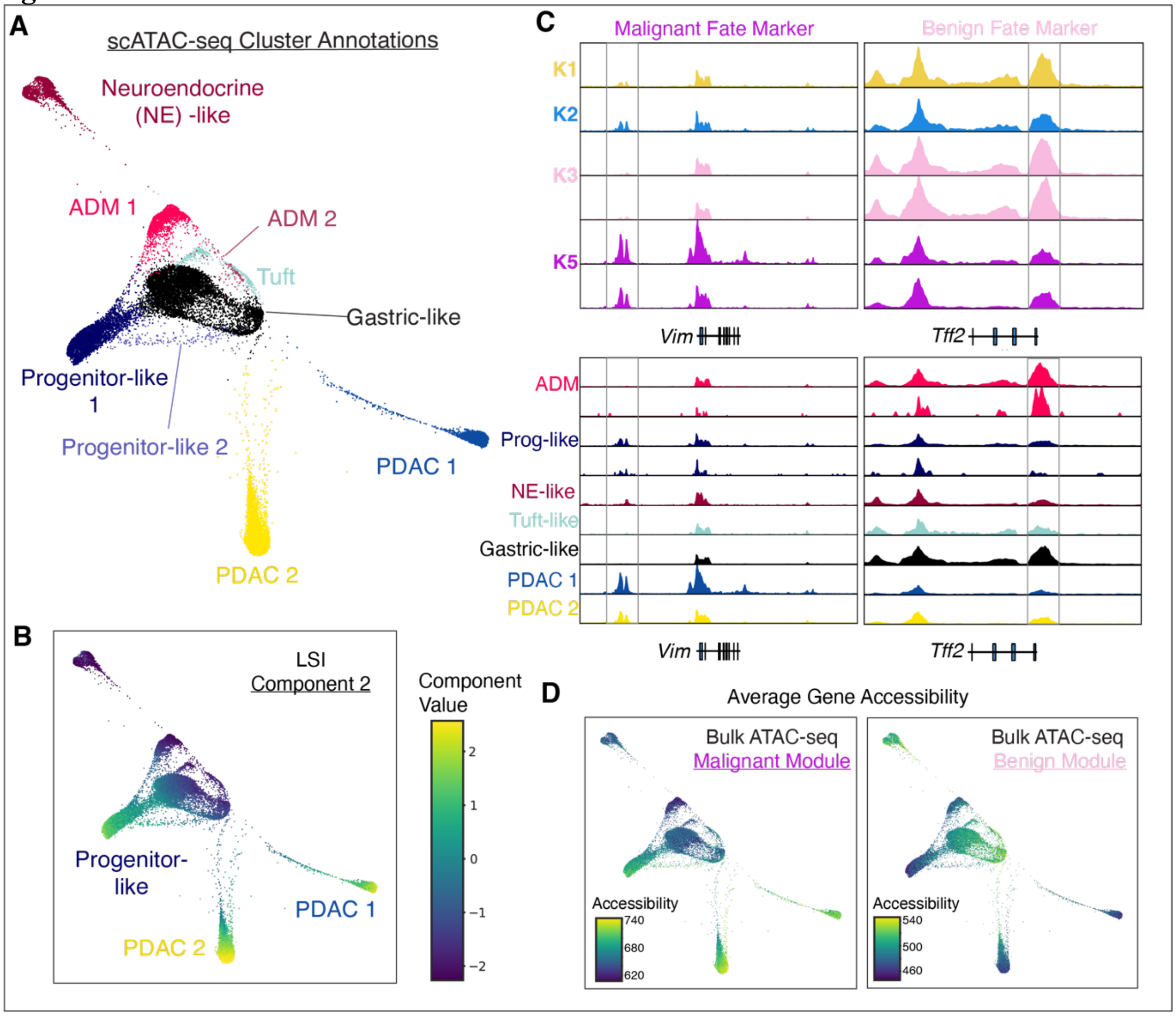

Epigenomic characterization of early and late *Kras*-mutant subpopulations.
(**A**) FDL of scATAC-seq profiles from pre-malignant (K1-K3) and malignant (K5) stages, colored by merged PhenoGraph cluster and annotated manually (*27*).
(**B**) FDL as in (A), colored by second component of LSI, revealing similarity between tumor and *Nes*$^+$ progenitor states at the chromatin level.
(**C**) Chromatin accessibility tracks for representative benign and adenocarcinoma-primed genes, aggregated across cells belonging to a given condition (top) or merged PhenoGraph cluster (bottom). x-axis, genomic coordinates around the indicated gene; y-axis, smoothed, depth-normalized counts of scATAC-seq fragments. *Tff2* is selected from the high-coefficient genes for benign neoplasia classifier (see **Fig. S4B**) and appears primed toward benign neoplasia in early tumorigenesis, whereas *Vim* is selected from the malignant classifier shows priming toward PDAC.
(**D**) FDL of scATAC-seq profiles from pre-malignant and malignant stages, colored by sum of accessibility near genes (ArchR gene score) associated with Benign and Malignant chromatin modules (**Table S3**). Cells with high accessibility for these fate-associated genes fall within distinct regions of the map. Similar to patterns observed in **Fig. 2C**, Malignant programs are activated in *Nes*$^+$ progenitor-like cells, where benign programs are enriched in gastric-like cells.

Figure S6

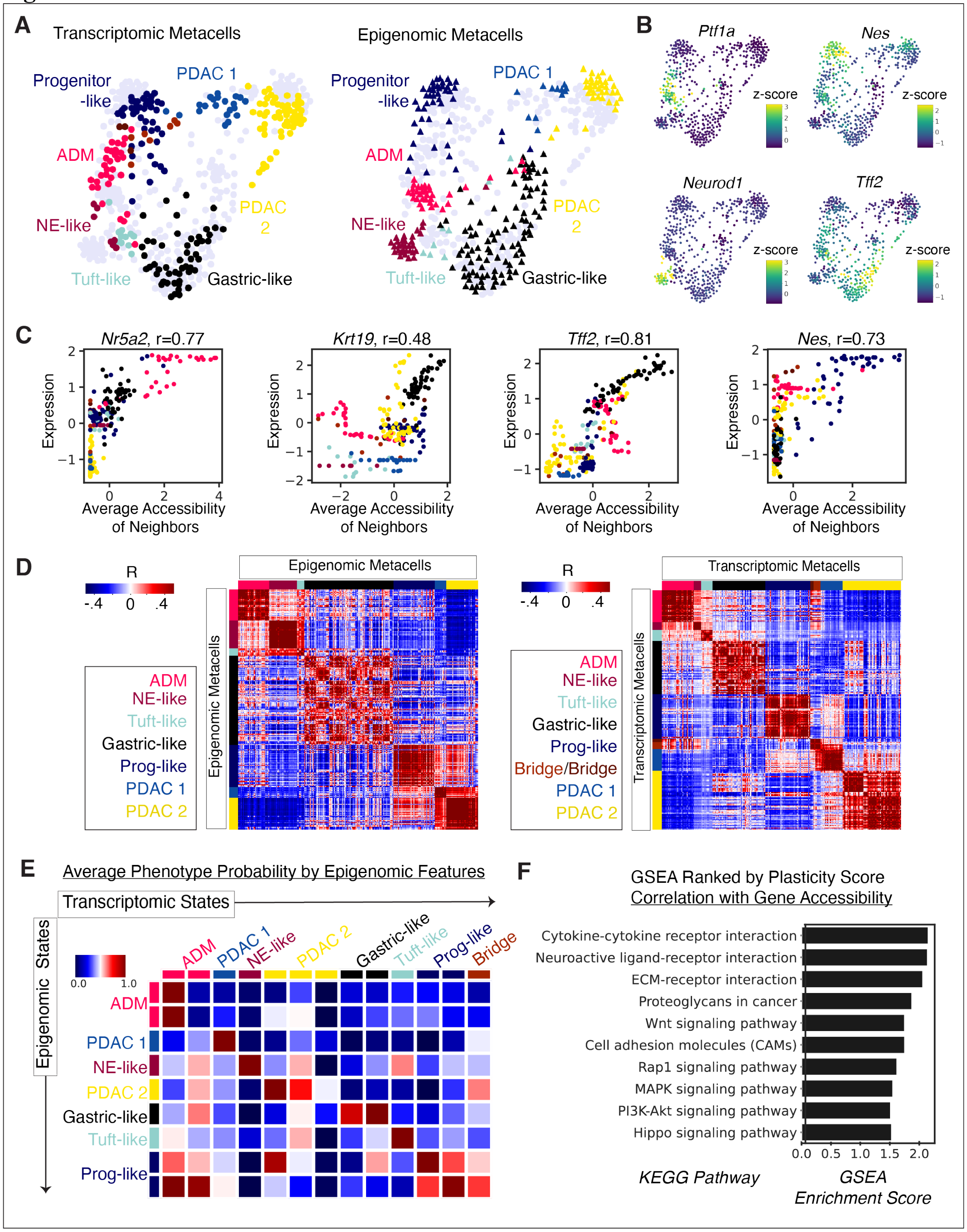

Integration of tumorigenesis-associated epigenomic and transcriptional cell states.
(**A**) Integrated FDL visualization of metacells derived from scATAC-seq (triangles, right) and scRNA-seq (circles, left), with nodes colored by PhenoGraph cluster from each respective technology (**Figs. S1C** and **S5A**). The FDL is built on a composite graph which combines

within-modality nearest neighbors with cross-modality mutually nearest neighbors (MNN) to emphasize similarities between cells both within and across modalities (*27*).
(**B**) FDL as in (A), colored by expression score (the average of log-normalized counts from all cells in a metacell) or accessibility score (average of log-normalized ArchR gene accessibility scores) for each metacell, for a selection of known cell-state markers. Values are z-scored within each modality to obtain comparable scales. Acinar (*Ptf1a*), progenitor-like (*Nes*), neuroendocrine-like (*Neurod1*), and gastric-like (*Tff2*) markers show high concordance between modalities on the visualization.
(**C**) Per-gene correlations of accessibility and expression across paired metacells. Each point corresponds to one transcriptomic metacell, colored by annotation (color as indicated in (A)) of PhenoGraph clusters (refined transcriptomic clusters; see **Fig. S1C** and **S5A**). Y-axis displays z-scored expression of the indicated gene in each metacell; x-axis displays the average z-scored accessibility score across the epigenomic metacell MNNs of that transcriptomic metacell. High correlation values indicate strong concordance between expression of a gene and its accessibility for matched epigenomic profiles.
(**D**) Pairwise Pearson correlation coefficients of metacells derived from scATAC-seq (left) and scRNA-seq (right). Metacells are ordered by manually annotated PhenoGraph clusters (refined transcriptomic clusters; see **Figs. S1C** and **S5A**). Major blocks of high positive correlation along the diagonal represent accessibility- and expression-derived cell-states that are highly similar within each modality. Off-diagonal correlations represent similarity between distinct cell-states. Quantifying the difference between intra-cluster (r_{intra}) and inter-cluster (r_{inter}) correlations, we find that intra-cluster similarity is much greater ($r_{intra} - r_{inter} = 0.51$ for RNA and 0.42 for ATAC) than cross-modality similarity ($r_{intra} - r_{inter} = 0.26$).
(**E**) Average phenotype probability by epigenomic features. Heatmap displays average log probability of classification to each transcriptomic cluster (columns) from metacells of each epigenomic cluster (rows). Row and column order correspond to that in the confusion matrix in **Fig. 3E**. Color values indicate the full log probability distribution (as opposed to the count of discrete predictions for cells of each epigenetic cluster depicted in **Fig. 3E**).
(**F**) GSEA enrichment scores for select significantly (FDR < 0.1) enriched gene sets in genes ranked by Spearman correlation to inferred plasticity. High enrichment indicates a significant positive association of that program at the chromatin level with cell-states maintaining high plasticity as defined by the method in **Fig. 3D**.

Figure S7

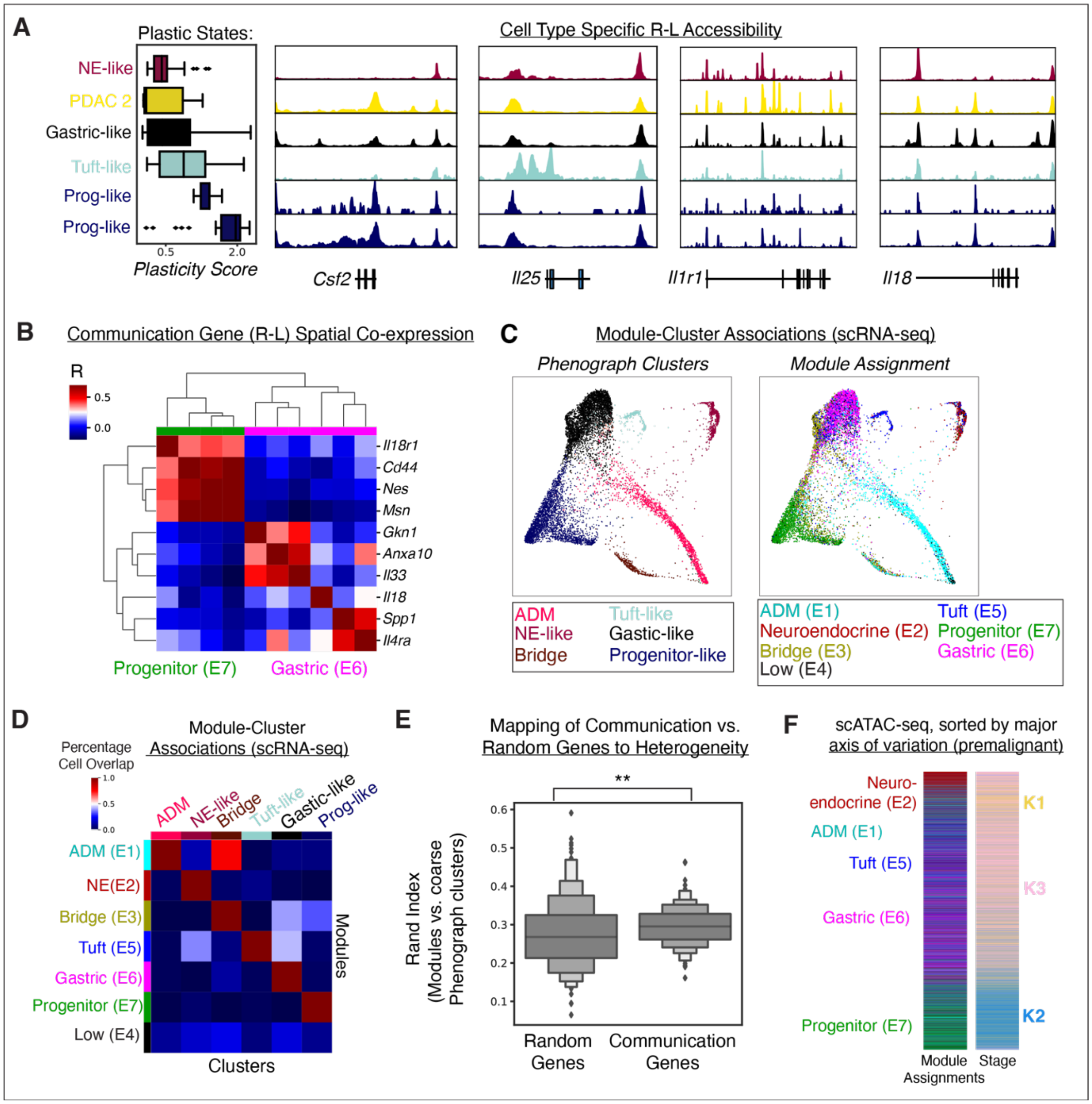

Distinct cell-cell communication modules are associated with defined neoplastic lineages.
(**A**) Chromatin accessibility signal tracks from scATAC-seq data for select communication genes aggregated across cells from each PhenoGraph cluster of plastic cell-states. Clusters are ordered by increasing plasticity (reproduced at left here from **Fig. 3F**), highlighting greater accessibility near these genes in high-plasticity populations.
(**B**) Pearson correlation between each pair of communication genes (or corresponding markers) across all segmented cells in smFISH data. The Calligraphy communication module for each R-L gene (or associated marker) is denoted in color on the columns, where magenta corresponds with the gastric (E6) module and green corresponds with the progenitor (E7) module. Hierarchical clustering (visualized as dendrograms) on rows and columns groups genes based on their co-expression patterns across space in the tissue.

(**C**) FDL of pre-malignant epithelial cells (K1–K3, see **Fig. 4B**), colored by coarse PhenoGraph cluster (see **Fig. S1B**) (left) or communication module assignment (right). Module expression is computed as the log of average normalized expression of each gene in that module; cells are assigned to the highest-expressed module.
(**D**) Correspondence between cell membership in coarse PhenoGraph clusters computed on all genes (columns) and communication modules computed only on communication genes (rows). Color values represent the proportion of cells in each cluster which map to each module, revealing tight concordance for all modules except Low (E4).
(**E**) Boxplots displaying distribution of rand indices (against coarse PhenoGraph clusters) from modules defined from randomly selected genes versus down-sampled communication genes. Asterisks indicate significance in a one-tailed, un-paired t-test (p value < 0.01). Modules derived from communication genes are significantly more similar to global heterogeneity (clustering) than modules derived from random genes.
(**F**) Communication module assignments of cells from chromatin accessibility data, based on average normalized accessibility (ArchR gene score) of communication genes within each module (*27*). Cells are ordered along the second component from LSI computed on scATAC-seq data.

Figure S8

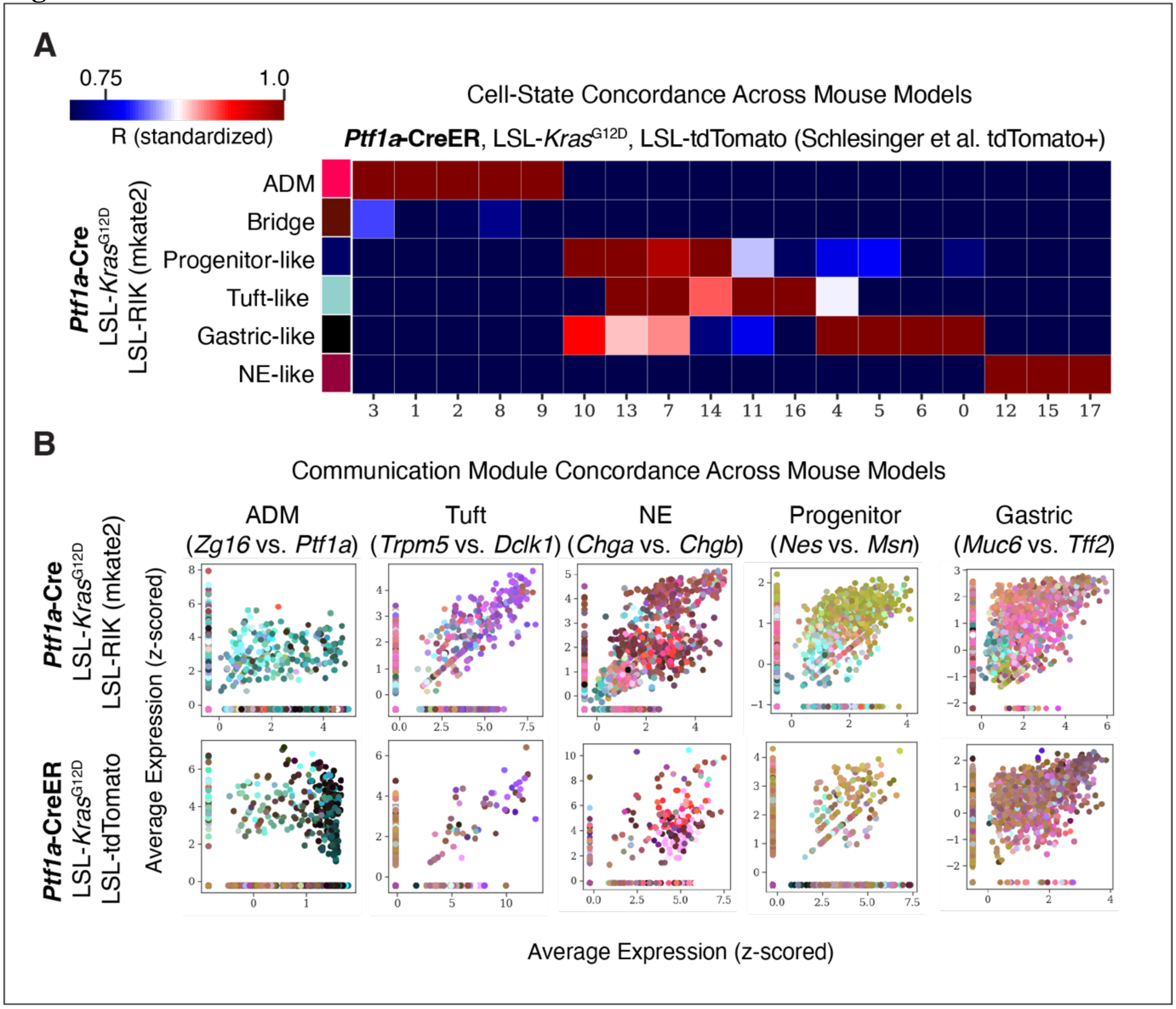

Concordance of global heterogeneity and communication modules in alternate model systems.

(**A**) Pairwise correlation of average expression per coarse PhenoGraph cluster derived from pre-malignant *Kras* mutant cells (rows) and per cluster identified in a comparable scRNA-seq dataset with mutant *Kras* activated in the adult via Cre-ER (*52*). The majority of cell-states have clear correspondence based on high correlation with several clusters in the complementary dataset.

(**B**) Scatter plots of normalized expression per cell of indicated marker genes in our data (top) and data from (*52*) (bottom). Cells are colored by relative module expression, computed as the log of average normalized expression of each gene in the module. Cell "pseudo-color" values correspond to a mixture of module-specific colors based on the relative expression of module genes in each cell (*27*). There is a high degree of correspondence between module expression and cell-state definitions.

Figure S9

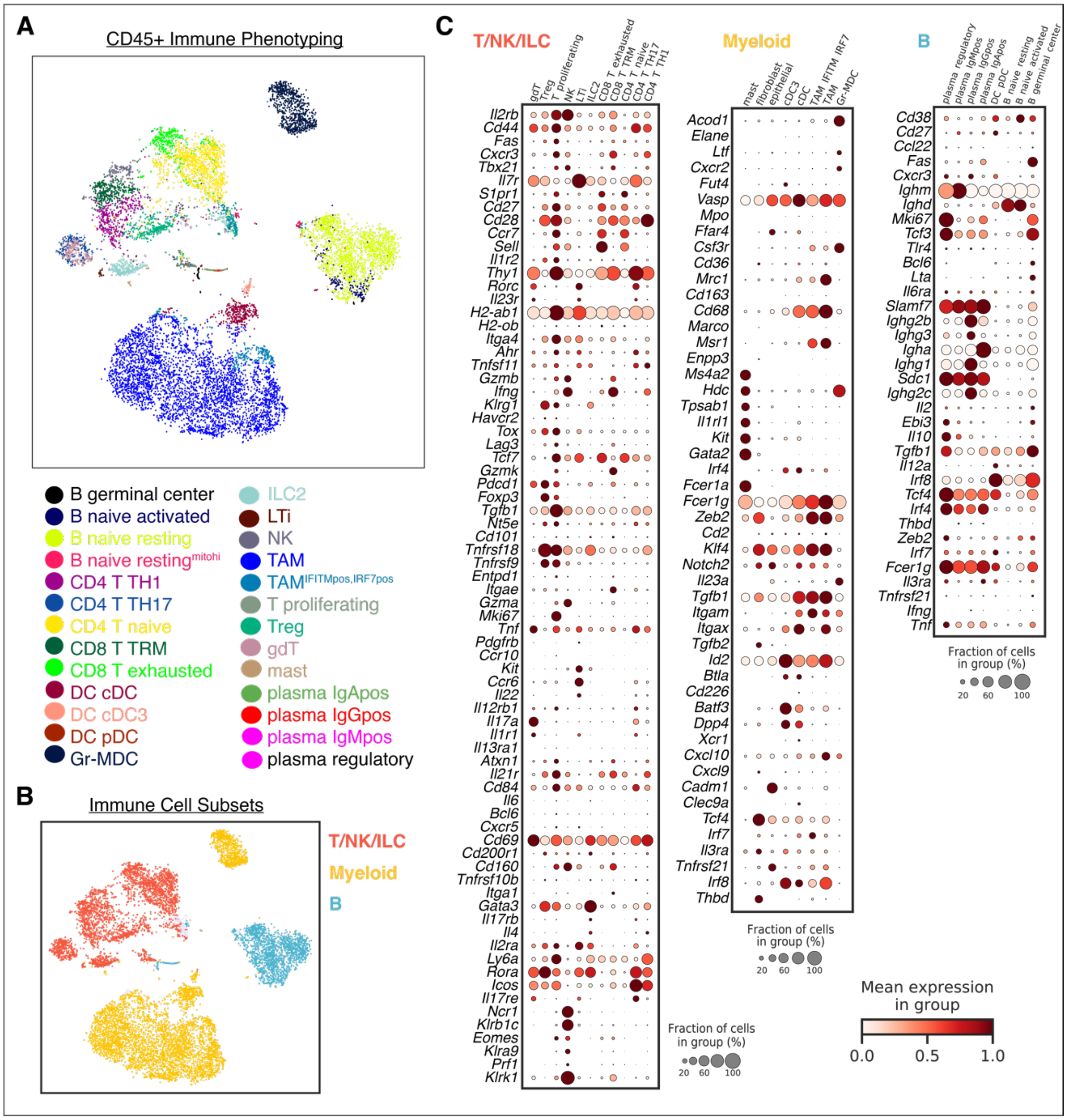

Characterization of immune heterogeneity in pancreatic tissue.

(**A**) tSNE visualization (scRNA-seq) of CD45^{+}-sorted immune populations from pre-malignant pancreata (K1–K3) colored by cell type annotation.

(**B**) tSNE as in (A) displaying coarse immune cell subsets combining all T, NK, and ILC - derived cells, myeloid -derived cells, or B cell -derived cells into a group for downstream co-expression analysis and module determination (see **Figs. S10B,C**).

(**C**) Immune marker expression (rows) across PhenoGraph clusters from CD45^{+}-sorted immune cells in scRNA-seq (columns). Each dot plot shows cell-type -specific expression patterns in clusters derived from T/NK/ILC, Myeloid and B cell subsets from left to right. The size of each dot scales with the number of cells in each cluster (columns) expressing the gene in each row. The color of each dot scales with the mean expression of that gene across all cells in that cluster.

Figure S10

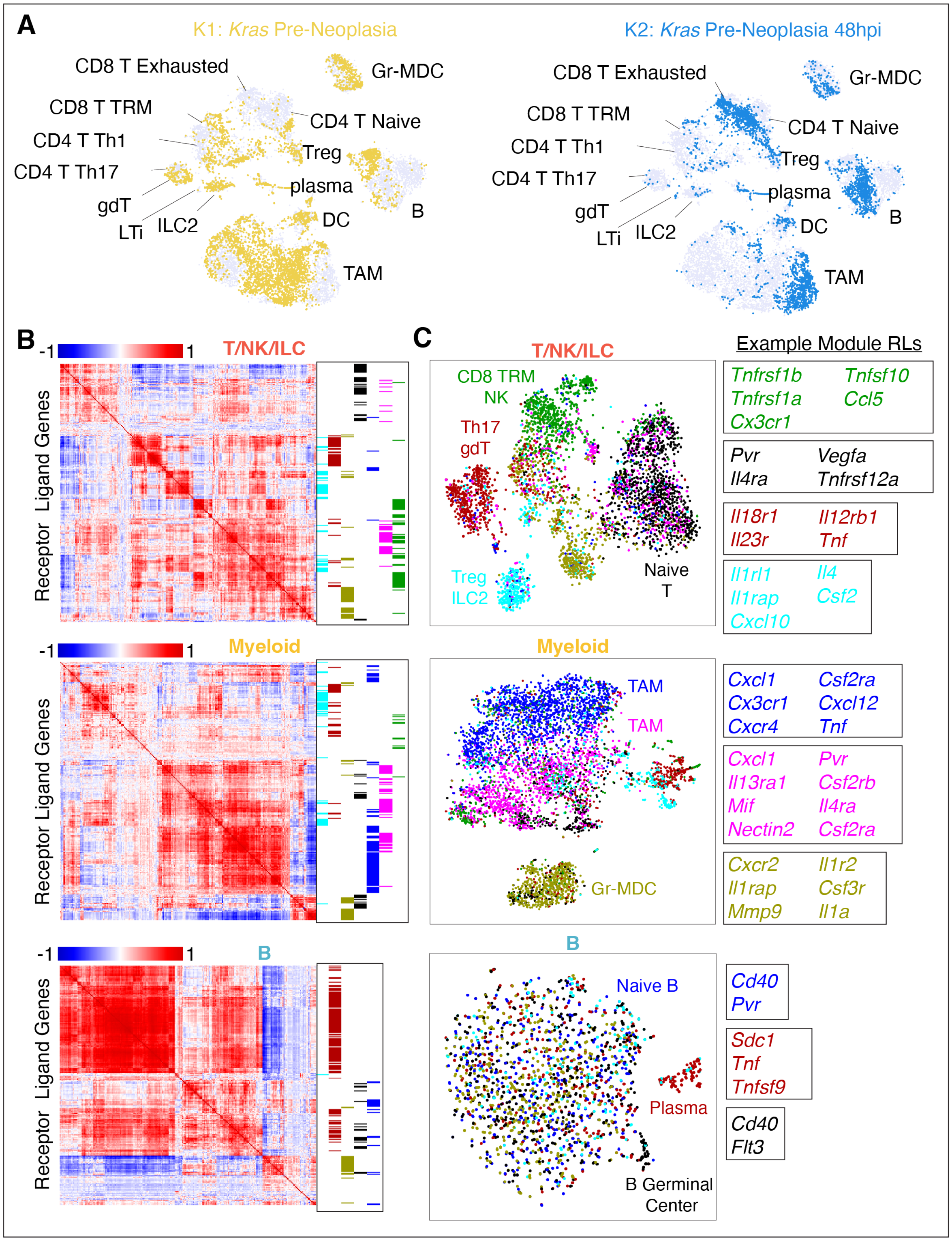

Communication module prediction in infiltrating immune cells.
(**A**) tSNE visualization of CD45^{+}-sorted immune populations from pre-malignant samples (K1–K3, see **Figs. S9A,B**). Coloring K1 (yellow) and K2 (blue) cells separately reveals the dramatic shifts in immune phenotypes that occur in *Kras*-mutant pancreata upon injury.

(**B**) Communication gene co-expression modules in CD45^{+} immune cells derived from pre-malignant tissues separated into three major immune subsets as in **Fig. S9B**. Each row or column corresponds to one receptor or ligand, and color values represent the Pearson correlation coefficient between the expression of a pair of genes across cells of that subset. Blocks of highly correlated communication genes along the diagonal correspond to partially overlapping modules of genes that tend to be expressed in the same cell populations. Each column of inferred communication gene co-expression modules from the OSLOM community detection algorithm (right) depicts genes belonging to a single module.
(**C**) tSNE visualization of major immune subsets in **Fig. S9B** with cells colored by module assignment from **Fig. S10B**. For each subset, modules are computed using OSLOM community detection (*27*), cells are assigned to the module with highest average z-scored, log-normalized expression. Right, representative communication genes from select modules. Individual genes can be shared between modules (for example, CD40 in B cells) when genes are expressed in more than one cell type.

Figure S11

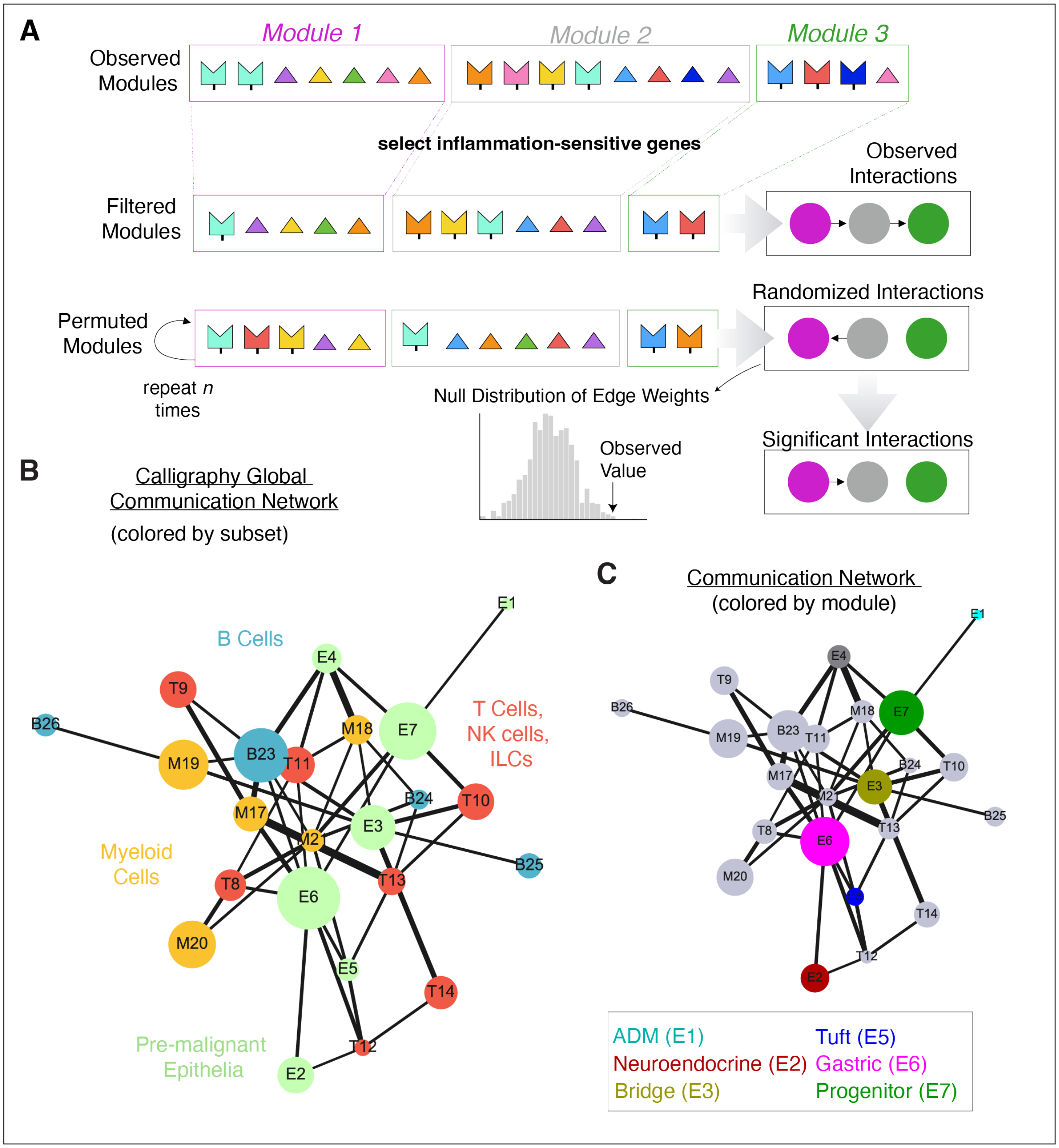

Calligraphy predicts tissue crosstalk networks between *Kras*-mutant cells and infiltrating immune cells recruited to their tissue environment.

(**A**) Schematic of the module-based crosstalk algorithm, Calligraphy. Observed modules are first identified from a gene-gene co-expression graph of cell subpopulations using the OSLOM community-based detection approach (*99*). Each module contains a set of receptors (boxes) and ligands (triangles) which are co-expressed across subpopulations of scRNA-seq data, colored by cognate pairs known to physically interact. Inferred gene modules are then filtered for communication genes that are upregulated in early tumorigenesis (K2) compared to normal regeneration (N2). Cognate R-L pairs spanning filtered modules are enumerated to suggest

potential cross-module interactions. Then, modules are randomly permuted *n* times and cross-module interactions are recounted in each trial to derive a null distribution for each pairwise module-module interaction. A p value is obtained for each pair of modules using this null distribution.
(**B**) Global crosstalk network inferred by Calligraphy, colored by cell subset. Each node represents one module, with size proportional to the number of communication genes in that module. Edges connect significant module-module interactions, with widths proportional to the significance of interaction (–10 log(p value + pseudocount)).
(**C**) Global crosstalk network inferred by Calligraphy, colored by epithelial module as in **Fig. 4A**, or gray for immune modules.

Figure S12

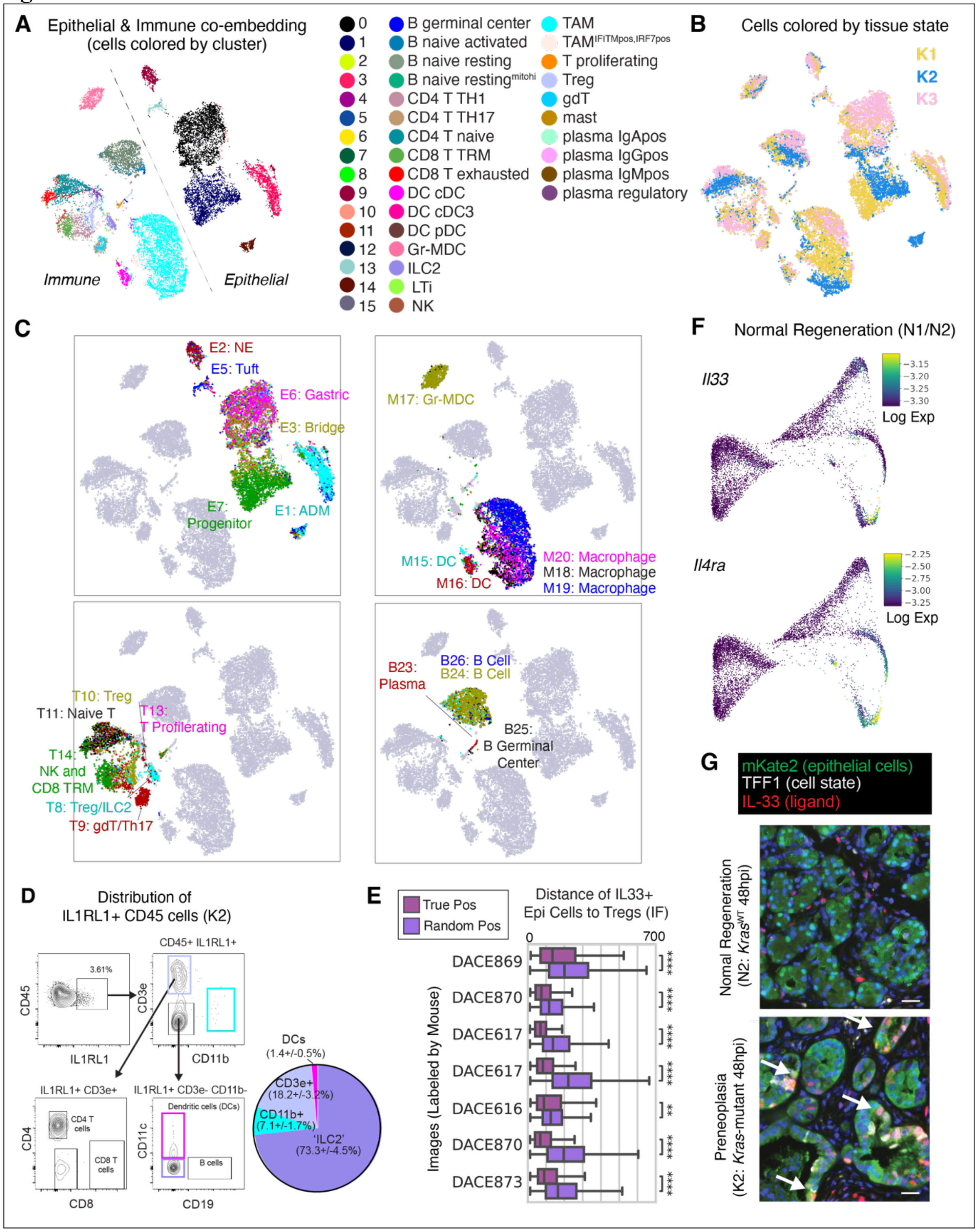

Identification of *Kras*-mutant specific epithelial-immune crosstalk networks.
(**A**) tSNE of integrated immune and epithelial scRNA-seq data from pre-malignant stages (K1-K3, see **Fig. 5B**) colored by PhenoGraph cluster (coarse epithelial clusters, see **Fig. S1B**).
(**B**) tSNE as in (A), colored by progression stage.

(**C**) tSNE as in (A), separated by epithelial (top left), myeloid (top right), T/NK/ILC (bottom left), and B cell (bottom right) compartments. Cells of each compartment are colored in each plot by their module assignments. Module expression is computed as the log of average normalized expression of each gene in that module; cells are assigned to the highest-expressed module of the corresponding subset.
(**D**) Representative FACS plots indicating gating strategy to characterize IL1RL1 positivity in myeloid and lymphoid compartments of *Kras*-mutant pancreata at 48 hpi (left). The fraction of lymphoid and myeloid cells among IL1RL1^{+} immune cells in *Kras*-mutant pancreata at 48 hpi are quantified in the pie chart, consistent with the expected positivity in CD4^{+} T-reg cells (CD3^{+}, CD11b^{-}), DCs (CD3^{-}, CD11b^{-}CD11c^{+}), Non DC-myeloid (CD11b^{+}CD3^{-}CD11c^{-}), and ILC2 (CD3^{-}, CD11b^{-}, CD11c^{-}). Pooled data are presented as mean ± s.e.m (n = 6 independent mice).
(**E**) Boxplots comparing distance (in pixels, 0.325 µm per pixel) *in situ* of IL33^{+} epithelial cells to Tregs against a null model of spatial distribution in co-immunofluorescence (co-IF) data from the indicated biological replicates (independent mice) from K2-tissue state (*Kras*-mutant + injury, 48 hpi condition). Each pair of boxes across the y-axis is for one co-IF image collected. For each image, distances in the upper distribution are calculated between each IL-33^{+} epithelial (E-cadherin^{+}) cell and its closest Treg (CD3^{+}FOXP3^{+}). Distances in the lower distribution are calculated by randomly permuting positions of epithelial cells and re-computing distances for multiple trials. Asterisks indicate statistically significant differences in a one-tailed, un-paired t-test. In these cases, IL-33^{+} cells are closer to Tregs than other epithelial cells, on average. Pooled data are presented as mean ± s.e.m (minimum 128 distances analyzed per comparison).
(**F**) FDL of normal regeneration (N1, N2, see **Fig. 4C**) colored by MAGIC imputed log-normalized expression of *Il33* (middle) or *Il4ra* (bottom). Colors are scaled between the 1st and 99th percentile.
(**G**) Representative immunofluorescence of IL-33 (red), TFF1 (white) or mKate2 (epithelial cells, green), visualizing the selective injury-driven induction of IL-33 in a subset of *Kras*-mutant pancreatic epithelial cells expressing the gastric-cluster marker TFF1 (bottom) but not in *Kras*-wild-type injured counterpart (top). Scale bar, 20 µm. The bottom panel section is also shown in **Fig. 6B**.

Figure S13

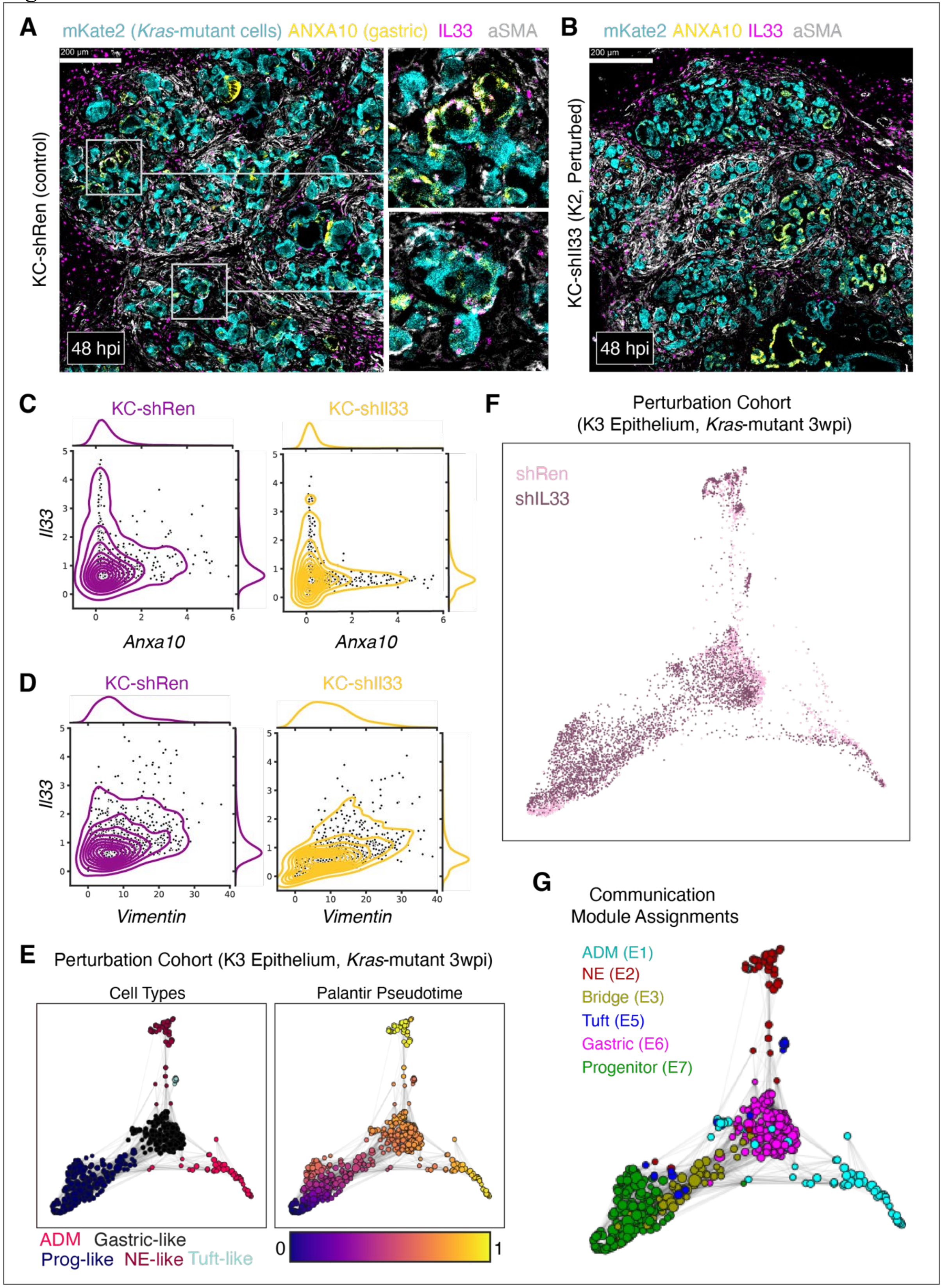

Impacts of *Il33* perturbation assessed with IMC and scRNA-seq.

(**A**) Imaging Mass Cytometry (IMC) staining of pancreatic tissues from KC-shRen mouse (48 hpi, K2 stage) showing activation of IL-33 (magenta) within mKate2 (marking epithelial cells, cyan) cells positive for gastric state marker (ANXA10, yellow), as well as in surrounding stroma. Scale bar, 200 µm.
(**B**) IMC staining of pancreatic tissues from KC-sh*Il33* mouse (48 hpi, K2 stage) showing depleted expression of IL-33 (magenta) in mKate2$^+$ *Kras*-mutant epithelial cells (cyan), and intact IL-33 expression fibroblasts marked by aSMA (white). Scale bar, 200 µm.
(**C**) Scatterplots with density contours indicating co-expression of ANXA10 (x-axis) and IL-33 (y-axis) in 30 pixel square patches (roughly representing single lesions) of a control K2 (left) and an sh*Il33* K2 (right) IMC image. sh*Il33* represses the spatial co-expression relationship between ANXA10 and IL-33.
(**D**) Scatterplots with density contours indicating co-expression of VIM (x-axis) and IL-33 (y-axis) in 30 pixel square patches of a control K2 (left) and an sh*Il33* K2 (right) IMC image. sh*Il33* does not substantially impact the spatial co-expression relationship between VIM (marking primarily stromal elements) and IL-33.
(**E**) Milo neighborhoods overlaid on FDL built on scRNA-seq of K3 control and sh*Il33* cells. Each point represents one neighborhood scaled to size (number of cells) and is colored by either cell-state annotation (left) or Palantir pseudotime (right).
(**F**) FDL built on scRNA-seq of K3 control and sh*Il33* cells, with cell color indicating its condition.
(**G**) Milo neighborhoods overlaid on FDL in (F) colored by communication module assignment. Module expression per neighborhood is computed as the log of average normalized expression of each gene in that module; neighborhoods are assigned to the highest-expressed module.

Table S1.

Sample and GEMM information, provided as a separate supplementary file.

Table S2.

**Cell Type**	**Markers**
Acinar (differentiated)	*Zg16, Cpa1*
Ductal	*Krt19, Sox9, Clu*
Nes^{+} Progenitor	*Nes, Cd44, Vim, Cdkn2a*
Tuft	*Pou2f3, Dclk1, Trpm5*
Neuroendocrine (NE)	*Chga, Chgb, Neurod1, Syp, Ppy, Gcg, Ins, Sst*
Gastric	*Muc1, Muc6, Gkn3, Muc5ac, Tff2, Tff1, Agr2*

scRNA-seq Annotation Strategy.

Table S3.

Benign and Malignant Signature Genes, provided as a separate supplementary file.

Table S4.

Plasticity score Gene Set Enrichment Analysis, provided as a separate supplementary file.

Table S5.

Plasticity Score Gene Correlations, provided as a separate supplementary file.

Table S6.

Calligraphy Communication Modules, provided as a separate supplementary file.

Table S7.

Calligraphy Module-Module Interactions, provided as a separate supplementary file.

Table S8.

Condition	Cohort	Oncogenic mutations	Pancreatitis treatment	Timepoint
N1	Progression (Normal)	No (*Kras^{wt}*, *Trp53^{wt})*	No (PBS control)	2 days post-PBS
N2	Progression (Regeneration)	No (*Kras^{wt}*, *Trp53^{wt})*	Yes	2 days post-caerulein
K1	Progression (Pre-malignant)	*Kras^{G12D}*	No (PBS control)	2 days post-PBS
K2	Progression (Pre-malignant)	*Kras^{G12D}*	Yes	2 days post-caerulein
K3	Progression (Pre-malignant)	*Kras^{G12D}*	Yes	21 days post-caerulein
K3.5	Progression (Pre-malignant)	*Kras^{G12D}*	No (PBS control)	21 days post-PBS
K4	Progression (Pre-malignant)	*Kras^{G12D}*	No (natural progression)	27 week old mice
K5	Progression (Malignant)	*Kras^{G12D}; p53-null or mutant (p53^{R172H}*)	No (natural progression)	PDAC formation (primary tumor)
K6	Progression (Malignant)	*Kras^{G12D}; p53-null or mutant (p53^{R172H}*)	No (natural progression)	PDAC formation (metastasis: liver or lung)
K2 + sh*Il33*	Perturbation	*Kras^{G12D}* (+ sh*Il33*)	Yes	2 days post-caerulein

K3 + sh*Il33*	Perturbation	*Kras*^{G12D} (+ sh*Il33*)	Yes	21 days post-caerulein
K2 + shControl	Perturbation	*Kras*^{G12D}	Yes	2 days post-caerulein
K3 + shControl	Perturbation	*Kras*^{G12D}	Yes	21 days post-caerulein

Experimental conditions.

Table S9.

**Antibody**	**Company**	**Catalog#**	**RRID**	**Metal**
mKate2	Evrogen	AB233	AB_2571743	142Nd
Vimentin	Fluidigm	3143027D	not available	143Nd
IL-33	R&D systems	AF3626	AB_884269	152Sm
ANXA10	Abcam	ab223131	not available	154Sm
FoxP3	Cell Signaling Technology	12653	AB_2797979	158Gd
aSMA	Abcam	ab5694	AB_2223021	175Lu
GFP	Abcam	ab220802	not available	176Yb
DNA1	Fluidigm	201192B	not available	191Ir
DNA2	Fluidigm	201192B	not available	193Ir

IMC Metal-conjugated primary antibodies.

Table S10.

smFISH information, provided as a separate supplementary file.

Table S11.

Short name	Alleles
C	*Ptf1a-cre;RIK* (C;RIK)
KC	*Ptf1a-cre;RIK;LSL-Kras*^{G12D} (KC;RIK)
KPC	*Ptf1a-cre;RIK;LSL-Kras*^{G12D}*;p53*$^{fl/+}$<br>*Ptf1a-cre;RIK;LSL-Kras*^{G12D}*;p53*$^{R172H/+}$ (KPC;RIK)
KC-sh*Ren*	*Ptf1a-cre;RIK;LSL-Kras*^{G12D}*;TRE-GFP-shRen.713*<br>*(*KC;RIK-shRen.713)
KC-sh*Il33*	*Ptf1a-cre;RIK;LSL-Kras*^{G12D}*;TRE-GFP-shIl33.668*<br>*Ptf1a-cre;RIK;LSL-Kras*^{G12D}*;TRE-GFP-shIl33.327*<br>*(*KC;RIK-shIl33.668 or KC;RIK-shIl33.327)

GEMM Alleles.

Table S12.

Cohort	Sample ID	Condition	Genotype	Filtering Group	No. of Cells	Median Library Size
Progression	DACD511_Kate_plus	N1	C;RIK	1	2491	4534
Progression	DACD550_kate_plus	N1	C;RIK	1	2890	5855
Progression	DAC_B530-Kate+	N2	C;RIK	2	2804	5610.5
Progression	DACD403_Kate_plus	N2	C;RIK	2	1424	5309.5
Progression	DACD394_Kate_plus	K1	KC;RIK-shRen.713 (Off Dox)	2	2834	7562.5
Progression	DACD406_Kate_plus	K1	KC;RIK	2	2189	10276
Progression	DACD351-Kate+	K1	KC;RIK-shRNA (Off Dox)	2	2781	17184
Progression	DAC_C263_EPI	K1.5	KC;RIK-shRNA (Off Dox)	2	1851	14922
Progression	D396_EPI	K1.5	KC;RIK-shRNA (Off Dox)	2	2776	1732.5
Progression	DACD404_Kate_plus	K2	KC;RIK	2	1595	10470
Progression	DACD407_Kate_plus	K2-Day 1	KC;RIK	2	2075	12076
Progression	DAC_DI143_Epi	K3	KC;RIK	2	2423	15665
Progression	DACD482_Kate_plus	K3	KC;RIK	2	2668	10252.5
Progression	DAC_C301-EPI_1	K4	KC;RIK	2	1812	2433.5
Progression	DAC_C301-EPI_2	K4	KC;RIK	2	1142	615.5

Progression	Ag-PDAC-PT-Kate	K5	KPC;RIK (KPR172HC;RIK)	2	3387	15828
Progression	Ag-Lung-Mets-Kate	K6	KPC;RK (KPR172HC;RIK)	3	389	40691
Progression	DAC_D020_p5_Epi	K5	KPflC;RIK	2	3646	22107
Progression	DACC963PT_Kate_plus	K5	KPflC;RIK	2	679	5941
Progression	DACC963LIVERmet	K6	KPflC;RIK	2	2244	30530
Progression	DACC963_mKate_plus	K6	KPflC;RIK	2	1863	30134
Perturbation	DACD350-Kate+	K2 Control	KC;RIK-shIl33.668 (Off Dox)	2	3088	15149.5
Perturbation	DACE621-mKate2	K2 Control	KC;RIK-shRen.713 (On Dox)	4	2670	18234
Perturbation	DACD346-Kate+	K2 + IL33 KD	KC;RIK-shIl33.668 (On Dox)	2	3067	16170
Perturbation	DACE604-mKate2	K2 + IL33 KD	KC;RIK-shIl33.668 (On Dox)	4	3178	16897.5
Perturbation	DACE605-mKate2	K2 + IL33 KD	KC;RIK-shIl33.668 (On Dox)	4	2792	18296
Perturbation	DACD349_mKate2+;GFP+	K3 Control	KC;RIK-shIl33.668 (Off Dox)	2	903	18997
Perturbation	DACE610-EPI	K3 Control	KC;RIK-shIl33.327 (Off Do)	4	1053	20917
Perturbation	DACD347_mKate2+;GFP+	K3 + IL33 KD	KC;RIK-shIl33.668 (On Dox)	2	1049	18285
Perturbation	DACE607-EPI	K3 + IL33 KD	KC;RIK-shIl33.668 (On Dox)	4	2082	15419.5

Perturbation	DACE614-EPI	K3 + IL33 KD	KC;RIK-shIl33.327-(OnDox)	4	1255	16008
Immune	DACD407_CD45+_Day1	K2-Day 1	KC;RIK	5	1937	2007
Immune	DACD404_CD45	K2	KC;RIK	5	918	4243.5
Immune	DACD143_CD45+	K3	KC;RIK	5	1825	1752
Immune	DACD482_CD45+	K3	KC;RIK	5	2186	2779.5
Immune	DACD406-CD45+	K1	KC;RIK	5	2250	5860
Immune	DACD408-CD45+	K1	KC;RIK	5	2167	2465
Immune	DACD351-CD45+	K1	KC;RIK-shIl33.668 (Off Dox)	5	2033	8389
Immune	DACD350-CD45+	K2 Control	KC;RIK-shIl33.668 (Off Dox)	5	2414	1752
Immune	DACE621-CD45	K2 Control	KC;RIK-shRen.713 (On Dox)	6	3272	1724
Immune	DACD346-CD45+	K2 + IL33 KD	KC;RIK-shIl33.668 (On Dox)	5	2047	8017
Immune	DACE604-CD45	K2 + IL33 KD	KC;RIK-shIl33.668 (On Dox)	6	2068	1802.5
Immune	DACE605-CD45	K2 + IL33 KD	KC;RIK-shIl33.668 (On Dox)	6	2469	7197
Immune	DACD349_CD45+	K3 Control	KC;RIK-shIl33.668 (Off Dox)	5	705	3876
Immune	DACE610-CD45	K3 Control	KC;RIK-shIl33.668 (Off Dox)	6	1555	3222

Immune	DACD345_CD45+	K3 + IL33 KD	KC;RIK-shIl33.668 (On Dox)	5	228	3085
Immune	DACD347_CD45+	K3 + IL33 KD	KC;RIK-shIl33.668 (On Dox)	5	1110	3320
Immune	DACE607-CD45	K3 + IL33 KD	KC;RIK-shIl33.668 (On Dox)	6	1496	5020
Immune	DACE614-CD45	K3 + IL33 KD	KC;RIK-shIl33.327 (On Dox)	6	1908	3807

scRNA-seq Cohorts and QC. Samples were assigned to one of three cohorts addressing broad questions, as well as groups for pooling prior to cluster-based filtering. Cell number and mean library size reflect the final filtered count matrix for each sample.

Table S13.

Analysis	# HVGs	# PCs	Variance Explained	Samples Included
Pre-malignant to malignant (K1–K6)	4000	51	41.6 %	DACD351_Kate_plus; DACD394_Kate_plus; DACD406_Kate_plus; DACD404_Kate_plus; DACD407_Kate_plus; DAC_DI143_Epi; DACD482_Kate_plus; DAC_C301-EPI_1; DAC_C301-EPI_2; Ag-PDAC-PT-Kate; Ag-Lung-Mets-Kate; DAC_D020_p5_Epi; DACC963PT_Kate_plus; DACC963LIVERmet; DACC963_mKate_plus
Pre-malignant scATAC integration (K1–K3, K5)	4000	49	41.7 %	DACD351_Kate_plus; DACD394_Kate_plus; DACD406_Kate_plus; DACD404_Kate_plus; DACD407_Kate_plus; DAC_DI143_Epi; DACD482_Kate_plus; DAC_D020_p5_Epi; DACC963PT_Kate_plus; DACC963LIVERmet; DACC963_mKate_plus
Pre-malignant Immune integration (K1–K3)	*	42	23.0 %	DACD406_Kate_plus; DACD351_Kate_plus; DACD404_Kate_plus; DACD407_Kate_plus; DAC_DI143_Epi; DACD482_Kate_plus
Regeneration Immune Integration (N1, N2)	*	63	35.9 %	DACD511_Kate_plus; DACD550_kate_plus; DAC_B530-Kate+; DACD403_Kate_plus

Neuroendocrine and Tuft (Clusters 9 and 13)	4000	50**	35.7 %	All Progression Cohort
Acinar-to-Ductal Metaplasia (Clusters 2, 7, 3 and 12)	4000	88	50.4 %	All Progression Cohort
Malignant (K5, K6)	4000	57	42.8 %	Ag-PDAC-PT-Kate; Ag-Lung-Mets-Kate; DAC_D020_p5_Epi; DACC963PT_Kate_plus; DACC963LIVERmet; DACC963_mKate_plus

Epithelial scRNA-seq Analysis Groups. *All genes were included for these samples so that heterogeneity driven by lowly expressed receptors or ligands was preserved. **50 PCs (occurring before the knee point) were selected to improve separation of rare cell types in visualization.

Table S14.

scRNA-seq Immune Genes, provided as a separate supplementary file.

Table S15.

**Analysis**	**# HVGs**	**# PCs**	**Cell types included**
T cell, NK cell, ILC subset	4000	50	CD4_T ,CD8_T, T, gdT, ILC (3657 cells)
Myeloid subset	4000	84	TAM, Gr-MDC, mast, DC (5419 cells)
B cell subset	4000	30	B, plasma (1985 cells)

Immune scRNA-seq analysis groups. Number of HVGs and PCs are those used in processing for Calligraphy analysis.

Table S16.

Sample ID	Condition	Genotype	Paired scRNA-seq Sample ID
DACD408_b_mKATE2_ATAC	K1	KC;RIK	N/A
DACD404_b_mKATE2_ATAC	K2	KC;RIK	DACD404_Kate_plus
DACE270_Epi	K3	KC;RIK	N/A
DACE271_Epi	K3	KC;RIK	N/A
DACC963_PT_B_mKate	K5	KPflC;RIK	DACC963PT_Kate_plus
DAC_D020_p5_Epi_ATAC	K5	KPflC;RIK	DAC_D020_p5_Epi

scATAC-seq sample pairings.

Table S17.

**Cell Type**	**Markers**
Acinar (differentiated)	*Ptf1a, Bhlha15, Cpa1, Nr5a2*
Ductal	*Krt19**, Sox9***
Nes^{+} Progenitor	*Nes, Cd44, Vim*
Tuft*	*Vil1*
Neuroendocrine (NE)	*Chga, Chgb, Neurod1, Ppy, Sst*
Gastric	*Gkn3, Tff2, Tff1, Agr2*

scATAC-seq annotation strategy. *Due to low accessibility captured near cell-state markers (for example, *Dclk1*), tuft cells in scATAC-seq data are mainly identified by genome-wide similarity to tuft cells identified in scRNA-seq (see "Identifying and integrating pancreas metacells"). **Broadly accessible in metaplastic cells.

Table S18.

Cognate R-L pairs, provided as a separate supplementary file.

References and Notes

1. B. Vogelstein, N. Papadopoulos, V. E. Velculescu, S. Zhou, L. A. Diaz Jr, K. W. Kinzler, Cancer genome landscapes. *Science*. **339**, 1546–1558 (2013).
2. I. Martincorena, A. Roshan, M. Gerstung, P. Ellis, P. Van Loo, S. McLaren, D. C. Wedge, A. Fullam, L. B. Alexandrov, J. M. Tubio, L. Stebbings, A. Menzies, S. Widaa, M. R. Stratton, P. H. Jones, P. J. Campbell, Tumor evolution. High burden and pervasive positive selection of somatic mutations in normal human skin. *Science*. **348**, 880–886 (2015).
3. N. Wijewardhane, L. Dressler, F. D. Ciccarelli, Normal Somatic Mutations in Cancer Transformation. *Cancer Cell*. **39**, 125–129 (2021).
4. D. Hanahan, Hallmarks of Cancer: New Dimensions. *Cancer Discov.* **12**, 31–46 (2022).
5. A. S. Nam, R. Chaligne, D. A. Landau, Integrating genetic and non-genetic determinants of cancer evolution by single-cell multi-omics. *Nat. Rev. Genet.* **22**, 3–18 (2021).
6. C. Guerra, A. J. Schuhmacher, M. Cañamero, P. J. Grippo, L. Verdaguer, L. Pérez-Gallego, P. Dubus, E. P. Sandgren, M. Barbacid, Chronic pancreatitis is essential for induction of pancreatic ductal adenocarcinoma by K-Ras oncogenes in adult mice. *Cancer Cell*. **11**, 291–302 (2007).
7. C. Carrière, A. L. Young, J. R. Gunn, D. S. Longnecker, M. Korc, Acute pancreatitis markedly accelerates pancreatic cancer progression in mice expressing oncogenic Kras. *Biochem. Biophys. Res. Commun.* **382**, 561–565 (2009).
8. A. B. Lowenfels, P. Maisonneuve, E. P. DiMagno, Y. Elitsur, L. K. Gates, J. Perrault, D. C. Whitcomb, Hereditary Pancreatitis and the Risk of Pancreatic Cancer. *J. Natl. Cancer Inst.* **89**, 442–446 (1997).
9. L. M. Coussens, Z. Werb, Inflammation and cancer. *Nature*. **420**, 860–867 (2002).
10. V. Giroux, A. K. Rustgi, Metaplasia: tissue injury adaptation and a precursor to the dysplasia-cancer sequence. *Nat. Rev. Cancer*. **17**, 594–604 (2017).
11. R. Maddipati, B. Z. Stanger, Pancreatic Cancer Metastases Harbor Evidence of Polyclonality. *Cancer Discovery*. **5** (2015), pp. 1086–1097.
12. S. R. Torborg, Z. Li, J. E. Chan, T. Tammela, Cellular and molecular mechanisms of plasticity in cancer. *Trends Cancer Res.* (2022), doi:10.1016/j.trecan.2022.04.007.
13. W. A. Flavahan, E. Gaskell, B. E. Bernstein, Epigenetic plasticity and the hallmarks of cancer. *Science*. **357** (2017), doi:10.1126/science.aal2380.
14. M. A. Dawson, The cancer epigenome: Concepts, challenges, and therapeutic opportunities. *Science*. **355**, 1147–1152 (2017).
15. W. Xie, M. D. Schultz, R. Lister, Z. Hou, N. Rajagopal, P. Ray, J. W. Whitaker, S. Tian, R. D. Hawkins, D. Leung, H. Yang, T. Wang, A. Y. Lee, S. A. Swanson, J. Zhang, Y. Zhu, A. Kim, J. R. Nery, M. A. Urich, S. Kuan, C.-A. Yen, S. Klugman, P. Yu, K. Suknuntha, N. E.

Propson, H. Chen, L. E. Edsall, U. Wagner, Y. Li, Z. Ye, A. Kulkarni, Z. Xuan, W.-Y. Chung, N. C. Chi, J. E. Antosiewicz-Bourget, I. Slukvin, R. Stewart, M. Q. Zhang, W. Wang, J. A. Thomson, J. R. Ecker, B. Ren, Epigenomic analysis of multilineage differentiation of human embryonic stem cells. *Cell*. **153**, 1134–1148 (2013).

16. C. A. Gifford, M. J. Ziller, H. Gu, C. Trapnell, J. Donaghey, A. Tsankov, A. K. Shalek, D. R. Kelley, A. A. Shishkin, R. Issner, X. Zhang, M. Coyne, J. L. Fostel, L. Holmes, J. Meldrim, M. Guttman, C. Epstein, H. Park, O. Kohlbacher, J. Rinn, A. Gnirke, E. S. Lander, B. E. Bernstein, A. Meissner, Transcriptional and epigenetic dynamics during specification of human embryonic stem cells. *Cell*. **153**, 1149–1163 (2013).

17. L. M. LaFave, V. K. Kartha, S. Ma, K. Meli, I. Del Priore, C. Lareau, S. Naranjo, P. M. K. Westcott, F. M. Duarte, V. Sankar, Z. Chiang, A. Brack, T. Law, H. Hauck, A. Okimoto, A. Regev, J. D. Buenrostro, T. Jacks, Epigenomic State Transitions Characterize Tumor Progression in Mouse Lung Adenocarcinoma. *Cancer Cell*. **38**, 212-228.e13 (2020).

18. N. D. Marjanovic, M. Hofree, J. E. Chan, D. Canner, K. Wu, M. Trakala, G. G. Hartmann, O. C. Smith, J. Y. Kim, K. V. Evans, A. Hudson, O. Ashenberg, C. B. M. Porter, A. Bejnood, A. Subramanian, K. Pitter, Y. Yan, T. Delorey, D. R. Phillips, N. Shah, O. Chaudhary, A. Tsankov, T. Hollmann, N. Rekhtman, P. P. Massion, J. T. Poirier, L. Mazutis, R. Li, J.-H. Lee, A. Amon, C. M. Rudin, T. Jacks, A. Regev, T. Tammela, Emergence of a High-Plasticity Cell State during Lung Cancer Evolution. *Cancer Cell*. **38**, 229-246.e13 (2020).

19. P. Storz, Acinar cell plasticity and development of pancreatic ductal adenocarcinoma. *Nat. Rev. Gastroenterol. Hepatol.* **14**, 296–304 (2017).

20. C. Guerra, M. Collado, C. Navas, A. J. Schuhmacher, I. Hernández-Porras, M. Cañamero, M. Rodriguez-Justo, M. Serrano, M. Barbacid, Pancreatitis-induced inflammation contributes to pancreatic cancer by inhibiting oncogene-induced senescence. *Cancer Cell*. **19**, 728–739 (2011).

21. S. Y. Gidekel Friedlander, G. C. Chu, E. L. Snyder, N. Girnius, G. Dibelius, D. Crowley, E. Vasile, R. A. DePinho, T. Jacks, Context-dependent transformation of adult pancreatic cells by oncogenic K-Ras. *Cancer Cell*. **16**, 379–389 (2009).

22. J. P. M. Iv, D. A. Cano, S. Sekine, S. C. Wang, M. Hebrok, β-catenin blocks Kras-dependent reprogramming of acini into pancreatic cancer precursor lesions in mice. *J. Clin. Invest.* **120**, 508–520 (2 2010).

23. D. Alonso-Curbelo, Y.-J. Ho, C. Burdziak, J. L. V. Maag, J. P. Morris 4th, R. Chandwani, H.-A. Chen, K. M. Tsanov, F. M. Barriga, W. Luan, N. Tasdemir, G. Livshits, E. Azizi, J. Chun, J. E. Wilkinson, L. Mazutis, S. D. Leach, R. Koche, D. Pe'er, S. W. Lowe, A gene-environment-induced epigenetic program initiates tumorigenesis. *Nature*. **590**, 642–648 (2021).

24. E. Del Poggetto, I.-L. Ho, C. Balestrieri, E.-Y. Yen, S. Zhang, F. Citron, R. Shah, D. Corti, G. R. Diaferia, C.-Y. Li, S. Loponte, F. Carbone, Y. Hayakawa, G. Valenti, S. Jiang, L. Sapio, H. Jiang, P. Dey, S. Gao, A. K. Deem, S. Rose-John, W. Yao, H. Ying, A. D. Rhim,

G. Genovese, T. P. Heffernan, A. Maitra, T. C. Wang, L. Wang, G. F. Draetta, A. Carugo, G. Natoli, A. Viale, Epithelial memory of inflammation limits tissue damage while promoting pancreatic tumorigenesis. *Science*. **373**, eabj0486 (2021).

25. Y. Li, Y. He, J. Peng, Z. Su, Z. Li, B. Zhang, J. Ma, M. Zhuo, D. Zou, X. Liu, X. Liu, W. Wang, D. Huang, M. Xu, J. Wang, H. Deng, J. Xue, W. Xie, X. Lan, M. Chen, Y. Zhao, W. Wu, C. J. David, Mutant Kras co-opts a proto-oncogenic enhancer network in inflammation-induced metaplastic progenitor cells to initiate pancreatic cancer. *Nat Cancer*. **2**, 49–65 (2021).

26. Y. Kawaguchi, B. Cooper, M. Gannon, M. Ray, R. J. MacDonald, C. V. E. Wright, The role of the transcriptional regulator Ptf1a in converting intestinal to pancreatic progenitors. *Nat. Genet.* **32**, 128–134 (2002).

27. See Materials and Methods.

28. C. B. Westphalen, Y. Takemoto, T. Tanaka, M. Macchini, Z. Jiang, B. W. Renz, X. Chen, S. Ormanns, K. Nagar, Y. Tailor, R. May, Y. Cho, S. Asfaha, D. L. Worthley, Y. Hayakawa, A. M. Urbanska, M. Quante, M. Reichert, J. Broyde, P. S. Subramaniam, H. Remotti, G. H. Su, A. K. Rustgi, R. A. Friedman, B. Honig, A. Califano, C. W. Houchen, K. P. Olive, T. C. Wang, Dclk1 Defines Quiescent Pancreatic Progenitors that Promote Injury-Induced Regeneration and Tumorigenesis. *Cell Stem Cell*. **18**, 441–455 (2016).

29. S. Sinha, Y.-Y. Fu, A. Grimont, M. Ketcham, K. Lafaro, J. A. Saglimbeni, G. Askan, J. M. Bailey, J. P. Melchor, Y. Zhong, M. G. Joo, O. Grbovic-Huezo, I.-H. Yang, O. Basturk, L. Baker, Y. Park, R. C. Kurtz, D. Tuveson, S. D. Leach, P. J. Pasricha, PanIN Neuroendocrine Cells Promote Tumorigenesis via Neuronal Cross-talk. *Cancer Res.* **77**, 1868–1879 (2017).

30. A. D. Rhim, E. T. Mirek, N. M. Aiello, A. Maitra, J. M. Bailey, F. McAllister, M. Reichert, G. L. Beatty, A. K. Rustgi, R. H. Vonderheide, S. D. Leach, B. Z. Stanger, EMT and dissemination precede pancreatic tumor formation. *Cell*. **148**, 349–361 (2012).

31. J. L. Kopp, G. von Figura, E. Mayes, F.-F. Liu, C. L. Dubois, J. P. Morris, F. C. Pan, H. Akiyama, C. V. E. Wright, K. Jensen, M. Hebrok, M. Sander, Identification of Sox9-Dependent Acinar-to-Ductal Reprogramming as the Principal Mechanism for Initiation of Pancreatic Ductal Adenocarcinoma. *Cancer Cell*. **22** (2012), pp. 737–750.

32. J. Peng, B.-F. Sun, C.-Y. Chen, J.-Y. Zhou, Y.-S. Chen, H. Chen, L. Liu, D. Huang, J. Jiang, G.-S. Cui, Y. Yang, W. Wang, D. Guo, M. Dai, J. Guo, T. Zhang, Q. Liao, Y. Liu, Y.-L. Zhao, D.-L. Han, Y. Zhao, Y.-G. Yang, W. Wu, Author Correction: Single-cell RNA-seq highlights intra-tumoral heterogeneity and malignant progression in pancreatic ductal adenocarcinoma. *Cell Res.* **29**, 777 (2019).

33. R. R. Coifman, S. Lafon, Diffusion maps. *Appl. Comput. Harmon. Anal.* **21**, 5–30 (2006).

34. S. Yang, P. He, J. Wang, A. Schetter, W. Tang, N. Funamizu, K. Yanaga, T. Uwagawa, A. R. Satoskar, J. Gaedcke, M. Bernhardt, B. M. Ghadimi, M. M. Gaida, F. Bergmann, J. Werner, T. Ried, N. Hanna, H. R. Alexander, S. P. Hussain, A Novel MIF Signaling

Pathway Drives the Malignant Character of Pancreatic Cancer by Targeting NR3C2. *Cancer Res.* **76**, 3838–3850 (2016).

35. R. A. Moffitt, R. Marayati, E. L. Flate, K. E. Volmar, S. G. H. Loeza, K. A. Hoadley, N. U. Rashid, L. A. Williams, S. C. Eaton, A. H. Chung, J. K. Smyla, J. M. Anderson, H. J. Kim, D. J. Bentrem, M. S. Talamonti, C. A. Iacobuzio-Donahue, M. A. Hollingsworth, J. J. Yeh, Virtual microdissection identifies distinct tumor- and stroma-specific subtypes of pancreatic ductal adenocarcinoma. *Nat. Genet.* **47**, 1168–1178 (2015).

36. D. L. Gibbons, C. J. Creighton, Pan-cancer survey of epithelial-mesenchymal transition markers across the Cancer Genome Atlas. *Dev. Dyn.* **247**, 555–564 (2018).

37. R. Jahan, K. Ganguly, L. M. Smith, P. Atri, J. Carmicheal, Y. Sheinin, S. Rachagani, G. Natarajan, R. E. Brand, M. A. Macha, P. M. Grandgenett, S. Kaur, S. K. Batra, Trefoil factor(s) and CA19.9: A promising panel for early detection of pancreatic cancer. *EBioMedicine*. **42**, 375–385 (2019).

38. J.-S. Roe, C.-I. Hwang, T. D. D. Somerville, J. P. Milazzo, E. J. Lee, B. Da Silva, L. Maiorino, H. Tiriac, C. M. Young, K. Miyabayashi, D. Filippini, B. Creighton, R. A. Burkhart, J. M. Buscaglia, E. J. Kim, J. L. Grem, A. J. Lazenby, J. A. Grunkemeyer, M. A. Hollingsworth, P. M. Grandgenett, M. Egeblad, Y. Park, D. A. Tuveson, C. R. Vakoc, Enhancer Reprogramming Promotes Pancreatic Cancer Metastasis. *Cell*. **170**, 875-888.e20 (2017).

39. M. Lange, V. Bergen, M. Klein, M. Setty, B. Reuter, M. Bakhti, H. Lickert, M. Ansari, J. Schniering, H. B. Schiller, D. Pe'er, F. J. Theis, CellRank for directed single-cell fate mapping. *Nat. Methods* (2022), doi:10.1038/s41592-021-01346-6.

40. V. Bergen, M. Lange, S. Peidli, F. A. Wolf, F. J. Theis, Generalizing RNA velocity to transient cell states through dynamical modeling. *Nat. Biotechnol.* **38**, 1408–1414 (2020).

41. G. La Manno, R. Soldatov, A. Zeisel, E. Braun, H. Hochgerner, V. Petukhov, K. Lidschreiber, M. E. Kastriti, P. Lönnerberg, A. Furlan, J. Fan, L. E. Borm, Z. Liu, D. van Bruggen, J. Guo, X. He, R. Barker, E. Sundström, G. Castelo-Branco, P. Cramer, I. Adameyko, S. Linnarsson, P. V. Kharchenko, RNA velocity of single cells. *Nature*. **560**, 494–498 (2018).

42. M. Ioannou, I. Serafimidis, L. Arnes, L. Sussel, S. Singh, V. Vasiliou, A. Gavalas, ALDH1B1 is a potential stem/progenitor marker for multiple pancreas progenitor pools. *Dev. Biol.* **374**, 153–163 (2013).

43. E. Mameishvili, I. Serafimidis, S. Iwaszkiewicz, M. Lesche, S. Reinhardt, N. Bölicke, M. Büttner, D. Stellas, A. Papadimitropoulou, M. Szabolcs, K. Anastassiadis, A. Dahl, F. Theis, A. Efstratiadis, A. Gavalas, Aldh1b1 expression defines progenitor cells in the adult pancreas and is required for Kras-induced pancreatic cancer. *Proc. Natl. Acad. Sci. U. S. A.* **116**, 20679–20688 (2019).

44. C. Carrière, E. S. Seeley, T. Goetze, D. S. Longnecker, M. Korc, The Nestin progenitor lineage is the compartment of origin for pancreatic intraepithelial neoplasia. *Proc. Natl. Acad. Sci. U. S. A.* **104**, 4437–4442 (2007).

45. C. Carrière, A. L. Young, J. R. Gunn, D. S. Longnecker, M. Korc, Acute pancreatitis accelerates initiation and progression to pancreatic cancer in mice expressing oncogenic Kras in the nestin cell lineage. *PLoS One*. **6**, e27725 (2011).

46. Y. Baran, A. Bercovich, A. Sebe-Pedros, Y. Lubling, A. Giladi, E. Chomsky, Z. Meir, M. Hoichman, A. Lifshitz, A. Tanay, MetaCell: analysis of single-cell RNA-seq data using K-nn graph partitions. *Genome Biol.* **20**, 206 (2019).

47. S. Persad, Z.-N. Choo, C. Dien, I. Masilionis, R. Chaligné, T. Nawy, C. C. Brown, I. Pe'er, M. Setty, D. Pe'er, SEACells: Inference of transcriptional and epigenomic cellular states from single-cell genomics data. *bioRxiv* (2022), p. 2022.04.02.486748, , doi:10.1101/2022.04.02.486748.

48. A. Subramanian, P. Tamayo, V. K. Mootha, S. Mukherjee, B. L. Ebert, M. A. Gillette, A. Paulovich, S. L. Pomeroy, T. R. Golub, E. S. Lander, J. P. Mesirov, Gene set enrichment analysis: a knowledge-based approach for interpreting genome-wide expression profiles. *Proc. Natl. Acad. Sci. U. S. A.* **102**, 15545–15550 (2005).

49. Y. Pylayeva-Gupta, K. E. Lee, C. H. Hajdu, G. Miller, D. Bar-Sagi, Oncogenic Kras-induced GM-CSF production promotes the development of pancreatic neoplasia. *Cancer Cell*. **21**, 836–847 (2012).

50. M. Efremova, M. Vento-Tormo, S. A. Teichmann, R. Vento-Tormo, CellPhoneDB: inferring cell-cell communication from combined expression of multi-subunit ligand-receptor complexes. *Nat. Protoc.* **15**, 1484–1506 (2020).

51. O. Strobel, Y. Dor, J. Alsina, A. Stirman, G. Lauwers, A. Trainor, C. F.-D. Castillo, A. L. Warshaw, S. P. Thayer, In vivo lineage tracing defines the role of acinar-to-ductal transdifferentiation in inflammatory ductal metaplasia. *Gastroenterology*. **133**, 1999–2009 (2007).

52. Y. Schlesinger, O. Yosefov-Levi, D. Kolodkin-Gal, R. Z. Granit, L. Peters, R. Kalifa, L. Xia, A. Nasereddin, I. Shiff, O. Amran, Y. Nevo, S. Elgavish, K. Atlan, G. Zamir, O. Parnas, Single-cell transcriptomes of pancreatic preinvasive lesions and cancer reveal acinar metaplastic cells' heterogeneity. *Nat. Commun.* **11**, 4516 (2020).

53. H. Gonzalez, C. Hagerling, Z. Werb, Roles of the immune system in cancer: from tumor initiation to metastatic progression. *Genes Dev.* **32**, 1267–1284 (2018).

54. U. Alon, Network motifs: theory and experimental approaches. *Nat. Rev. Genet.* **8**, 450–461 (2007).

55. S. Das, B. Shapiro, E. A. Vucic, S. Vogt, D. Bar-Sagi, Tumor Cell-Derived IL1β Promotes Desmoplasia and Immune Suppression in Pancreatic Cancer. *Cancer Res.* **80**, 1088–1101 (2020).

56. A. Alam, E. Levanduski, P. Denz, H. S. Villavicencio, M. Bhatta, L. Alhorebi, Y. Zhang, E. C. Gomez, B. Morreale, S. Senchanthisai, J. Li, S. G. Turowski, S. Sexton, S. J. Sait, P. K. Singh, J. Wang, A. Maitra, P. Kalinski, R. A. DePinho, H. Wang, W. Liao, S. I. Abrams, B. H. Segal, P. Dey, Fungal mycobiome drives IL-33 secretion and type 2 immunity in pancreatic cancer. *Cancer Cell*. **40**, 153-167.e11 (2022).

57. L. Y. Drake, H. Kita, IL-33: biological properties, functions, and roles in airway disease. *Immunol. Rev.* **278**, 173–184 (2017).

58. P. Andersson, Y. Yang, K. Hosaka, Y. Zhang, C. Fischer, H. Braun, S. Liu, G. Yu, S. Liu, R. Beyaert, M. Chang, Q. Li, Y. Cao, Molecular mechanisms of IL-33-mediated stromal interactions in cancer metastasis. *JCI Insight*. **3** (2018), doi:10.1172/jci.insight.122375.

59. A. Velez-Delgado, K. L. Donahue, K. L. Brown, W. Du, V. Irizarry-Negron, R. E. Menjivar, E. L. Lasse Opsahl, N. G. Steele, S. The, J. Lazarus, V. R. Sirihorachai, W. Yan, S. B. Kemp, S. A. Kerk, M. Bollampally, S. Yang, M. K. Scales, F. R. Avritt, F. Lima, C. A. Lyssiotis, A. Rao, H. C. Crawford, F. Bednar, T. L. Frankel, B. L. Allen, Y. Zhang, M. Pasca di Magliano, Extrinsic KRAS signaling shapes the pancreatic microenvironment through fibroblast reprogramming. *Cell. Mol. Gastroenterol. Hepatol.* **13**, 1673–1699 (2022).

60. E. Dann, N. C. Henderson, S. A. Teichmann, M. D. Morgan, J. C. Marioni, Differential abundance testing on single-cell data using k-nearest neighbor graphs. *Nat. Biotechnol.* (2021), doi:10.1038/s41587-021-01033-z.

61. M. Setty, V. Kiseliovas, J. Levine, A. Gayoso, L. Mazutis, D. Pe’er, Characterization of cell fate probabilities in single-cell data with Palantir. *Nat. Biotechnol.* **37**, 451–460 (2019).

62. J. M. Bailey, A. M. Hendley, K. J. Lafaro, M. A. Pruski, N. C. Jones, J. Alsina, M. Younes, A. Maitra, F. McAllister, C. A. Iacobuzio-Donahue, S. D. Leach, p53 mutations cooperate with oncogenic Kras to promote adenocarcinoma from pancreatic ductal cells. *Oncogene*. **35**, 4282–4288 (2016).

63. A. Malinova, L. Veghini, F. X. Real, V. Corbo, Cell lineage infidelity in PDAC progression and therapy resistance. *Front. Cell Dev. Biol.* **9**, 795251 (2021).

64. E. Azizi, A. J. Carr, G. Plitas, A. E. Cornish, C. Konopacki, S. Prabhakaran, J. Nainys, K. Wu, V. Kiseliovas, M. Setty, K. Choi, R. M. Fromme, P. Dao, P. T. McKenney, R. C. Wasti, K. Kadaveru, L. Mazutis, A. Y. Rudensky, D. Pe’er, Single-Cell Map of Diverse Immune Phenotypes in the Breast Tumor Microenvironment. *Cell*. **174**, 1293-1308.e36 (2018).

65. J. M. Granja, M. R. Corces, S. E. Pierce, S. T. Bagdatli, H. Choudhry, H. Y. Chang, W. J. Greenleaf, ArchR is a scalable software package for integrative single-cell chromatin accessibility analysis. *Nat. Genet.* **53**, 403–411 (2021).

66. N. F. Greenwald, G. Miller, E. Moen, A. Kong, A. Kagel, T. Dougherty, C. C. Fullaway, B. J. McIntosh, K. X. Leow, M. S. Schwartz, C. Pavelchek, S. Cui, I. Camplisson, O. Bar-Tal, J. Singh, M. Fong, G. Chaudhry, Z. Abraham, J. Moseley, S. Warshawsky, E. Soon, S. Greenbaum, T. Risom, T. Hollmann, S. C. Bendall, L. Keren, W. Graf, M. Angelo, D. Van

Valen, Whole-cell segmentation of tissue images with human-level performance using large-scale data annotation and deep learning. *Nat. Biotechnol.* **40**, 555–565 (2022).

67. N. M. Krah, J.-P. De La O, G. H. Swift, C. Q. Hoang, S. G. Willet, F. Chen Pan, G. M. Cash, M. P. Bronner, C. V. Wright, R. J. MacDonald, L. C. Murtaugh, The acinar differentiation determinant PTF1A inhibits initiation of pancreatic ductal adenocarcinoma. *Elife*. **4** (2015), doi:10.7554/eLife.07125.

68. C. F. Schaefer, K. Anthony, S. Krupa, J. Buchoff, M. Day, T. Hannay, K. H. Buetow, PID: the Pathway Interaction Database. *Nucleic Acids Res.* **37**, D674-9 (2009).

69. D. van Dijk, R. Sharma, J. Nainys, K. Yim, P. Kathail, A. J. Carr, C. Burdziak, K. R. Moon, C. L. Chaffer, D. Pattabiraman, B. Bierie, L. Mazutis, G. Wolf, S. Krishnaswamy, D. Pe'er, Recovering Gene Interactions from Single-Cell Data Using Data Diffusion. *Cell*. **174**, 716-729.e27 (2018).

70. M. Saborowski, A. Saborowski, J. P. Morris 4th, B. Bosbach, L. E. Dow, J. Pelletier, D. S. Klimstra, S. W. Lowe, A modular and flexible ESC-based mouse model of pancreatic cancer. *Genes Dev.* **28**, 85–97 (2014).

71. C. Fellmann, T. Hoffmann, V. Sridhar, B. Hopfgartner, M. Muhar, M. Roth, D. Y. Lai, I. A. M. Barbosa, J. S. Kwon, Y. Guan, N. Sinha, J. Zuber, An optimized microRNA backbone for effective single-copy RNAi. *Cell Rep.* **5**, 1704–1713 (2013).

72. L. E. Dow, P. K. Premsrirut, J. Zuber, C. Fellmann, K. McJunkin, C. Miething, Y. Park, R. A. Dickins, G. J. Hannon, S. W. Lowe, A pipeline for the generation of shRNA transgenic mice. *Nat. Protoc.* **7**, 374–393 (2012).

73. M. Gertsenstein, L. M. J. Nutter, T. Reid, M. Pereira, W. L. Stanford, J. Rossant, A. Nagy, Efficient generation of germ line transmitting chimeras from C57BL/6N ES cells by aggregation with outbred host embryos. *PLoS One*. **5**, e11260 (2010).

74. E. L. Jackson, N. Willis, K. Mercer, R. T. Bronson, D. Crowley, R. Montoya, T. Jacks, D. A. Tuveson, Analysis of lung tumor initiation and progression using conditional expression of oncogenic K-ras. *Genes Dev.* **15**, 3243–3248 (2001).

75. S. R. Hingorani, L. Wang, A. S. Multani, C. Combs, T. B. Deramaudt, R. H. Hruban, A. K. Rustgi, S. Chang, D. A. Tuveson, Trp53R172H and KrasG12D cooperate to promote chromosomal instability and widely metastatic pancreatic ductal adenocarcinoma in mice. *Cancer Cell*. **7**, 469–483 (2005).

76. C. Beard, K. Hochedlinger, K. Plath, A. Wutz, R. Jaenisch, Efficient method to generate single-copy transgenic mice by site-specific integration in embryonic stem cells. *genesis*. **44** (2006), pp. 23–28.

77. L. E. Dow, Z. Nasr, M. Saborowski, S. H. Ebbesen, E. Manchado, N. Tasdemir, T. Lee, J. Pelletier, S. W. Lowe, Conditional reverse tet-transactivator mouse strains for the efficient induction of TRE-regulated transgenes in mice. *PLoS One*. **9**, e95236 (2014).

78. J. R. Moffitt, D. Bambah-Mukku, S. W. Eichhorn, E. Vaughn, K. Shekhar, J. D. Perez, N. D. Rubinstein, J. Hao, A. Regev, C. Dulac, X. Zhuang, Molecular, spatial, and functional single-cell profiling of the hypothalamic preoptic region. *Science*. **362**, eaau5324 (2018).

79. J. R. Moffitt, J. Hao, G. Wang, K. H. Chen, H. P. Babcock, X. Zhuang, High-throughput single-cell gene-expression profiling with multiplexed error-robust fluorescence in situ hybridization. *Proc. Natl. Acad. Sci. U. S. A.* **113**, 11046–11051 (2016).

80. G. Wang, J. R. Moffitt, X. Zhuang, Multiplexed imaging of high-density libraries of RNAs with MERFISH and expansion microscopy. *Sci. Rep.* **8**, 4847 (2018).

81. L. Farack, S. Itzkovitz, Protocol for single-molecule fluorescence in situ hybridization for intact pancreatic tissue. *STAR Protoc.* **1**, 100007 (2020).

82. J. R. Moffitt, J. Hao, D. Bambah-Mukku, T. Lu, C. Dulac, X. Zhuang, High-performance multiplexed fluorescence in situ hybridization in culture and tissue with matrix imprinting and clearing. *Proc. Natl. Acad. Sci. U. S. A.* **113**, 14456–14461 (2016).

83. J.-R. Lin, M. Fallahi-Sichani, P. K. Sorger, Highly multiplexed imaging of single cells using a high-throughput cyclic immunofluorescence method. *Nat. Commun.* **6**, 8390 (2015).

84. K. H. Chen, A. N. Boettiger, J. R. Moffitt, S. Wang, X. Zhuang, RNA imaging. Spatially resolved, highly multiplexed RNA profiling in single cells. *Science*. **348**, aaa6090 (2015).

85. J. H. Levine, E. F. Simonds, S. C. Bendall, K. L. Davis, E.-A. D. Amir, M. D. Tadmor, O. Litvin, H. G. Fienberg, A. Jager, E. R. Zunder, R. Finck, A. L. Gedman, I. Radtke, J. R. Downing, D. Pe'er, G. P. Nolan, Data-Driven Phenotypic Dissection of AML Reveals Progenitor-like Cells that Correlate with Prognosis. *Cell*. **162**, 184–197 (2015).

86. A. T. Satpathy, J. M. Granja, K. E. Yost, Y. Qi, F. Meschi, G. P. McDermott, B. N. Olsen, M. R. Mumbach, S. E. Pierce, M. R. Corces, P. Shah, J. C. Bell, D. Jhutty, C. M. Nemec, J. Wang, L. Wang, Y. Yin, P. G. Giresi, A. L. S. Chang, G. X. Y. Zheng, W. J. Greenleaf, H. Y. Chang, Massively parallel single-cell chromatin landscapes of human immune cell development and intratumoral T cell exhaustion. *Nat. Biotechnol.* **37**, 925–936 (2019).

87. A. Gayoso, J. Shor, *JonathanShor/DoubletDetection: doubletdetection v3.0* (2020; https://zenodo.org/record/4359992).

88. C. S. Smillie, M. Biton, J. Ordovas-Montanes, K. M. Sullivan, G. Burgin, D. B. Graham, R. H. Herbst, N. Rogel, M. Slyper, J. Waldman, M. Sud, E. Andrews, G. Velonias, A. L. Haber, K. Jagadeesh, S. Vickovic, J. Yao, C. Stevens, D. Dionne, L. T. Nguyen, A.-C. Villani, M. Hofree, E. A. Creasey, H. Huang, O. Rozenblatt-Rosen, J. J. Garber, H. Khalili, A. Nicole Desch, M. J. Daly, A. N. Ananthakrishnan, A. K. Shalek, R. J. Xavier, A. Regev, Intra- and Inter-cellular Rewiring of the Human Colon during Ulcerative Colitis. *Cell*. **178** (2019), pp. 714-730.e22.

89. L. van der Maaten, Accelerating t-SNE using Tree-Based Algorithms. *J. Mach. Learn. Res.* **15**, 3221–3245 (2014).

90. S. Nowotschin, M. Setty, Y.-Y. Kuo, V. Liu, V. Garg, R. Sharma, C. S. Simon, N. Saiz, R. Gardner, S. C. Boutet, D. M. Church, P. A. Hoodless, A.-K. Hadjantonakis, D. Pe'er, The emergent landscape of the mouse gut endoderm at single-cell resolution. *Nature*. **569**, 361–367 (2019).

91. A. T. L. Lun, K. Bach, J. C. Marioni, Pooling across cells to normalize single-cell RNA sequencing data with many zero counts. *Genome Biol.* **17**, 75 (2016).

92. Y. Zhang, T. Liu, C. A. Meyer, J. Eeckhoute, D. S. Johnson, B. E. Bernstein, C. Nusbaum, R. M. Myers, M. Brown, W. Li, X. S. Liu, Model-based analysis of ChIP-Seq (MACS). *Genome Biol.* **9**, R137 (2008).

93. M. I. Love, W. Huber, S. Anders, Moderated estimation of fold change and dispersion for RNA-seq data with DESeq2. *Genome Biol.* **15**, 550 (2014).

94. F. Pedregosa, G. Varoquaux, A. Gramfort, V. Michel, B. Thirion, O. Grisel, M. Blondel, P. Prettenhofer, R. Weiss, V. Dubourg, Others, Scikit-learn: Machine learning in Python. *the Journal of machine Learning research*. **12**, 2825–2830 (2011).

95. H. Li, Tabix: fast retrieval of sequence features from generic TAB-delimited files. *Bioinformatics*. **27**, 718–719 (2011).

96. D. Papailiopoulos, A. Kyrillidis, C. Boutsidis, "Provable deterministic leverage score sampling" in *Proceedings of the 20th ACM SIGKDD international conference on Knowledge discovery and data mining* (Association for Computing Machinery, New York, NY, USA, 2014), *KDD '14*, pp. 997–1006.

97. M. Kanehisa, S. Goto, KEGG: kyoto encyclopedia of genes and genomes. *Nucleic Acids Res.* **28**, 27–30 (2000).

98. A. Hagberg, P. Swart, D. S Chult, "Exploring network structure, dynamics, and function using networkx" (LA-UR-08-05495; LA-UR-08-5495, Los Alamos National Lab. (LANL), Los Alamos, NM (United States), 2008), (available at https://www.osti.gov/biblio/960616).

99. A. Lancichinetti, F. Radicchi, J. J. Ramasco, S. Fortunato, Finding statistically significant communities in networks. *PLoS One*. **6**, e18961 (2011).

100. N. G. Steele, E. S. Carpenter, S. B. Kemp, V. R. Sirihorachai, S. The, L. Delrosario, J. Lazarus, E.-A. D. Amir, V. Gunchick, C. Espinoza, S. Bell, L. Harris, F. Lima, V. Irizarry-Negron, D. Paglia, J. Macchia, A. K. Y. Chu, H. Schofield, E.-J. Wamsteker, R. Kwon, A. Schulman, A. Prabhu, R. Law, A. Sondhi, J. Yu, A. Patel, K. Donahue, H. Nathan, C. Cho, M. A. Anderson, V. Sahai, C. A. Lyssiotis, W. Zou, B. L. Allen, A. Rao, H. C. Crawford, F. Bednar, T. L. Frankel, M. Pasca di Magliano, Multimodal Mapping of the Tumor and Peripheral Blood Immune Landscape in Human Pancreatic Cancer. *Nat Cancer*. **1**, 1097–1112 (2020).
